# Herbal medicine for the treatment of chronic cough: a systematic review and meta-analysis

**DOI:** 10.3389/fphar.2023.1230604

**Published:** 2023-10-17

**Authors:** Boram Lee, Chan-Young Kwon, Hyo-Weon Suh, Ye Ji Kim, Kwan-Il Kim, Beom-Joon Lee, Jun-Hwan Lee

**Affiliations:** ^1^ KM Science Research Division, Korea Institute of Oriental Medicine, Daejeon, Republic of Korea; ^2^ Department of Oriental Neuropsychiatry, Dong-eui University College of Korean Medicine, Busan, Republic of Korea; ^3^ Health Policy Research Team, Division of Healthcare Research, National Evidence-based Healthcare Collaborating Agency, Seoul, Republic of Korea; ^4^ Department of Korean Pediatrics, Kyung Hee University Medical Center, Seoul, Republic of Korea; ^5^ Division of Allergy, Immune and Respiratory System, Department of Internal Medicine, College of Korean Medicine, Kyung Hee Medical Center, Kyung Hee University, Seoul, Republic of Korea; ^6^ Korean Medicine Life Science, University of Science and Technology (UST), Campus of Korea Institute of Oriental Medicine, Daejeon, Republic of Korea

**Keywords:** herbal medicine, chronic cough, cough, systematic review, East Asian traditional medicine

## Abstract

**Objectives:** Chronic cough is a frequent condition worldwide that significantly impairs quality of life. Herbal medicine (HM) has been used to treat chronic cough due to the limited effectiveness of conventional medications. This study aimed to summarize and determine the effects of HM on patients with chronic cough.

**Methods:** A comprehensive search of 11 databases was conducted to find randomized controlled clinical trials (RCTs) that reported the effects of HM for patients with chronic cough on 16 March 2023. The primary outcome was cough severity, and the secondary outcomes included cough-related quality of life, cough frequency, total effective rate (TER), and cough recurrence rate. The methodological quality of the included studies was assessed using the Cochrane risk of bias tool, and the certainty of the evidence for effect estimates was assessed using the Grading of Recommendations, Assessment, Development, and Evaluations tool.

**Results:** A total of 80 RCTs comprising 7,573 patients were included. When HM was used as an alternative therapy to conventional medication, there were inconsistent results in improving cough severity. However, HM significantly improved cough-related quality of life and TER and significantly lowered the cough recurrence rate compared with conventional medication. When used as an add-on therapy to conventional medication, HM significantly improved cough severity, cough-related quality of life, and TER and significantly lowered the recurrence rate. In addition, HM had a significantly lower incidence of adverse events when used as an add-on or alternative therapy to conventional medication. The subgroup analysis according to age and cause of cough also showed a statistically consistent correlation with the overall results. The certainty of the evidence for the effect of HM was generally moderate to low due to the risk of bias in the included studies.

**Conclusion:** HM may improve cough severity and cough-related quality of life, and lower the cough recurrence rate and incidence of adverse events in patients with chronic cough. However, due to the high risk of bias and clinical heterogeneity of the included studies, further high-quality placebo-controlled clinical trials should be conducted using a validated and objective assessment tool.

**Systematic Review Registration:** https://www.crd.york.ac.uk/prospero/display_record.php?ID=CRD42023418736, CRD42023418736.

## 1 Introduction

Chronic cough refers to a cough lasting more than 8 weeks in adults and children over the age of 15 or more than 4 weeks in children under the age of 15 (Chang and Glomb, 2006; Irwin et al., 2006). Chronic cough is a common problem that affects 10% of the general population worldwide (Song et al., 2015), and chronic cough imposes a severe socio-economic burden by directly impacting the quality of life and decreasing productivity (Won et al., 2020; Kubo et al., 2021). The most common causes of chronic cough are upper airway cough syndrome (UACS), asthma, cough variant asthma (CVA), gastroesophageal reflux disease (GERD), and eosinophilic bronchitis. Empirical treatments based on common causes through taking the medical history and physical examination have been conducted in clinical settings (Perotin et al., 2018; Kaplan, 2019). However, unexplained or refractory chronic cough, which has an unknown etiology after a thorough investigation and therapeutic trials of common cough-trigger conditions, has been reported in nearly half of all cases of chronic cough (Gibson et al., 2016). Nonspecific chronic cough with no history, symptoms, signs, or laboratory findings is also common (Song et al., 2018). In addition, according to a survey study, a suggested diagnosis for the cough was given in only 53% of the 1,120 patients suffering from chronic cough, and more than 90% reported limited or no effectiveness of conventional medications (Chamberlain et al., 2015).

East Asian traditional medicine (EATM), including herbal medicine (HM), has frequently been used by patients with chronic cough who were difficult to diagnose or were not effectively treated with conventional medication. HM especially has the potential to be a treatment for nonspecific or unexplained chronic cough as well as specific coughs based on the characteristics of multi-components and multi-targets (Wang et al., 2012). Accordingly, clinical trials to evaluate the effects of specific HMs on nonspecific chronic cough are being conducted (Kim et al., 2016; Lee et al., 2023). Although the effects of HM were previously summarized through systematic reviews, these were mainly limited to common causes of chronic cough, such as CVA and UACS (Jiang et al., 2016; Chen et al., 2021). To the best of our knowledge, no study has systematically summarized the effects of HM on chronic cough regardless of the causative disease. Therefore, this study aimed to evaluate the certainty of evidence by comprehensively synthesizing the effects of HM on chronic cough, regardless of the causative disease and age, to aid the decision-making of clinicians, patients, researchers, and policymakers.

## 2 Methods

### 2.1 Protocol registration

The protocol of this systematic review was registered to the International Prospective Register of Systematic Reviews (PROSPERO) (identifying number: CRD42023418736; registration date: 30 April 2023). There were no deviations from the protocol.

### 2.2 Eligibility criteria

1) Study design: Only parallel-group randomized controlled clinical trials (RCTs) published in journals were included.
2) Population: Studies involving populations with chronic cough lasting longer than 8 weeks in adults (≥15 years old) or 4 weeks in children (<15 years old) were included (Chang and Glomb, 2006; Irwin et al., 2006), regardless of age, sex, race, and nationality. Studies not describing the cough period were excluded since whether the study met the definition of chronic cough could not be determined.
3) Treatment intervention: Studies using oral HM based on the EATM theory as a monotherapy or add-on therapy to conventional medication for chronic cough were included as a treatment intervention. Studies in which the composition of botanical drugs in HM was not specified were excluded. Conventional medications for chronic cough in this review included any medication used for treating chronic cough in the included studies, such as antitussive expectorants, bronchodilators, and codeine phosphate.
4) Control intervention: Only conventional medication, waitlist, and placebo HM were eligible as a control intervention. Studies using EATM therapies such as HM, acupuncture, and moxibustion as a control intervention were excluded.
5) Outcomes: Only studies reporting at least one of the following primary and secondary outcomes were included. The primary outcome was post-treatment cough severity, measured by methods such as the cough symptom score (CSS), cough visual analog scale (VAS), or simplified cough score (SCS) (Wang et al., 2019). The secondary outcomes included 1) post-treatment cough-related quality of life, measured by methods such as the Leicester cough questionnaire (LCQ), 2) post-treatment cough frequency, 3) total effective rate (TER) based on cough symptom improvement, 4) cough recurrence rate, and 5) incidence of adverse events during the trial period.
6) Others: There were no publication language restrictions.

### 2.3 Search sources and strategy

The following 11 electronic databases were searched for published studies on 16 March 2023: Medline, EMBASE, Cochrane Central Register of Controlled Trials, the Allied and Complementary Medicine Database, China National Knowledge Infrastructure, Wanfang data, Oriental Medicine Advanced Searching Integrated System, Korean Medical Database, ScienceON, Research Information Sharing Service, and Citation Information by National Institute of Informatics. Reference lists of relevant articles and trial registries, such as ClinicalTrials.gov, were reviewed to identify eligible studies. Search strategies were consulted with experts in respiratory medicine and systematic reviews. The search strategies for all the databases are described in Supplementary Material S1.

### 2.4 Study selection and data collection

The study selection process was independently performed by two authors (BL. and C-YK), and data extraction was independently performed by four authors (BL, C-YK, H-WS, and YK). Any disagreements between them were resolved by discussions with other researchers.

The bibliographic information of the studies identified from the databases or other sources was imported into EndNote 20 (Clarivate Analytics, PA, United States), and the titles and abstracts were reviewed after removing any duplicate studies. Full texts were searched for eligible studies, and the final included studies were confirmed after reviewing the retrieved studies. For the final included studies, the following data were extracted using a pilot-tested Excel form (Excel 365, Microsoft, Redmond, WA, United States): study characteristics (first author, publication year, publication language, the country where the study was conducted, study setting, and funding source), population (sample size, mean age and cough period, and pattern identification), details of HM (name, dosage form, composition, manufacturing company, and administration period), control intervention, the outcome of interest, results, and adverse events.

### 2.5 Risk of bias assessment

The risk of bias in the included studies was assessed by Cochrane’s risk of bias tool (Higgins et al., 2011). The risk of selection, performance, detection, attrition, reporting, and other biases in the individual studies were rated as “low,” “unclear,” or “high.” Other bias items were evaluated based on statistical or clinical heterogeneity of the baseline characteristics between the treatment and control groups.

### 2.6 Data analysis and synthesis

All of the included studies were qualitatively analyzed. In cases where at least two studies used the same treatment and control intervention and had the same outcome measure, a meta-analysis was conducted using Review Manager software, version 5.4 (Cochrane, London, UK). Continuous data are presented using the mean differences (MDs) with their 95% confidence intervals (CIs), while categorical data are presented using the risk ratio (RR) with their 95% CIs.

Heterogeneity between the studies in terms of effect measures was assessed using the I^2^ statistic, which expresses the proportion of variability in a meta-analysis that is explained by between-trial heterogeneity rather than by sampling error. The I^2^ values were calculated as (Q—df)/Q × 100%, where Q is the Cochran’s homogeneity test statistic, and df is the degrees of freedom (the number of trials minus 1) (Higgins and Thompson, 2002; Thorlund et al., 2012). We considered I^2^ values greater than 50% and 75% to be indicative of substantial and high heterogeneity, respectively. Considering the unavoidable clinical heterogeneity of the HM intervention, such as that caused by botanical drugs content, dose, and administration period, between the included studies, the results were pooled using a random-effect model, which assumes that the true effect could vary from study to study due to the differences among studies (Borenstein et al., 2010).

To interpret the heterogeneity in the meta-analysis results if there was more than one study included in each subgroup, subgroup analyses were conducted according to the age of the participants (children, adults, and both) and the cause of the cough. Through subgroup analysis, the changes in I^2^ values and consistency with the overall meta-analysis results were examined. To identify the robustness of the meta-analysis result, sensitivity analyses were performed by excluding studies with high risks of bias or outliers. In cases where there were a sufficient number of studies (≥10) included in the meta-analysis, the evidence of publication bias was assessed by the symmetry of the funnel plot and Egger’s test.

### 2.7 Certainty assessment

The certainty of the evidence for the meta-analysis findings was assessed by the Grading of Recommendations Assessment, Development, and Evaluation (GRADE) approach (Balshem et al., 2011). To evaluate the certainty of the evidence, the risk of bias of the studies included in the analysis; the indirectness, inconsistency, and imprecision of the effect estimate; and the risk of publication bias were evaluated. For each finding, the certainty of the evidence was rated as “High,” “Moderate,” “Low,” or “Very low."

## 3 Results

### 3.1 Study selection and characteristics

**FIGURE 1:**
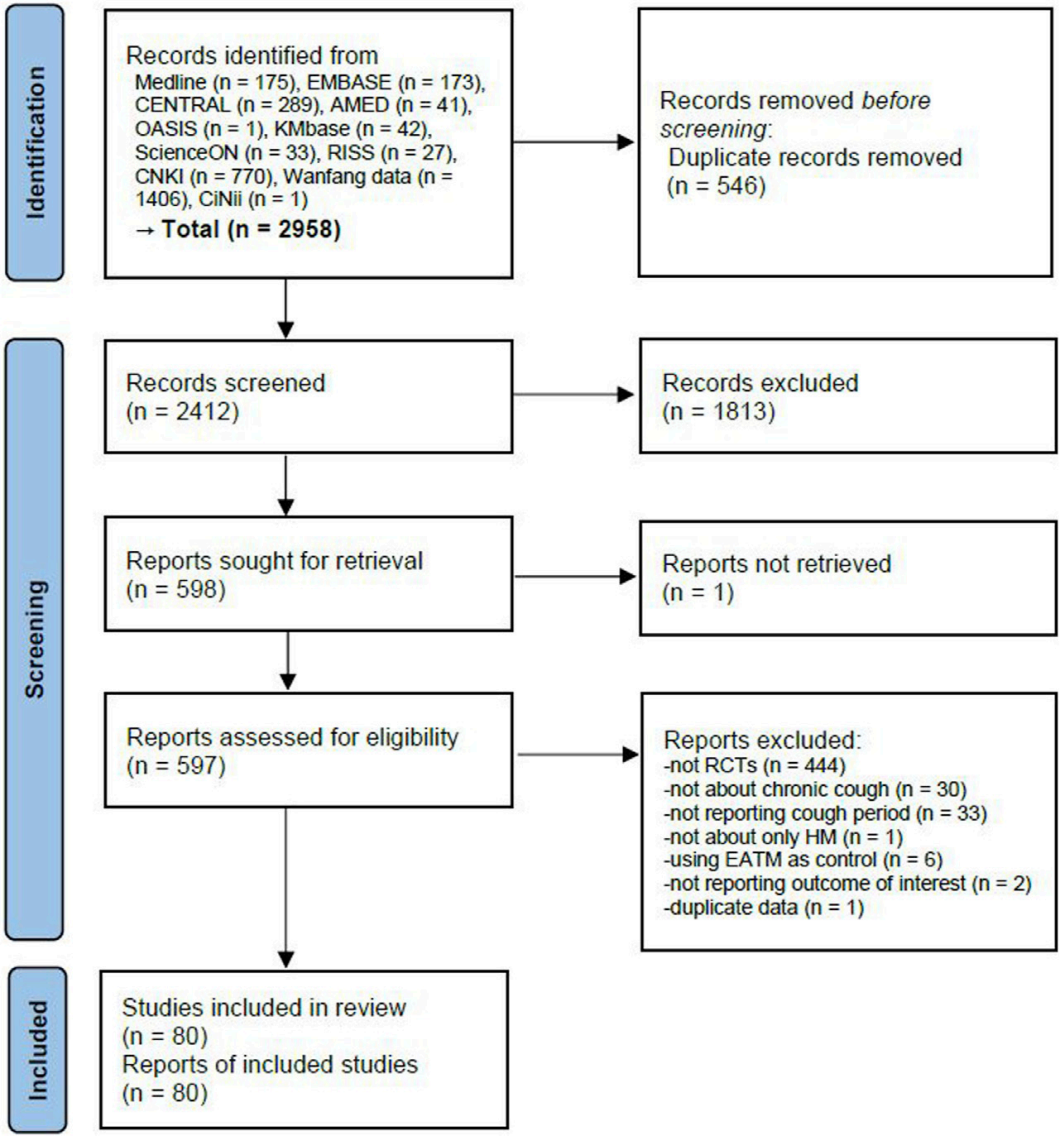
PRISMA 2020 flow diagram. EATM, East Asian traditional medicine; HM, herbal medicine; RCT, randomized controlled clinical trial.

A total of 2,958 articles were identified through the database search. After removing 546 duplicate articles, 1813 articles were excluded through the title and abstract review. As a result, 597 studies underwent full-text review, except for one study where the full text was not retrieved. After the full-text review, 517 studies were excluded for the following reasons: not an RCT (*n* = 444), not about chronic cough (*n* = 30), not reporting cough period (*n* = 33), not about HM (*n* = 1), using EATM as the control group intervention (n = 6), not reporting the outcome of interest (*n* = 2), and duplicate data (*n* = 1) (Supplementary Material S2). Finally, a total of 80 studies involving 7,573 participants were included in this review (Han et al., 2008; Chen et al., 2009; Shi et al., 2010; Zhou, 2011; Deng, 2012; Liu and Zhang, 2012; Wang, 2012; Xie et al., 2012; Yang and Yang, 2012; Zhu, 2012; Gan and Zhu, 2013; Lu et al., 2013; Chen, 2014; Cui, 2014; Lin, 2014; Lu and Pan, 2014; Yu et al., 2014; Chen, 2016; Cui et al., 2016; Wang and Zang, 2016; Wang and Zhou, 2016; Zhang et al., 2016; Fan, 2017; Gao et al., 2017; Ge et al., 2017; Shen et al., 2017; Xie, 2017; Xu, 2017; Zhou et al., 2017; Zhu, 2017; Li, 2018; Yan et al., 2018; Zhou, 2018; Chen et al., 2019; Li, 2019; Shen and Huang, 2019; Wang and Li, 2019; Wu et al., 2019; Yang et al., 2019; Gu, 2020; Hui and Dong, 2020; Lai, 2020; Li, 2020; Liu, 2020; Meng et al., 2020; Niu et al., 2020; Sun and Wei, 2020; Tan and Zheng, 2020; Tang et al., 2020; Wang, 2020; Wu and Li, 2020; Yang et al., 2020; Yi and Zheng, 2020; Zhang and Chen, 2020; Zhang H. et al., 2021; Chen, 2021; Chen and Chen, 2021; Diao, 2021; He, 2021; Huang, 2021; Li et al., 2021; Luan et al., 2021; Xia, 2021; Zhou et al., 2021; Zhao L. et al., 2022; Zhao Y. et al., 2022; Gong, 2022; Guan, 2022; Ji, 2022; Li et al., 2022; Liang et al., 2022; Liu, 2022; Lyu et al., 2022; Song, 2022; Wang and Xu, 2022; Yang et al., 2022; Zhang and Wang, 2022; Zhao and Tuo, 2022; Zhou et al., 2022; Tian, 2023) (Figure 1).

One study was conducted in Korea and published in English (Lyu et al., 2022); the others were conducted in China and published in Chinese. The study setting of one study was a family planning service station (Wang and Zhou, 2016), and the remaining settings were clinics. A total of 21 studies reported funding sources (Shi et al., 2010; Lu et al., 2013; Lu and Pan, 2014; Chen, 2016; Zhang et al., 2016; Gao et al., 2017; Ge et al., 2017; Shen et al., 2017; Zhou et al., 2017; Yang et al., 2019; Meng et al., 2020; Tang et al., 2020; Wang, 2020; Wu and Li, 2020; Zhang and Chen, 2020; Zhang H. et al., 2021; Li et al., 2021; Zhou et al., 2021; Lyu et al., 2022; Zhang and Wang, 2022; Zhao and Tuo, 2022). A total of 27 studies were conducted on children (Chen et al., 2009; Zhou, 2011; Liu and Zhang, 2012; Yu et al., 2014; Chen, 2016; Cui et al., 2016; Ge et al., 2017; Zhou et al., 2017; Li, 2018; Yan et al., 2018; Li, 2019; Shen and Huang, 2019; Niu et al., 2020; Wang, 2020; Zhang and Chen, 2020; Chen and Chen, 2021; Diao, 2021; He, 2021; Li et al., 2021; Luan et al., 2021; Zhao L. et al., 2022; Gong, 2022; Li et al., 2022; Liang et al., 2022; Yang et al., 2022; Zhao and Tuo, 2022; Tian, 2023), 3 studies included both children and adults (Zhang et al., 2016; Fan, 2017; Wu and Li, 2020), and the remaining 50 studies were conducted on adults. Only 30 studies (37.5%) described a cause for the cough, including post-respiratory infection in 5 studies (Zhou, 2011; Li, 2019; Shen and Huang, 2019; Niu et al., 2020; Diao, 2021); GERD (Deng, 2012; Gao et al., 2017; Lyu et al., 2022), UACS (Chen et al., 2009; Chen et al., 2019; Luan et al., 2021), and unexplained chronic cough (Wang, 2012; Chen, 2014; Zhang et al., 2016) in 3 studies each; and chronic bronchitis (Song, 2022), CVA (Cui et al., 2016), and nonspecific chronic chough (Chen and Chen, 2021) in 1 study each. There were also 13 studies targeting a mixed population, including patients with various causes of the cough (Han et al., 2008; Cui, 2014; Lu and Pan, 2014; Yu et al., 2014; Chen, 2016; Xu, 2017; Zhu, 2017; Lai, 2020; Wang, 2020; Yang et al., 2020; Yi and Zheng, 2020; Li et al., 2021; Zhou et al., 2021). One study was a 3-arm RCT comparing HM plus conventional medication, HM, and conventional medication (Zhang et al., 2016). The remaining studies were 2-arm RCTs, 33 comparing HM and conventional medication (Han et al., 2008; Shi et al., 2010; Deng, 2012; Liu and Zhang, 2012; Wang, 2012; Xie et al., 2012; Yang and Yang, 2012; Zhu, 2012; Lu et al., 2013; Yu et al., 2014; Fan, 2017; Xie, 2017; Zhu, 2017; Li, 2018; Wang and Li, 2019; Gu, 2020; Hui and Dong, 2020; Lai, 2020; Li, 2020; Liu, 2020; Meng et al., 2020; Tang et al., 2020; Wu and Li, 2020; Yi and Zheng, 2020; Zhang H. et al., 2021; Chen, 2021; Zhou et al., 2021; Liang et al., 2022; Liu, 2022; Lyu et al., 2022; Wang and Xu, 2022; Yang et al., 2022; Zhou et al., 2022), 1 comparing HM and placebo HM (Lyu et al., 2022), and the rest comparing HM plus conventional medication and conventional medication alone (Supplementary Material S3).

In the included studies, various HMs were used, and among them, Zhisou-san or its modified form was used the most frequently (13 studies) (Liu and Zhang, 2012; Chen, 2016; Cui et al., 2016; Li, 2018; Zhou, 2018; Gu, 2020; Lai, 2020; Niu et al., 2020; Huang, 2021; Guan, 2022; Song, 2022; Wang and Xu, 2022; Zhou et al., 2022). A total of 154 botanical drugs were used, and as a result of analyzing the constituent botanical drugs of basic HM, four studies were excluded that used different prescriptions according to the pattern identification (Chen et al., 2009; Yu et al., 2014; Hui and Dong, 2020; Tang et al., 2020). Among them, *Glycyrrhiza glabra* L. [Fabaceae; Glycyrrhizae Radix et Rhizoma] was used the most (61 studies), followed by *Platycodon grandiflorus* (Jacq.) A.DC. [Campanulaceae; Platycodonis Radix] (41 studies), *Aster tataricus* L.f. [Asteraceae; Asteris Radix et Rhizoma] (36 studies), *Pinellia ternata* (Thunb.) Makino [Araceae; Pinelliae Tuber] (29 studies), *Citrus × aurantium* f. deliciosa (Ten.) M. Hiroe [Rutaceae; Citri Unshius Pericarpium] (28 studies), *Prunus armeniaca* L. [Rosaceae; Armeniacae Semen] (27 studies), *Poria cocos* Wolf [Polyporaceae; Poria Sclerotium] (24 studies), *Stemona tuberosa* Lour. [Stemonaceae; Stemonae Radix] (22 studies), and *Schisandra chinensis* (Turcz.) Baill. [Schisandraceae; Schisandrae Fructus] (20 studies). As for the dosage of HMs, decoction was the most common (68 studies), followed by granule (5 studies) and oral liquid (3 studies). The HM administration duration varied from 1 week to 3 months, with 2 weeks being the most (34 studies), followed by 4 weeks (13 studies), 1 month (9 studies), and 1 week (7 studies). Twelve studies performed follow-ups after the end of the HM administration, and the follow-up period varied from 1 week to 1 year (Supplementary Material S4).

### 3.2 Risk of bias assessment

All the studies appropriately generated random sequences using random number tables or dice-throwing methods, and none of the studies mentioned allocation concealment. Except for one study comparing HM and placebo HM (Lyu et al., 2022), no study conducted blinding of the participants and personnel. In addition, only two studies blinded the outcome assessors (Lu et al., 2013; Lyu et al., 2022). Four studies were evaluated as having a high risk of attrition bias by a per-protocol analysis (Chen et al., 2009; Shi et al., 2010; Liang et al., 2022; Zhao and Tuo, 2022). All the studies were rated as having a low risk of reporting bias and other biases (Supplementary Material S5).

### 3.3 HM *versus* conventional medication

1) Primary outcome: cough severity

Although there was no significant difference in the SCS total score between the two groups (MD -0.07, 95% CI −0.31 to 0.18, I^2^ = 0%), each daytime (MD −0.34, 95% CI −0.59 to −0.08, I^2^ = 91%) or nighttime SCS score (MD −0.38, 95% CI -0.58 to −0.19, I^2^ = 86%) significantly decreased in the HM group compared with the conventional medication group (Table 1).

2) Secondary outcomes

**TABLE 1:** Summary of findings.

Outcomes	No. participants	No. RCTs	Effect estimate [95% CI]*		I^2^ value (%)	Certainty of evidence	Reasons for downgrading
HM *versus* conventional medication							
** **SCS (total)	142	2 Tang et al. (2020); Yi and Zheng (2020)	MD	−0.07 [**−**0.31, 0.18]	0	Low	Risk of bias, Imprecision
** **SCS (daytime)	442	6 Hui and Dong (2020); Meng et al. (2020); Tang et al. (2020); Wu and Li (2020); Yi and Zheng (2020); Wang and Xu (2022)	MD	**−0.34 [-0.59, −0.08]**	91	Low	Risk of bias, Inconsistency
** **SCS (nighttime)	442	6 Hui and Dong (2020); Meng et al. (2020); Tang et al. (2020); Wu and Li (2020); Yi and Zheng (2020); Wang and Xu (2022)	MD	**−0.38 [-0.58, −0.19]**	86	Moderate	Risk of bias
** **LCQ (total)	224	3 Hui and Dong (2020); Meng et al. (2020); Tang et al. (2020)	MD	**2.32 [0.04, 4.61]**	96	Moderate	Risk of bias
** **LCQ (physical items)	322	4 Zhu (2017); Hui and Dong (2020); Meng et al. (2020); Tang et al. (2020)	MD	**0.63 [0.30, 0.95]**	75	Moderate	Risk of bias
** **LCQ (psychological items)	322	4 Zhu (2017); Hui and Dong (2020); Meng et al. (2020); Tang et al. (2020)	MD	**0.58 [0.30, 0.87]**	71	Moderate	Risk of bias
** **LCQ (social items)	322	4 Zhu (2017); Hui and Dong (2020); Meng et al. (2020); Tang et al. (2020)	MD	**0.66 [0.14, 1.18]**	88	Moderate	Risk of bias
** **TER	2,994	33 Han et al. (2008); Shi et al. (2010); Deng (2012); Liu and Zhang (2012); Wang (2012); Xie et al. (2012); Yang and Yang (2012); Zhu (2012); Lu et al. (2013); Yu et al. (2014); Zhang et al. (2016); Fan (2017); Xie (2017); Zhu (2017); Li (2018); Wang and Li (2019); Gu (2020); Hui and Dong (2020); Lai (2020); Li (2020); Liu (2020); Meng et al. (2020); Tang et al. (2020); Wu and Li (2020); Yi and Zheng (2020); Zhang et al. (2021b); Chen (2021); Zhou et al. (2021); Liang et al. (2022); Liu (2022); Wang and Xu (2022); Yang et al. (2022); Zhou et al. (2022)	RR	**1.23 [1.18, 1.29]**	35	Low	Risk of bias, Publication bias
age: children	394	5 Liu and Zhang (2012); Yu et al. (2014); Li (2018); Liang et al. (2022); Yang et al. (2022)	RR	**1.23 [1.12, 1.34]**	0	Moderate	Risk of bias
age: adults	2,272	25 Han et al. (2008); Shi et al. (2010); Deng (2012); Wang (2012); Xie et al. (2012); Yang and Yang (2012); Zhu (2012); Lu et al. (2013); Xie (2017); Zhu (2017); Wang and Li (2019); Gu (2020); Hui and Dong (2020); Lai (2020); Li (2020); Liu (2020); Meng et al. (2020); Tang et al. (2020); Yi and Zheng (2020); Zhang et al. (2021b); Chen (2021); Zhou et al. (2021); Liu (2022); Wang and Xu (2022); Zhou et al. (2022)	RR	**1.24 [1.18, 1.31]**	48	Low	Risk of bias, Publication bias
age: both	328	3 Zhang et al. (2016); Fan (2017); Wu and Li (2020)	RR	**1.24 [1.12, 1.36]**	0	Moderate	Risk of bias
** **Cough recurrence rate	222	3 Li (2020); Zhang et al. (2021b); Zhou et al. (2022)	RR	**0.29 [0.16, 0.51]**	0	Low	Risk of bias, Imprecision
** **Incidence of adverse events	1,503	18 Han et al. (2008); Shi et al. (2010); Lu et al. (2013); Zhang et al. (2016); Gu (2020); Hui and Dong (2020); Lai (2020); Liu (2020); Meng et al. (2020); Wu and Li (2020); Yi and Zheng (2020); Zhang et al. (2021b); Chen (2021); Zhou et al. (2021); Liang et al. (2022); Wang and Xu (2022); Yang et al. (2022); Zhou et al. (2022)	RR	**0.26 [0.17, 0.40]**	0	Low	Risk of bias, Imprecision
age: children	190	2 Liang et al. (2022); Yang et al. (2022)	RR	0.67 [0.12, 3.81]	NA	Low	Risk of bias, Imprecision
age: adults	1,181	14 Han et al. (2008); Shi et al. (2010); Lu et al. (2013); Gu (2020); Hui and Dong (2020); Lai (2020); Liu (2020); Meng et al. (2020); Yi and Zheng (2020); Zhang et al. (2021b); Chen (2021); Zhou et al. (2021); Wang and Xu (2022); Zhou et al. (2022)	RR	**0.24 [0.15, 0.40]**	0	Low	Risk of bias, Imprecision
age: both	132	2 Zhang et al. (2016); Wu and Li (2020)	RR	**0.25 [0.08, 0.81]**	NA	Low	Risk of bias, Imprecision
HM plus conventional medication *versus* conventional medication alone							
** **SCS (daytime)	979	9 Shen et al. (2017); Yan et al. (2018); Tan and Zheng (2020); Wang (2020); Yang et al. (2020); He (2021); Li et al. (2021); Ji (2022); Zhang and Wang (2022)	MD	**−0.53 [−0.67, −0.39]**	97	Moderate	Risk of bias
age: children	453	4 Yan et al. (2018); Wang (2020); He (2021); Li et al. (2021)	MD	**−0.48 [−0.67, −0.28]**	98	Moderate	Risk of bias
age: adults	526	5 Shen et al. (2017); Tan and Zheng (2020); Yang et al. (2020); Ji (2022); Zhang and Wang (2022)	MD	**−0.58 [-0.77, −0.39]**	89	Moderate	Risk of bias
cause of cough: mixed	283	3 Wang (2020); Yang et al. (2020); Li et al. (2021)	MD	**−0.47 [−0.69, −0.25]**	90	Moderate	Risk of bias
cause of cough: NR	696	6 Shen et al. (2017); Yan et al. (2018); Tan and Zheng (2020); He (2021); Ji (2022); Zhang and Wang (2022)	MD	**−0.56 [−0.75, −0.37]**	96	Moderate	Risk of bias
** **SCS (nighttime)	979	9 Shen et al. (2017); Yan et al. (2018); Tan and Zheng (2020); Wang (2020); Yang et al. (2020); He (2021); Li et al. (2021); Ji (2022); Zhang and Wang (2022)	MD	**−0.55 [−0.69, −0.41]**	95	Moderate	Risk of bias
age: children	453	4 Yan et al. (2018); Wang (2020); He (2021); Li et al. (2021)	MD	**−0.54 [-0.83, -0.25]**	96	Moderate	Risk of bias
age: adults	526	5 Shen et al. (2017); Tan and Zheng (2020); Yang et al. (2020); Ji (2022); Zhang and Wang (2022)	MD	**−0.56 [-0.67, -0.44]**	81	Moderate	Risk of bias
cause of cough: mixed	283	3 Wang (2020); Yang et al. (2020); Li et al. (2021)	MD	**−0.44 [-0.63, -0.25]**	85	Moderate	Risk of bias
cause of cough: NR	696	6 Shen et al. (2017); Yan et al. (2018); Tan and Zheng (2020); He (2021); Ji (2022); Zhang and Wang (2022)	MD	**−0.62 [-0.71, -0.53]**	71	Moderate	Risk of bias
0–100 mm Cough VAS	202	3 Gao et al. (2017); Yang et al. (2020); Tian (2023)	MD	**−11.69 [-21.71, -1.67]**	98	Moderate	Risk of bias
** **LCQ (physical items)	356	4 Ge et al. (2017); Yan et al. (2018); Chen et al. (2019); Yang et al. (2019)	MD	**0.88 [0.71, 1.05]**	0	Moderate	Risk of bias
age: children	176	2 Ge et al. (2017); Yan et al. (2018)	MD	**0.82 [0.55, 1.08]**	1	Moderate	Risk of bias
age: adults	180	2 Chen et al. (2019); Yang et al. (2019)	MD	**0.93 [0.70, 1.16]**	0	Moderate	Risk of bias
** **LCQ (psychological items)	356	4 Ge et al. (2017); Yan et al. (2018); Chen et al. (2019); Yang et al. (2019)	MD	**0.62 [0.46, 0.77]**	0	Moderate	Risk of bias
age: children	176	2 Ge et al. (2017); Yan et al. (2018)	MD	**0.54 [0.33, 0.76]**	11	Moderate	Risk of bias
age: adults	180	2 Chen et al. (2019); Yang et al. (2019)	MD	**0.72 [0.48, 0.95]**	0	Moderate	Risk of bias
** **LCQ (social items)	356	4 Ge et al. (2017); Yan et al. (2018); Chen et al. (2019); Yang et al. (2019)	MD	**0.81 [0.44, 1.17]**	71	Moderate	Risk of bias
age: children	176	2 Ge et al. (2017); Yan et al. (2018)	MD	**0.53 [0.19, 0.86]**	34	Moderate	Risk of bias
age: adults	180	2 Chen et al. (2019); Yang et al. (2019)	MD	**1.08 [0.79, 1.37]**	0	Moderate	Risk of bias
** **TER	4,525	46 Chen et al. (2009); Zhou (2011); Gan and Zhu (2013); Chen (2014); Cui (2014); Lin (2014); Lu and Pan (2014); Chen (2016); Cui et al. (2016); Wang and Zang (2016); Wang and Zhou (2016); Zhang et al. (2016); Gao et al. (2017); Ge et al. (2017); Shen et al. (2017); Xu (2017); Zhou et al. (2017); Yan et al. (2018); Zhou (2018); Chen et al. (2019); Li (2019); Shen and Huang (2019); Wu et al. (2019); Yang et al. (2019); Niu et al. (2020); Sun and Wei (2020); Wang (2020); Yang et al. (2020); Zhang and Chen (2020); Chen and Chen (2021); Diao (2021); He (2021); Huang (2021); Li et al. (2021); Luan et al. (2021); Xia (2021); Zhao et al. (2022a); Zhao et al. (2022b); Gong (2022); Guan (2022); Ji (2022); Li et al. (2022); Song (2022); Zhang and Wang (2022); Zhao and Tuo (2022); Tian (2023)	RR	**1.18 [1.16, 1.21]**	0	Low	Risk of bias, Publication bias
** **Cough recurrence rate	486	6 Chen et al. (2009); Cui et al. (2016); Zhao et al. (2022a); Zhao et al. (2022b); Guan (2022); Li et al. (2022)	RR	**0.30 [0.19, 0.46]**	0	Low	Risk of bias, Imprecision
age: children	330	4 Chen et al. (2009); Cui et al. (2016); Zhao et al. (2022a); Li et al. (2022)	RR	**0.32 [0.19, 0.52]**	0	Low	Risk of bias, Imprecision
age: adults	156	2 Zhao et al. (2022b); Guan (2022)	RR	**0.22 [0.08, 0.63]**	0	Low	Risk of bias, Imprecision
** **Incidence of adverse events	1,489	17 Chen (2014); Cui (2014); Zhang et al. (2016); Ge et al. (2017); Zhou (2018); Li (2019); Shen and Huang (2019); Wu et al. (2019); Tan and Zheng (2020); Wang (2020); Chen and Chen (2021); Li et al. (2021); Luan et al. (2021); Guan (2022); Li et al. (2022); Song (2022); Zhang and Wang (2022)	RR	**0.46 [0.25, 0.87]**	50	Very low	Risk of bias, Inconsistency, Imprecision

CI, confidence interval; HM, herbal medicine; LCQ, leicester cough questionnaire; MD, mean difference; NA, not applicable; NR, not reported; RCT, randomized controlled clinical trial; RR, risk ratio; SCS, simplified cough score; TER, total effective rate; VAS, visual analog scale.

*Bold value indicates a significant difference between two groups.

Cough-related quality of life, assessed by the LCQ total score, and physical, psychological, and social sub-items, were significantly increased after the treatment in the HM group compared with the conventional medication group (total: MD 2.32, 95% CI 0.04 to 4.61, I^2^ = 96%; physical items: MD 0.63, 95% CI 0.30 to 0.95, I^2^ = 75%; psychological items: MD 0.58, 95% CI 0.30 to 0.87, I^2^ = 71%; social items: MD 0.66, 95% CI 0.14 to 1.18, I^2^ = 88%). In addition, the TER, calculated according to the cough symptom improvement, was significantly higher in the HM group compared with the conventional medication group (RR 1.23, 95% CI 1.18 to 1.29, I^2^ = 35%), regardless of subgroup according to age. The funnel plot for the TER was asymmetric, and the result of the Egger’s test also suggested the risk of publication bias with a *p*-value of less than 0.05 (Supplementary Material S6). Cough recurrence rate was significantly lower in the HM group (RR 0.29, 95% CI 0.16 to 0.51, I^2^ = 0%) (Table 1).

Eighteen studies comparing HM and conventional medication reported the incidence of adverse events (Han et al., 2008; Shi et al., 2010; Lu et al., 2013; Zhang et al., 2016; Gu, 2020; Hui and Dong, 2020; Lai, 2020; Liu, 2020; Meng et al., 2020; Wu and Li, 2020; Yi and Zheng, 2020; Zhang H. et al., 2021; Chen, 2021; Zhou et al., 2021; Liang et al., 2022; Wang and Xu, 2022; Yang et al., 2022; Zhou et al., 2022). The incidence of adverse events was significantly lower in the HM group compared with the conventional medication group (RR 0.26, 95% CI 0.17 to 0.40, I^2^ = 0%). The funnel plot was symmetric, and the *p*-value of the Egger’s test was 0.917, suggesting that the risk of publication was low (Supplementary Material S6). When analyzing subgroups according to age, insignificant results were shown between the two groups in children, but adverse events were significantly lower in the HM group in adults and mixed populations (Table 1).

### 3.4 HM plus conventional medication *versus* conventional medication alone

1) Primary outcome: cough severity

Cough severity, evaluated by SCS daytime and nighttime scores, was significantly lower in the HM plus conventional medication group (SCS daytime: MD −0.53, 95% CI −0.67 to −0.39, I^2^ = 97%; SCS nighttime: MD −0.55, 95% CI −0.69 to −0.41, I^2^ = 95%), which was consistent with subgroup analyses according to age and cause of cough. In addition, cough severity, assessed by the 0–100 mm cough VAS, also significantly improved in the HM plus conventional medication group (MD −11.69, 95% CI −21.71 to −1.67, I^2^ = 98%) (Table 1).

2) Secondary outcomes

Cough-related quality of life, assessed by the LCQ, was significantly improved in the HM plus conventional medication group compared with conventional medication alone (physical items: MD 0.88, 95% CI 0.71 to 1.05, I^2^ = 0%; psychological items: MD 0.62, 95% CI 0.46 to 0.77, I^2^ = 0%; social items: MD 0.81, 95% CI 0.44 to 1.17, I^2^ = 71%), and these results were consistent in the subgroup analysis according to age. One study targeted adults with chronic cough due to GERD (Gao et al., 2017). When HM was additionally administered with conventional medication (rabeprazole and mosapride citrate) for 8 weeks, cough severity and quality of life, assessed by the chronic cough impact scale, were significantly improved compared to conventional medication alone (*p* < 0.01). One study targeted pediatric patients with chronic cough due to UACS (Luan et al., 2021). When HM was additionally administered with conventional medication (budesonide nasal spray) for 12 weeks, cough frequency per day was significantly improved compared to conventional medication alone (*p* < 0.05).

The TER, according to the cough symptom improvement, was significantly higher in the HM plus conventional medication group (RR 1.18, 95% CI 1.16 to 1.21, I^2^ = 0%). The funnel plot for the TER was asymmetric, and the result of Egger’s test also suggested the risk of publication bias with a *p*-value of less than 0.05 (Supplementary Material S6). The cough symptom recurrent rate was significantly lower in the HM plus conventional medication group (RR 0.30, 95% CI 0.19 to 0.46, I^2^ = 0%), and the statistical significance was consistent when conducting a subgroup analysis according to age (Table 1).

Seventeen studies comparing HM plus conventional medication and conventional medication alone reported the incidence of adverse events (Chen, 2014; Cui, 2014; Zhang et al., 2016; Ge et al., 2017; Zhou, 2018; Li, 2019; Shen and Huang, 2019; Wu et al., 2019; Tan and Zheng, 2020; Wang, 2020; Chen and Chen, 2021; Li et al., 2021; Luan et al., 2021; Guan, 2022; Li et al., 2022; Song, 2022; Zhang and Wang, 2022), and it was found that adverse events were significantly lower in the HM plus conventional medication group (RR 0.46, 95% CI 0.25 to 0.87, I^2^ = 50%) (Table 1). The funnel plot was symmetric, and the *p*-value of Egger’s test was 0.433, suggesting the risk of publication was low (Supplementary Material S6).

### 3.5 HM *versus* placebo HM

Only one study compared HM *versus* placebo HM (Lyu et al., 2022), and therefore, a meta-analysis was not possible. In the study, HM significantly improved cough severity, measured by the cough diary score and cough VAS, and cough-related quality of life, measured by LCQ, compared with the baseline. HM significantly improved daytime and total cough diary score compared to the placebo HM.

### 3.6 Certainty assessment

The certainty of the evidence for all meta-analysis outcomes comparing HM to conventional medication was moderate to low. The main reason for downgrading was the risk of bias of the included studies, inconsistency, imprecision, and publication bias. The certainty of the evidence for cough severity and cough-related quality of life when comparing HM plus conventional medication and conventional medication alone was moderate, downgraded by the risk of bias of the included studies. In this comparison group, the certainty of the evidence for TER and cough recurrence rate was low, and the certainty of the evidence for adverse event rates was very low (Table 1).

## 4 Discussion

To summarize the effect of HM on chronic cough, 11 databases were comprehensively searched, and a total of 80 relevant articles were included in this meta-analysis. Previous systematic reviews of the effect of HM on chronic cough were conducted on specific causes of chronic cough, such as adult CVA (Chen et al., 2021) or UACS patients (Jiang et al., 2016). A study also synthesized relevant studies on HM up to 2012 without distinction of acute or chronic cough (Wagner et al., 2015). Our study differs from these previous studies in that it updated the effect of HM on chronic cough regardless of the cause of the cough or age.

According to our findings, when HM was used as a monotherapy, there were inconsistent results across rating scales for cough severity. However, compared to conventional medication, HM significantly improved cough-related quality of life and TER and significantly lowered the cough recurrence rate. The certainty of the evidence was mainly low. When HM was used as an add-on therapy with conventional medication, the combination of therapies significantly improved cough severity, cough-related quality of life, and TER and significantly lowered the cough recurrence rate compared to conventional medication alone. The certainty of the evidence was mainly moderate. A subgroup analysis according to age and cause of cough also showed a statistically consistent direction with the overall results. However, only a small number of studies reported the cause of cough. In addition, the HM group had a significantly lower incidence of adverse events than the conventional medication group when HM was used as an add-on or monotherapy.

Cough frequency, one of our secondary outcomes, was evaluated as the number of coughs per day in only one study (Luan et al., 2021), and how the number of coughs was counted was not reported; however, cough frequency significantly improved in the HM group. A cough frequency meter is an objective standard evaluation method of coughing, and a representative example is the Leicester cough monitor (Morice et al., 2007). The correlation between cough frequency using an objective cough frequency meter and subjective cough-related quality of life LCQ scores was reported to be moderate (Birring et al., 2006), suggesting that there is a difference between perceived and actual cough frequency. Therefore, in future clinical trials to evaluate the effect of HM on coughing, subjective questionnaires, and tools to evaluate cough frequency objectively should be used to confirm the effect of HM on actual cough frequency.

Chronic cough has a variety of causes, and more than one disease can cause a complex effect inducing cough. In many cases, there is no specific symptom besides coughing or a definitive test method (Morice et al., 2020). Therefore, many nonspecific chronic coughs are difficult to treat by identifying the cause of the disease in the clinical setting, so empirical treatment is often performed first (Morice et al., 2020). In addition, it has been reported that 10%–40% of patients with chronic cough who visit secondary or tertiary medical institutions have an unexplained cough that does not resolve well despite the best diagnostic tests and treatment efforts (McGarvey, 2013). Accordingly, there is a need for an effective treatment approach for chronic cough that exhibits complex pathophysiology rather than simply a symptom of another disease. We attempted a subgroup analysis of the effect of HM according to the cause of the cough; however, 50 studies (62.5% of the included studies) did not report the cause of the cough. In addition, there was only one study on nonspecific chronic cough and three on unexplained chronic cough. Due to its multi-component and multi-target characteristics, HM has the potential to act effectively on chronic cough from various causes or nonspecific or unexplained chronic coughs. In particular, the effect of Maekmundong-tang (Maimendong-tang in Chinese), one of the HM, on cough hypersensitivity accompanied by chronic cough has been reported through clinical studies (Watanabe et al., 2003; Watanabe et al., 2004). An RCT is also being attempted to explore the effects of Maekmundong-tang on nonspecific chronic cough (Lee et al., 2023). Therefore, in clinical studies evaluating the effect of HM on chronic cough in the future, the cause of the cough should be reported if known, or it should be specified as an explained or nonspecific chronic cough based on the definition in the guideline (Gibson et al., 2016; Song et al., 2018). This specification will also help to explore the therapeutic mechanism of HM for the treatment of cough.

The therapeutic mechanism of HM on chronic cough has been proposed in various ways. Among the included studies, Zhisou-san, or its modified form, was used most frequently, which was reported to be related to inflammation and Th17/Treg immune balance regulation for the treatment of CVA (Guo et al., 2022). In addition, the pulmonary protective effect and antitussive effect of *P. grandiflorus* (Jacq.) A.DC. [Campanulaceae; Platycodonis Radix] and liquiritin, a flavonoid extracted from *G. glabra* L. [Fabaceae; Glycyrrhizae Radix et Rhizoma], which were used frequently in the included studies, have been reported (Zhang C. et al., 2021; Qin et al., 2022). The antitussive, anti-inflammatory, and expectorant effects of *A. tataricus* L.f. [Asteraceae; Asteris Radix et Rhizoma] and *P. ternata* (Thunb.) Makino [Araceae; Pinelliae Tuber] were also reported (Ok et al., 2009; Yu et al., 2015). In addition to the protective effect on the respiratory system, some HMs, including Maekmundong-tang, have been reported to have a therapeutic effect on GERD-induced cough through mechanisms such as relieving airway reflux and hypersensitivity (Lyu et al., 2022). These mechanisms could potentially contribute to the relief of chronic cough caused by GERD. As such, it seems that HM may treat cough due to the synergistic effect of these various botanical drugs, and additional research is needed to derive a clear mechanism.

This study has the following limitations. First, despite attempts at a subgroup analysis according to age and cause of cough, statistical heterogeneity could not be resolved. This difficulty may have been due to clinical heterogeneity, as the included studies used very different types of HM, and the duration of HM administration also varied widely. Second, the risk of bias for the included studies was not optimal, with a particularly high risk of performance and detection bias. There was only one study that evaluated the efficacy of HM compared with placebo HM (Lyu et al., 2022). Therefore, a planned sensitivity analysis excluding studies of low methodological quality was not possible. This also affected the certainty of the evidence, and the certainty of the evidence for the effect of HM was moderate to low.

Nevertheless, this study is the first to comprehensively synthesize the effects of HM regardless of age or cause of cough through a systematic review procedure. In addition, we assessed the risk of publication bias with the symmetry of the funnel plots and Egger’s tests and evaluated the certainty of the evidence with the GRADE method. In the future, clinical trials evaluating the effect of HM should be actively conducted in other countries, such as Korea and Japan, in addition to China, to help generalize and evaluate the effects of HM. In addition, the efficacy of HM compared to placebo HM should be evaluated using a validated and objective assessment tool.

## 5 Conclusion

HM as a monotherapy may improve cough severity and cough-related quality of life in patients with chronic cough. In addition, when used as an add-on to conventional medication, HM can significantly improve cough severity and cough-related quality of life. HM as a monotherapy or add-on therapy may lower the cough recurrence rate and incidence of adverse events. However, the certainty of the evidence for the effectiveness of HM was moderate to low due to the risk of bias in the included studies.

## Data Availability

The original contributions presented in the study are included in the article/Supplementary Material, further inquiries can be directed to the corresponding author.
