# Supplementary material for: Herbal medicine for the treatment of chronic cough: a systematic review and meta-analysis
Source: Front Pharmacol. 2023 Oct 17;14:1230604. doi: 10.3389/fphar.2023.1230604 (PMC10619915; doi:10.3389/fphar.2023.1230604)
Supplement: Supplementary file 1 [file DataSheet1.docx]

**Supplement 1. Search terms used in each database and results**

**Medline via PubMed**

|  | Searches | Results |
| --- | --- | --- |
| #1 | chronic[TIAB] AND cough[TIAB] | 11041 |
| #2 | “Plants, Medicinal”[MH] OR “Drugs, Chinese Herbal”[MH] OR “Medicine, Chinese Traditional”[MH] OR “Medicine, Kampo”[MH] OR “Medicine, Korean Traditional”[MH] OR “Herbal Medicine”[MH] OR “traditional Korean medicine”[TIAB] OR “traditional Chinese medicine”[TIAB] OR “traditional oriental medicine”[TIAB] OR “Kampo medicine”[TIAB] OR “medicinal plants”[TIAB] OR herb*[TIAB] OR prescription[TIAB] OR decoction[TIAB] OR tang[TIAB] OR capsule[TIAB] OR powder[TIAB] OR botanic*[TIAB] | 498416 |
| #3 | “randomized controlled trial”[PT] OR “controlled clinical trial”[PT] OR randomized[TIAB] OR placebo[TIAB] OR “drug therapy”[SH] OR randomly[TIAB] OR trial[TIAB] OR groups[TIAB] | 5682922 |
| #4 | animals[MH] NOT humans[MH] | 5101179 |
| #5 | #1 AND #2 AND #3 NOT #4 | **175** |

**EMBASE via Elsevier**

|  | Searches | Results |
| --- | --- | --- |
| #1 | ‘chronic cough’/exp OR (chronic:ab,ti AND cough:ab,ti) | 22830 |
| #2 | ‘medicinal plant’/exp OR ‘medicinal plant’:ab,ti OR ‘herbaceous agent’/exp OR ‘herbaceous agent’:ab,ti OR ‘chinese medicine’/exp OR ‘chinese medicine’:ab,ti OR ‘kampo medicine’/exp OR ‘kampo medicine’:ab,ti OR ‘kampo medicine (drug)’/exp OR ‘kampo medicine (drug)’:ab,ti OR ‘korean medicine’/exp OR ‘korean medicine’:ab,ti OR ‘herbal medicine’/exp OR ‘herbal medicine’:ab,ti OR ‘oriental medicine’/exp OR ‘oriental medicine’:ab,ti OR herb/exp OR herb*:ab,ti OR prescription:ab,ti OR decoction:ab,ti OR tang:ab,ti OR capsule:ab,ti OR powder:ab,ti OR botanic*:ab,ti | 849771 |
| #3 | 'crossover procedure':de OR 'double-blind procedure':de OR 'randomized controlled trial':de OR 'single-blind procedure':de OR (random* OR factorial* OR crossover* OR cross NEXT/1 over* OR placebo* OR doubl* NEAR/1 blind* OR singl* NEAR/1 blind* OR assign* OR allocat* OR volunteer*):de,ab,ti | 3097616 |
| #4 | #1 AND #2 AND #3 | **173** |

**CENTRAL**

|  | Searches | Results |
| --- | --- | --- |
| #1 | (chronic AND cough):ti,ab,kw | 2542 |
| #2 | MeSH descriptor: [Plants, Medicinal] explode all trees | 1040 |
| #3 | MeSH descriptor: [Drugs, Chinese Herbal] explode all trees | 4175 |
| #4 | MeSH descriptor: [Medicine, Chinese Traditional] explode all trees | 1537 |
| #5 | MeSH descriptor: [Medicine, Kampo] explode all trees | 58 |
| #6 | MeSH descriptor: [Medicine, Korean Traditional] explode all trees | 41 |
| #7 | MeSH descriptor: [Herbal Medicine] explode all trees | 85 |
| #8 | (“traditional Korean medicine” OR “traditional Chinese medicine” OR “traditional oriental medicine” OR “Kampo medicine” OR “medicinal plants” OR herb* OR prescription OR decoction OR tang OR capsule OR powder OR botanic*):ti,ab,kw | 74008 |
| #9 | #2 OR #3 OR #4 OR #5 OR #6 OR #7 OR #8 | 75228 |
| #10 | (#1 AND #9) in Trials | **289** |

**AMED via EBSCO**

|  | Searches | Results |
| --- | --- | --- |
| #1 | TX “chronic cough” OR (TX chronic AND TX cough) | 133 |
| #2 | SU “Herbal Medicine” OR SU “Herbal Drugs” OR SU herbs OR SU “Herbal Preparations” OR SU herbalism OR SU “Traditional Medicine Korean” OR SU “Traditional Medicine Chinese” OR SU “Kampo Medicine” OR SU Kampo OR SU “Medicine Kampo” OR SU “Traditional Medicine Oriental” OR TX “herbal medicine” OR TX “medicinal plants” OR TX herb* OR TX “traditional Chinese medicine” OR TX “traditional Korean medicine” OR TX Kampo OR TX “Oriental medicine*” OR TX prescription OR TX decoction OR TX tang OR TX capsule OR TX powder OR TX botanic* | 31492 |
| #3 | #1 AND #2 | **41** |

**OASIS**

|  | Searches | Results |
| --- | --- | --- |
| #1 | (만성기침) (한약\|약초\|본초\|탕\|환\|산) | **1** |

**KMbase**

|  | Searches | Results |
| --- | --- | --- |
| #1 | [ALL=만성기침] | 65 |
| #2 | ((((([ALL=한약] OR [ALL=약초]) OR [ALL=본초]) OR [ALL=탕]) OR [ALL=환]) OR [ALL=산]) | 117859 |
| #3 | #1 AND #2 | **42** |

**ScienceON**

|  | Searches | Results |
| --- | --- | --- |
| #1 | (만성기침) (한약\|약초\|본초\|탕\|환\|산) | **33** |

**RISS**

|  | Searches | Results |
| --- | --- | --- |
| #1 | (만성기침) (한약\|약초\|본초\|탕\|환\|산) | **27** |

**CNKI**

|  | Searches | Results |
| --- | --- | --- |
| #1 | (SU=‘慢性咳嗽’+’长期咳嗽’) AND (SU=‘中医药’+’中医’+’中西医结合’+’中药’+’本草’+’汤’+’丸’+’散’+’饮’+’颗粒’+’胶囊’) AND (’随机’+’对照’+’随意’+’试验’+’安慰’) | **770** |

**Wanfang data**

|  | Searches | Results |
| --- | --- | --- |
| #1 | (主题:慢性咳嗽 OR 主题:长期咳嗽) AND (主题:中医药 OR 主题:中医 OR 主题:中西医结合 OR 主题:中药 OR 主题:本草 OR 主题:汤 OR 主题:丸 OR 主题:散 OR 主题:饮 OR 主题:颗粒 OR 主题:胶囊) AND (主题:随机 OR 主题:对照 OR 主题:随意 OR 主题:试验 OR 主题:安慰) | **1406** |

**CiNii**

|  | Searches | Results |
| --- | --- | --- |
| #1 | (慢性咳嗽 OR 慢性咳 OR 慢性的な咳) AND (漢方薬 OR ハーブ OR 中药 OR 本草 OR 散 OR 汤 OR 丸) AND (ランダム化比較試験 OR 対照臨床試験 OR ランダム OR 無作為 OR 対照 OR 試験 OR 偽薬) | **1** |

**Supplement 2. Excluded studies after full text review**

1) not RCT (n = 444)

1. 柯旭, *小儿慢性咳嗽的中西医结合治疗方案.* 医学理论与实践, 2015. **28**(07): p. 892-893.

2. 康芳, *三拗汤合六君子汤治疗小儿慢性咳嗽的效果分析.* 中国冶金工业医学杂志, 2020. **37**(03): p. 266-267.

3. 耿立梅 and 郭媛媛, *郭纪生经验方肺系2号治疗慢性咳嗽的临床研究.* 现代中西医结合杂志, 2017. **26**(31): p. 3485-3486,3505.

4. 耿晓婷, *中医辨证治疗慢性咳嗽疗效观察.* 健康必读, 2020(3): p. 187.

5. 季锡林, *止嗽散加减治疗慢性咳嗽的临床效果.* 临床合理用药杂志, 2021. **14**(23): p. 67-69.

6. 高作良 and 高梅, *止嗽散合三拗汤治疗慢性咳嗽患儿的疗效及对炎症因子、免疫功能的影响.* 中医临床研究, 2019. **11**(36): p. 31-33.

7. 曲艳, et al., *槐杞黄颗粒治疗儿童呼吸道感染后慢性咳嗽临床疗效的研究.* 中国血液流变学杂志, 2016. **26**(2): p. 220-223.

8. 孔庆梅, *养阴清肺法治疗肺阴虚型慢性咳嗽疗效观察.* 健康女性, 2021(26): p. 67.

9. 孔维斌, *芪冬润肺方治疗气阴亏虚慢性咳嗽的有效性分析.* 医学理论与实践, 2016. **29**(18): p. 3199-3200.

10. 高晓琴, *经方止嗽散加味治疗300例慢性咳嗽的临床疗效.* 当代医药论丛, 2014. **12**(01): p. 176-177.

11. 郭建明, *浅议中药治疗慢性咳嗽.* 内蒙古中医药, 2015. **34**(06): p. 17-18.

12. 郭景瑞, *沙参麦冬汤治疗小儿慢性咳嗽肺阴亏虚证的临床效果.* 临床医学研究与实践, 2019. **4**(06): p. 97-98+122.

13. 郭洪刚, *中西医结合治疗慢性咳嗽60例分析.* 河北联合大学学报(医学版), 2012. **14**(06): p. 868.

14. 郭怀干, *止嗽散加减中药汤剂治疗慢性咳嗽的临床疗效观察.* 世界最新医学信息文摘（连续型电子期刊）, 2020. **20**(51): p. 195,197.

15. 郭龙, *半夏泻心汤加减治疗慢性咳嗽的临床疗效研究.* 中国卫生标准管理, 2018. **9**(24): p. 80-82.

16. 匡奕亮, et al., *苓甘五味姜辛汤合止嗽散加减治疗慢性咳嗽临床观察.* 中外医学研究, 2020. **18**(26): p. 28-30.

17. 邱宏, *使用参芪白术散合泻白散治疗小儿慢性咳嗽的效果分析.* 当代医药论丛, 2015. **13**(17): p. 25-26.

18. 仇年芳 and 童蓓丽, *滋阴活血法治疗阴虚血瘀型慢性咳嗽30例.* 中国中西医结合耳鼻咽喉科杂志, 2015. **23**(6): p. 466-467.

19. 丘梅清, *加味旋复代赭汤治疗慢性咳嗽30例.* 广州医药, 2011. **42**(05): p. 48-49.

20. 吉建中, *自拟清肺化痰止咳汤加减治疗80例慢性咳嗽的临床效果.* 中国处方药, 2017. **15**(8): p. 94-95.

21. 金光, *中西医结合治疗慢性咳嗽33例临床效果观察.* 中国保健营养, 2019. **29**(15): p. 331.

22. 金石园, *自拟清肺化痰止咳汤加减治疗慢性咳嗽的临床效果分析.* 中国社区医师, 2020. **36**(9): p. 96-97.

23. 金辉杰, *70例中医治疗小儿慢性咳嗽临床分析.* 医学信息, 2013(22): p. 562-563.

24. 南俊国, *分析小儿肺炎支原体感染后痰湿蕴肺型慢性咳嗽采用加味二陈汤治疗的效果.* 中国医药指南, 2019. **17**(28): p. 165-166.

25. 念小桃, *新升阳益胃汤治疗慢性咳嗽患者的临床研究.* 大家健康(学术版), 2015. **9**(08): p. 31.

26. 段印会, *止嗽散加减治疗慢性咳嗽的临床疗效分析.* 世界最新医学信息文摘, 2021. **21**(85): p. 515-516.

27. 段正英, *小儿鼻后流滴症所致慢性咳嗽的中西医结合治疗.* 中外健康文摘, 2013(22): p. 165-166.

28. 唐明杰, *苓甘五味姜辛汤联合止嗽散加减治疗慢性咳嗽40例临床观察.* 中医临床研究, 2021. **13**(18): p. 29-32.

29. 唐存祥 and 胡一莉, *利肺汤治疗慢性咳嗽40例疗效观察.* 浙江中医杂志, 2015. **50**(01): p. 31.

30. 唐洁芬 and 余培煌, *养阴清肺汤联合孟鲁司特治疗小儿支原体感染肺炎后慢性咳嗽临床观察.* 四川中医, 2016. **34**(08): p. 72-75.

31. 戴蓓, et al., *加味止嗽散治疗慢性咳嗽26例疗效观察.* 湖南中医杂志, 2015. **31**(07): p. 42-43.

32. 戴盈, *反流性食管炎引起慢性咳嗽的临床治疗效果分析.* 医药前沿, 2014(12): p. 173-173,174.

33. 代育中, *苏黄止咳胶囊联合布地奈德混悬液治疗儿童咳嗽变异性哮喘75例临床观察.* 河北中医, 2013(11): p. 1683-1684.

34. 戴兴龙 and 吴玄珠, *自拟止咳养肺汤治疗儿童慢性咳嗽40例.* 中国中医药科技, 2022. **29**(03): p. 506-508.

35. 代红 and 朱凤娟, *中西医结合治疗慢性咳嗽的临床效果观察.* 医药前沿, 2020. **10**(19): p. 79-80.

36. 涂媚, *苓甘五味姜辛汤合止嗽散加减治疗慢性咳嗽临床观察.* 实用中医药杂志, 2018. **34**(01): p. 15-16.

37. 童玉琴, *自拟理中止嗽散治疗慢性咳嗽80例——附西药治疗80例对照.* 浙江中医杂志, 2004(02): p. 19.

38. 董红娟, *麦门冬汤治疗慢性咳嗽临床体会.* 中医药临床杂志, 2016. **28**(09): p. 1248-1249.

39. 童鹏伟, *自拟活血止漱汤加减治疗慢性支气管炎50例.* 中医药临床杂志, 2005. **17**(5): p. 478.

40. 杜占申, *中西医结合治疗小儿慢性咳嗽92例临床观察及分析.* 中国现代药物应用, 2014. **8**(16): p. 162-163.

41. 杜定益, *苓甘五味姜辛汤合止嗽散加减治疗慢性咳嗽的临床价值体会.* 养生保健指南, 2019(35): p. 273.

42. 杜启鹏, *中西医结合治疗小儿咳嗽变异性哮喘疗效观察.* 海南医学, 2001. **12**(7): p. 25.

43. 梁雪 and 仇志锴, *三拗汤合六君子汤化裁治疗小儿慢性咳嗽的临床疗效.* 临床医药文献电子杂志, 2020. **7**(28): p. 31+41.

44. 雷颖 and 陈庆海, *止嗽散治疗小儿慢性咳嗽的临床观察.* 实用中西医结合临床, 2017. **17**(03): p. 128-129+131.

45. 廖福建 and 汪莎莎, *小柴胡汤联合香砂六君子汤治疗小儿慢性咳嗽疗效观察.* 临床合理用药杂志, 2019. **12**(10): p. 103-104.

46. 廖颖钊, 赵丽, and 王璋, *益肺祛邪止咳方治疗儿童慢性咳嗽60例疗效观察.* 河北中医, 2015. **37**(10): p. 1487-1488.

47. 李佳佳, *中西医结合治疗小儿慢性咳嗽疗效分析.* 保健文汇, 2021. **22**(15): p. 239-240.

48. 李杰, *中药方治疗小儿慢性咳嗽疗效观察.* 中国现代药物应用, 2010. **4**(19): p. 144-145.

49. 李冬梅, 周维维, and 曹骅, *祛风止咳汤结合西医治疗小儿慢性咳嗽30例疗效观察.* 中国优生优育, 2014. **20**(01): p. 25-27.

50. 李隆庆, *桔前汤治疗小儿慢性咳嗽30例疗效观察.* 河北中医, 2012. **34**(05): p. 680.

51. 李立政, *小儿支原体肺炎后慢性咳嗽应用养阴清肺汤效果探讨.* 健康必读, 2020(24): p. 167.

52. 李柏林, *慢性咳嗽中医治疗效果观察.* 医学信息, 2013(24): p. 469-470.

53. 李小慧, 张素卿, and 李巧兰, *沙参麦冬汤加减治疗老年慢性咳嗽体会.* 实用中医药杂志, 2013. **29**(04): p. 287.

54. 李松林, *自拟麻杏利咽汤治疗变异性哮喘慢性咳嗽的临床效果观察.* 临床合理用药杂志, 2017. **10**(32): p. 37-38.

55. 李秀英, *止嗽散加减治疗慢性咳嗽疗效观察.* 中国实用医药, 2010. **5**(08): p. 151-152.

56. 李新 and 王有鹏, *抗支口服液治疗小儿咳嗽变异性哮喘25例.* 中医药信息, 2005. **22**(6): p. 30-31.

57. 李雅莉, *宣降止咳汤治疗小儿慢性咳嗽的临床疗效观察.* 航空航天医学杂志, 2011. **22**(07): p. 892-893.

58. 李影捷 and 唐兴荣, *生姜五味子汤加减治疗久咳不愈56例.* 新中医, 2000. **32**(1): p. 33.

59. 李迎春, *苓甘五味姜辛汤合止咳散加减治疗慢性咳嗽的疗效及安全性分析.* 中国农村卫生, 2021. **13**(04): p. 5-6.

60. 李玉强, *经方合用治疗小儿咳嗽变异型哮喘29例.* 国医论坛, 2011. **26**(5): p. 9.

61. 李玉兰, 郭燕华, and 董晓莉, *黄芪五味辛甘汤治疗小儿上气道咳嗽综合征61例.* 中国中医药科技, 2012. **19**(3): p. 272-273.

62. 李勇, *中医辩证治疗慢性咳嗽的效果观察.* 东方药膳, 2019(19): p. 244.

63. 李育祥, *苓甘五味姜辛汤合止嗽散加减治疗慢性咳嗽的思路探讨.* 中国社区医师, 2014. **30**(05): p. 86-87.

64. 李寅, *中西医结合治疗小儿呼吸道感染长期咳嗽.* 齐齐哈尔医学院学报, 2003(07): p. 777.

65. 李俊霞, *中西医结合治疗儿童咳嗽变异性哮喘54例疗效观察.* 河北中医, 2006. **28**(11): p. 844-845.

66. 李春, *止嗽散加减中药汤剂用于慢性咳嗽患者的临床疗效.* 特别健康, 2021(19): p. 111-112.

67. 李忠林 and 李毅芳, *探究贝蒌止嗽散治疗小儿慢性咳嗽干咳类型的临床疗效.* 中国保健营养, 2017. **27**(30): p. 270.

68. 李平端, *二陈汤合三拗汤加减治疗咳嗽变异型哮喘50例.* 河北中医, 2010. **32**(4): p. 541-542.

69. 李海宁, *止咳散加味治疗外感后慢性咳嗽46例.* 实用中医内科杂志, 2006(02): p. 189.

70. 林柳廷, *小儿支原体肺炎后慢性咳嗽应用养阴清肺汤联合孟鲁司特治疗的效果.* 人人健康, 2020(08): p. 103.

71. 林丛, *自拟方平喘颗粒治疗小儿咳嗽变异性哮喘(寒性哮喘)的临床疗效观察.* 首都食品与医药, 2021. **28**(24): p. 143-144.

72. 林钰久, et al., *应用止嗽散合三拗汤加减治疗慢性咳嗽临床效果分析.* 自我保健, 2022(3): p. 268-269.

73. 林锡丰, *止咳散加味治疗外感后慢性咳嗽55例的临床疗效分析.* 中医临床研究, 2015. **7**(01): p. 104-105.

74. 李亚飞, *中西医结合治疗小儿过敏性咳嗽60例.* 浙江中医杂志, 2010. **45**(1): p. 57.

75. 李华 and 李颉, *中西医结合治疗小儿慢性咳嗽的临床研究.* 河北医学, 2015. **21**(12): p. 1965-1967.

76. 李华, 李颉, and 郭婷婷, *三拗汤合六君子汤化裁治疗小儿慢性咳嗽临床研究.* 河南中医, 2017. **37**(07): p. 1255-1257.

77. 李华, 李颉, and 李飞飞, *三拗汤合六君子汤治疗儿童慢性咳嗽(痰湿阻肺证)的疗效观察.* 上海医药, 2020. **41**(21): p. 4-6,57.

78. 李华成, *采用小柴胡汤加减治疗难治性慢性咳嗽的临床效果评析.* 当代医药论丛, 2018. **16**(07): p. 223-224.

79. 李国霞, *自拟止嗽汤治疗慢性咳嗽.* 医药前沿, 2012. **2**(2): p. 361.

80. 李杨, 谢钧, and 温伟波, *宣肺降气汤治疗慢性咳嗽临床观察.* 吉林中医药, 2008(08): p. 573.

81. 李红, *止嗽降气汤对慢性咳嗽的疗效观察.* 医药前沿, 2014(10): p. 374-375.

82. 李聪敏, et al., *芪冬润肺汤治疗气阴亏虚型慢性咳嗽39例临床观察.* 河北中医, 2015. **37**(02): p. 174-176.

83. 李赞峰, 刘玉萍, and 高明, *金玄利咽颗粒治疗成人慢性咳嗽的疗效观察.* 临床合理用药杂志, 2011. **4**(34): p. 74.

84. 李静静, 衡永波, and 王强, *止咳平喘汤辅治儿童慢性咳嗽变异性哮喘疗效观察.* 实用中医药杂志, 2020. **36**(12): p. 1616-1617.

85. 李颉, et al., *化痰祛风方治疗小儿呼吸道感染后慢性咳嗽(痰湿证)的临床研究.* 上海中医药杂志, 2020. **54**(10): p. 62-67.

86. 苗良, *参苓汤治疗慢性咳嗽64例.* 实用中医内科杂志, 2008(09): p. 19.

87. 武志娟, 张大鹏, and 张志敏, *麻黄附子细辛汤加味治疗慢性咳嗽65例观察.* 实用中医药杂志, 2011. **27**(10): p. 671.

88. 武胜举, *中西医结合治疗上呼吸道感染后慢性咳嗽的疗效观察.* 实用临床护理学电子杂志, 2019. **4**(08): p. 18+20.

89. 武鹏, *补肺汤联合西药治疗慢性咳嗽的效果及对咳嗽积分的影响.* 黑龙江中医药, 2021. **50**(05): p. 165-166.

90. 潘奇林, 余洋, and 李春玲, *加味六君子汤治疗慢性咳嗽40例疗效观察.* 云南中医中药杂志, 2012. **33**(05): p. 82.

91. 潘方红, *中西医结合治疗慢性咳嗽的临床效果研究.* 东方药膳, 2020(16): p. 267.

92. 潘玉梅, 杨若莹, and 瞿慧, *加味二陈汤治疗小儿痰湿蕴肺型慢性咳嗽的临床疗效观察.* 临床医药文献电子杂志, 2019. **6**(90): p. 26.

93. 方小云, *中药胶囊治疗慢性咳嗽的临床研究.* 大家健康（中旬版）, 2017. **11**(5): p. 40-41.

94. 方俊成, 黎业鹏, and 王志伟, *三参二梗汤联合西药治疗气阴两虚型慢性咳嗽的临床效果观察.* 中国中医药科技, 2021. **28**(01): p. 145-147.

95. 房志家, *止嗽散加减合苓甘五味姜辛汤治疗慢性咳嗽的临床价值分析.* 大家健康(学术版), 2015. **9**(23): p. 41-42.

96. 白散丹 and 苏木亚, *蒙药治疗慢性咳嗽临床观察.* 中国民族医药杂志, 2020. **26**(1): p. 4-5.

97. 白雪梅, et al., *中西医结合治疗小儿慢性咳嗽临床研究.* 亚太传统医药, 2015. **11**(14): p. 79-80.

98. 樊敏, *碧玉止咳汤治疗顽固性干咳60例——附西药治疗30例对照.* 浙江中医杂志, 2001. **36**(4): p. 151.

99. 樊志明, *射干麻黄汤合用西药治疗咳嗽变异型哮喘39例.* 中国中医药科技, 2008. **15**(5): p. 392-393.

100. 樊亚军, 车红英, and 郑志远, *以慢性咳嗽为主要表现的儿童鼻后滴漏综合征诊断和治疗效果探讨.* 家庭医药.就医选药, 2020(9): p. 13-14.

101. 樊学扬, *苓甘五味细辛汤联合西药治疗慢性咳嗽.* 养生保健指南, 2019(11): p. 243.

102. 范伏元 and 罗姣利, *自拟疏风宣肺汤治疗慢性咳嗽300例临床观察.* 中医药导报, 2006(02): p. 33-34+43.

103. 范国梅, *止嗽散加减治疗慢性咳嗽的效果及安全性分析.* 北方药学, 2021. **18**(08): p. 158-159.

104. 扶佳玲, *健脾止咳汤治疗慢性咳嗽的效果评价.* 当代医药论丛, 2020. **18**(03): p. 4-5.

105. 付茹, et al., *自拟清肺汤联合西药治疗慢性咳嗽疗效观察.* 西部中医药, 2019. **32**(05): p. 94-96.

106. 常英丽, *观察自拟益气活血肃肺汤治疗小儿慢性咳嗽（肺脾气虚型）的临床有效性分析.* 今日健康, 2016. **15**(2): p. 332-332.

107. 沙艳萍, *半夏厚朴汤合麦门冬汤治疗慢性咳嗽的效果探析.* 当代医药论丛, 2020. **18**(12): p. 186-187.

108. 史锁芳, *清润止咳颗粒治疗外感久咳116例临床观察.* 国医论坛, 2001. **16**(3): p. 23-24.

109. 徐琴, 吴峰, and 徐宪韬, *蓝芩口服液治疗青年上气道咳嗽综合征引起的慢性咳嗽临床观察.* 河北中医, 2016. **38**(10): p. 1511-1514.

110. 徐莉, *止嗽散加减治疗感冒后咳嗽临床观察.* 中国农村卫生, 2015(3): p. 47-47,49.

111. 徐永红, *加味二陈汤治疗小儿痰湿蕴肺型感染后咳嗽临床研究.* 亚太传统医药, 2017. **13**(8): p. 148-149.

112. 胥媛, *养阴清肺汤联合孟鲁司特治疗55例小儿支原体肺炎后慢性咳嗽的效果评价.* 河南医学研究, 2017. **26**(01): p. 144-145.

113. 徐菁, *沙参麦冬汤合芍药甘草汤治疗肺炎支原体感染后小儿慢性咳嗽60例.* 中国中医药科技, 2012. **19**(02): p. 101.

114. 徐春霞, *百合固金汤加减治疗虚火灼肺型慢性咳嗽的临床疗效.* 内蒙古中医药, 2019. **38**(10): p. 15-16.

115. 宣旎, *化痰祛风方治疗小儿呼吸道感染后慢性咳嗽(痰湿证)的临床效果.* 内蒙古中医药, 2021. **40**(8): p. 19-20.

116. 薛武, 吴平, and 李志勇, *中西医结合治疗慢性咳嗽临床观察.* 健康之友, 2020(20): p. 36-37.

117. 徐亚琴, *加味止嗽散治疗小儿慢性咳嗽临床观察.* 大家健康(学术版), 2013. **7**(11): p. 75.

118. 徐传藩, 吴怀敏, and 徐盼, *苓甘五味姜辛汤合止嗽散加减治疗慢性咳嗽的疗效观察.* 内蒙古中医药, 2020. **39**(04): p. 57-58.

119. 胥卫青 and 戴伲伲, *滋阴止咳汤治疗小儿慢性咳嗽的临床分析.* 饮食保健, 2020. **7**(22): p. 99-100.

120. 徐响瑜, 蔡淑琴, and 俞素青, *自拟宣肺汤合六君子汤治疗慢性咳嗽的临床观察.* 中国中医药科技, 2021. **28**(03): p. 469-470.

121. 徐爱民, *止嗽散加减合苓甘五味姜辛汤用于慢性咳嗽治疗的疗效研究.* 中西医结合心血管病电子杂志, 2016. **4**(18): p. 158+160.

122. 邵成良, *润肺通络汤联合孟鲁司特钠治疗小儿肺炎支原体感染后慢性咳嗽46例.* 中国中医药科技, 2021. **28**(01): p. 134-135.

123. 邵增才, *止嗽散加减中药汤剂治疗慢性咳嗽的临床疗效观察.* 东方药膳, 2021(10): p. 50.

124. 宋菊芯, *止嗽散治疗感冒后久咳158例.* 中医临床研究, 2010. **2**(24): p. 21-21.

125. 宋雪娟, *中西医结合治疗成人上呼吸道感染后咳嗽的疗效观察.* 中国基层医药, 2012. **19**(21): p. 3302-3303.

126. 宋晓琴, *中西医结合治疗慢性咳嗽的疗效观察.* 临床医药文献电子杂志, 2015. **2**(08): p. 1419-1420.

127. 邵丽, 梁尤娟, and 谢进, *沙参麦冬汤合芍药甘草汤对肺炎支原体感染后小儿慢性咳嗽的治疗效果观察.* 临床合理用药杂志, 2017. **10**(17): p. 70-71.

128. 辛素芳, 李力敏, and 刘佩, *小儿柴桂退热颗粒辅助治疗肺炎支原体感染伴咳嗽变异性哮喘患儿的临床效果观察.* 临床误诊误治, 2020. **33**(1): p. 21-25.

129. 岳会杰, *慢性咳嗽中医治疗效果观察.* 中国农村卫生, 2013(z2): p. 260.

130. 余江秀, *中药小柴胡汤加减治疗难治性慢性咳嗽60例临床效果观察.* 健康必读, 2019(15): p. 183.

131. 余成勇, *苓甘五味姜辛汤合止嗽散加减治疗慢性咳嗽临床观察.* 新中医, 2017. **49**(07): p. 44-46.

132. 倪华开, *中西医结合治疗上呼吸道感染后慢性咳嗽的疗效.* 医学理论与实践, 2020. **33**(18): p. 3019-3020.

133. 伍安平, *中西医结合治疗鼻后滴漏综合征所致慢性咳嗽临床效果评估.* 大家健康(学术版), 2015. **9**(11): p. 90-91.

134. 阮加飞, *麦门冬汤合止嗽散治疗慢性咳嗽36例.* 江苏中医药, 2013. **45**(01): p. 35.

135. 王建华 and 谭庆华, *参苓白术散汤剂联合阿莫西林克拉维在小儿慢性咳嗽的疗效分析.* 湖北科技学院学报(医学版), 2016. **30**(05): p. 414-416.

136. 王桂华, *三拗汤合止嗽散加减治疗慢性咳嗽30例观察.* 实用中医药杂志, 2009. **25**(04): p. 217.

137. 王宏杰 and 杨之藻, *中药煮散剂治疗儿童慢性咳嗽（痰热壅肺证）30例临床观察.* 国医论坛, 2020. **35**(05): p. 26-28.

138. 王琦芬, *自拟温阳汤治疗阳虚质型小儿慢性咳嗽疗效观察.* 中国处方药, 2014. **12**(07): p. 130.

139. 王黎芸, *养阴止嗽汤治疗慢性咳嗽56例.* 云南中医中药杂志, 1999(04): p. 29.

140. 王曼曼, *滋阴止咳汤治疗小儿慢性咳嗽临床观察.* 光明中医, 2019. **34**(16): p. 2508-2510.

141. 王文红, *经方止嗽散加味治疗慢性咳嗽62例临床观察.* 世界最新医学信息文摘, 2019. **19**(40): p. 194+197.

142. 王博, *止嗽散加减治疗呼吸道感染后慢性咳嗽的临床疗效观察.* 临床医药文献电子杂志, 2021. **8**(12): p. 55-57.

143. 王芳, 侯秋雨, and 李明心, *参苓白术散加味治疗肺癌根治术后慢性咳嗽临床疗效及对患者炎性因子及免疫功能的影响.* 四川中医, 2022. **40**(05): p. 76-79.

144. 王步青, et al., *镇肝熄风汤加减联合复方甲氧那明胶囊治疗风盛挛急型慢性咳嗽的临床观察.* 中国民间疗法, 2021. **29**(02): p. 78-81.

145. 王爽, *小青龙汤加减治疗慢性咳嗽的临床效果分析.* 养生保健指南, 2016(25): p. 21-21.

146. 王世强, *橘红痰咳颗粒联合西药治疗慢性咳嗽临床观察.* 新中医, 2014. **46**(09): p. 50-51.

147. 王首, et al., *调理中气法治疗岭南地区小儿慢性咳嗽60例临床观察.* 中医儿科杂志, 2017. **13**(2): p. 36-38.

148. 王燕, *二陈汤联合四君子汤加味治疗46例小儿慢性咳嗽疗效观察.* 光明中医, 2015. **30**(09): p. 1901-1902.

149. 王旭 and 郭楠, *贝蒌止嗽散治疗小儿慢性咳嗽干咳类型疗效观察.* 中国中西医结合儿科学, 2016. **8**(03): p. 319-321.

150. 王云方, *对47例慢性咳嗽患者进行中医辨证治疗的效果探究.* 当代医药论丛, 2018. **16**(22): p. 178-179.

151. 王有广 and 宋吉义, *速效定嗽中药配方颗粒治疗慢性咳嗽108例.* 新中医, 2007(12): p. 66-67.

152. 王子江, *苏黄止咳胶囊联合复方甲氧那明胶囊治疗咳嗽变异性哮喘78例.* 河南中医, 2014. **34**(10): p. 1986-1987.

153. 王展儒, 刘新平, and 张晓颖, *中西医结合治疗肺炎支原体感染所致小儿慢性咳嗽临床研究.* 解放军预防医学杂志, 2019. **37**(09): p. 34-35.

154. 王靖 and 杜文娟, *滋阴止咳汤治疗小儿慢性咳嗽临床研究.* 河北中医, 2016. **38**(05): p. 689-692.

155. 王朝晖, et al., *加服芪冬润肺方治疗气阴亏虚型 慢性咳嗽的疗效观察.* 广西中医药, 2017. **40**(6): p. 22-23.

156. 王俊俊 and 牛永新, *小柴胡汤加减治疗牛慢性咳嗽临床观察.* 中兽医学杂志, 2020(2): p. 7-8.

157. 王俊恒, *止嗽散合三拗汤加减治疗慢性咳嗽的效果及安全性分析.* 健康之友, 2019(18): p. 94-95.

158. 王倩, *小青龙汤合九仙散加减方治疗慢性咳嗽疗效分析.* 临床医药文献电子杂志, 2019. **6**(87): p. 10.

159. 王渝, 武蕾, and 崔丽, *金郁平喘汤加减治疗咳嗽变异型哮喘60例临床观察.* 河北中医, 2008. **30**(3): p. 255-256.

160. 王豹, *疏风宣肺健脾止咳方治疗风盛挛急型慢性咳嗽38例.* 中医研究, 2016. **29**(11): p. 16-17.

161. 王洪, et al., *中药煮散剂健脾益气治疗小儿肺脾气虚型慢性咳嗽的临床观察.* 中国中西医结合儿科学, 2021. **13**(06): p. 526-529.

162. 王洪白, *止嗽散加减治疗慢性咳嗽46例观察.* 实用中医药杂志, 2010. **26**(07): p. 468-469.

163. 汪凤仙 and 汪永和, *加味四逆散治疗胃食管反流病所致慢性咳嗽临床观察.* 新中医, 2014. **46**(07): p. 59-60.

164. 汪凤仙, 曹明祥, and 赵萍, *四逆散加减治疗胃食管反流病引起的慢性咳嗽临床观察.* 新中医, 2015. **47**(12): p. 44-46.

165. 王开新, *参百止咳汤治疗慢性咳嗽130例.* 实用中医药杂志, 1999(01): p. 22.

166. 王晓娥 and 马燕杰, *中西医结合治疗小儿慢性咳嗽疗效分析.* 中国社区医师, 2019. **35**(17): p. 119+122.

167. 王莹, *玉屏风散加减治疗慢性咳嗽的临床研究.* 中国现代药物应用, 2016. **10**(13): p. 33-34.

168. 王评, et al., *麦朴汤治疗慢性咳嗽临床研究.* 中医药临床杂志, 2018. **30**(09): p. 1678-1681.

169. 王锦, *小儿咳嗽的中医临床治疗效果观察.* 医药前沿, 2016. **6**(21): p. 71-71,72.

170. 王飞飞, *中西医结合治疗小儿慢性咳嗽的临床研究.* 特别健康, 2017(21): p. 272.

171. 于世华, *养阴清肺汤配合孟鲁司特治疗小儿支原体肺炎后慢性咳嗽的疗效.* 中国社区医师, 2018. **34**(10): p. 124-125.

172. 郁晓维 and 王明明, *加味玉屏风散治疗小儿慢性咳嗽肺脾两虚证疗效评价.* 中医儿科杂志, 2010. **6**(01): p. 23-27.

173. 熊芳, 冯莉, and 罗华春, *泻白散加减治疗小儿支原体肺炎后久咳临床效果观察.* 中国社区医师, 2020. **36**(7): p. 105-106.

174. 于连达, *小儿咳嗽的中医临床治疗心得.* 临床医药文献电子杂志, 2014(3): p. 355-356.

175. 袁玉, 魏文周, and 杨会双, *中西医结合治疗咳嗽变异性哮喘疗效观察.* 河北中医, 2007. **29**(7): p. 620-620.

176. 袁全军, *养阴清肺汤合止嗽散治疗慢性咳嗽62例.* 养生保健指南, 2017(17): p. 69.

177. 魏琦, *柴胡疏肝散加减治疗慢性咳嗽(肝郁气滞型)的临床疗效观察.* 饮食保健, 2018. **5**(3): p. 78.

178. 游柏稳, 游海, and 杜海燕, *清肝宁肺方治疗嗜酸粒细胞性支气管炎肝火犯肺证的临床观察.* 湖南中医药大学学报, 2011. **31**(9): p. 61-63,67.

179. 尹璐, 秦芳, and 李祖长, *养阴清肺汤合止嗽散对慢性咳嗽的效果观察.* 湖北中医药大学学报, 2018. **20**(02): p. 64-67.

180. 尹鸣, *中药浓煎剂小儿止咳方在慢性咳嗽中的应用体会.* 中西医结合心血管病电子杂志, 2015. **3**(06): p. 77-78.

181. 印长虹, *自拟儿童止嗽汤治疗小儿过敏性咳嗽49例疗效观察.* 中国冶金工业医学杂志, 2016. **33**(1): p. 87-88.

182. 任文辉, *益气化痰汤治疗慢性咳嗽68例.* 河南中医, 2006(07): p. 40-41.

183. 任欢, *中西医结合治疗小儿慢性咳嗽疗效.* 健康大视野, 2022(4): p. 133-134.

184. 岑杨成, *三拗汤合六君子汤化裁治疗小儿慢性咳嗽的临床研究.* 名医, 2019(07): p. 266.

185. 章秀辉, *辛芩颗粒在上气道咳嗽综合征中的临床应用心得.* 内蒙古中医药, 2013. **32**(18): p. 32.

186. 庄秋凤, *小儿消积止咳颗粒联合阿奇霉素治疗小儿肺炎支原体感染后慢性咳嗽疗效观察.* 实用中医药杂志, 2021. **37**(05): p. 819-820.

187. 章匀, 苏成程, and 尤菊松, *中西医结合治疗慢性咳嗽72例临床研究.* 江苏中医药, 2015. **47**(08): p. 42-44.

188. 田路军, *呼吸内科慢性咳嗽症状临床治疗及观察.* 健康必读, 2018(34): p. 271-272.

189. 田亚楠, *西药联合益气补肺汤治疗慢性咳嗽42例疗效观察.* 中国民族民间医药, 2018. **27**(19): p. 99-100.

190. 田国芳, et al., *止嗽散加减治疗慢性咳嗽120例临床观察.* 心理医生, 2016. **22**(23): p. 48-49.

191. 丁丽娟, *止咳化痰汤治疗小儿咳嗽变异性哮喘随机平行对照研究.* 实用中医内科杂志, 2012. **26**(3): p. 28-29.

192. 丁兴荣, *中西医结合治疗儿童上气道咳嗽综合征36例疗效观察.* 基层医学论坛, 2015(6): p. 769-770.

193. 丁晓军, *中西医结合治疗成人咳嗽变异型哮喘36例.* 中国现代药物应用, 2010. **4**(3): p. 166.

194. 丁静, et al., *疏风止咳方治疗咳嗽变异性哮喘45例临床观察.* 河北中医, 2015(6): p. 854-855,856.

195. 刁仕萍, 禹云梅, and 陈志红, *中西医结合治疗高气道反应性慢性咳嗽合并月经失调临床观察.* 中国医药科学, 2014. **4**(15): p. 13-16.

196. 刁志宁, *中医中药治疗慢性咳嗽临床观察.* 中西医结合心血管病电子杂志, 2016. **4**(36): p. 153+156.

197. 曹清, *苓甘五味姜辛汤、西医疗法治疗慢性咳嗽价值研究.* 中国保健营养, 2019. **29**(23): p. 120.

198. 周建国, *42例慢性咳嗽中西医治疗方法.* 健康之路, 2014(12): p. 348-349.

199. 朱金萍, *慢性咳嗽鼻后滴漏综合征中西医诊疗分析.* 健康大视野, 2012. **20**(10): p. 358-358.

200. 朱莎 and 肖波, *陈夏六君汤加减治疗慢性咳嗽的临床观察.* 内蒙古中医药, 2020. **39**(12): p. 19-20.

201. 周燕, *苓桂术甘汤加味治疗54例慢性支气管炎的疗效分析.* 医学信息, 2015(17): p. 251-252.

202. 朱永耀, *益气养阴方为主治疗小儿慢性咳嗽53例.* 陕西中医, 2008. **29**(11): p. 1475-1476.

203. 朱俊波, *苓甘五味姜辛汤合止嗽散加减治疗慢性咳嗽的疗效分析.* 家庭医药, 2019(10): p. 177-178.

204. 朱春花, *自拟清热宣肺祛风利咽方治疗慢性咳嗽风咳证疗效观察.* 中西医结合心血管病电子杂志, 2019. **7**(35): p. 151-152.

205. 朱沈芳, *延年半夏汤治疗慢性咳嗽25例疗效观察.* 浙江中医杂志, 2014. **49**(10): p. 724.

206. 朱苹, *中医辨证治疗慢性咳嗽的有效性及安全性.* 实用妇科内分泌电子杂志, 2020. **7**(03): p. 183-185.

207. 周虹, *中药治疗慢性咳嗽疗效观察.* 健康必读, 2021(4): p. 41.

208. 仲乐年, *自拟清肺汤联合西药治疗慢性咳嗽疗效分析.* 特别健康, 2020(5): p. 83.

209. 朱丽, *探讨中西医结合治疗上呼吸道感染后慢性咳嗽的效果.* 健康必读, 2018(22): p. 29-30.

210. 朱婴, *桑芩汤治疗慢性支气管炎痰热郁肺证.* 长春中医药大学学报, 2018. **34**(1): p. 111-113.

211. 周进 and 杨宇星, *小青龙汤治疗老年慢性咳嗽体会.* 人人健康, 2017(10): p. 117.

212. 朱青芝 and 闫莉, *加味六君子汤治疗高原地区小儿肺炎恢复期56例.* 四川中医, 2010. **28**(9): p. 84-85.

213. 周静, *小儿肺咳颗粒与盐酸氨溴索糖浆治疗小儿慢性咳嗽临床对比研究.* 新中医, 2021. **53**(05): p. 120-123.

214. 曾丽君 and 赖婵, *止咳降气汤治疗慢性咳嗽的临床疗效研究.* 深圳中西医结合杂志, 2019. **29**(10): p. 57-59.

215. 智克 and 陈光明, *黄芪止嗽饮治疗小儿肺脾气虚型慢性咳嗽30例.* 安徽中医药大学学报, 2014. **33**(02): p. 42-44.

216. 秦文俊, *慢性咳嗽应用苓甘五味姜辛汤+甲氧那明胶囊治疗的临床分析.* 养生保健指南, 2019(28): p. 282.

217. 秦鉴, et al., *健脾温肺治疗咽痒性久咳的临床研究.* 中药材, 2005. **28**(11): p. 1043-1045.

218. 秦长宏, *中西医结合治疗小儿慢性咳嗽临床分析.* 深圳中西医结合杂志, 2015. **25**(14): p. 55-56.

219. 支献峰, *益气养阴法治疗慢性咳嗽52例疗效观察.* 贵阳中医学院学报, 2010. **32**(6): p. 43-45.

220. 支艳, et al., *滋阴清热法为主治疗慢性咳嗽临床研究.* 中医临床研究, 2014(31): p. 12-13.

221. 蔡彬, *中西医结合治疗慢性咳嗽疗效分析.* 中国当代医药, 2012. **19**(10): p. 126.

222. 蔡娟, *止嗽散加味治疗慢性咳嗽48例观察.* 医药前沿, 2011. **1**(24): p. 328-328.

223. 肖健 and 兰作发, *四逆散加味治疗儿童感冒后咳嗽效果观察.* 中国乡村医药, 2021. **28**(14): p. 25.

224. 楚建业, *祛风开窍方治疗小儿上气道咳嗽综合征29例.* 湖南中医杂志, 2012. **28**(2): p. 67-68.

225. 楚玉波, *升降散加味治疗小儿慢性咳嗽临症体会.* 中医临床研究, 2015. **7**(05): p. 103-104.

226. 肖辉 and 李成伟, *清肝解毒止咳方治疗病毒感染后慢性咳嗽的临床研究.* 世界中医药, 2014(1): p. 36-38.

227. 肖钦 and 居来提·艾买提, *止嗽散合三拗汤加减治疗慢性咳嗽的临床疗效.* 实用中医内科杂志, 2012. **26**(07): p. 24-25.

228. 崔佳宾, et al., *苓甘五味姜辛汤联合三子养亲汤加减治疗慢性咳嗽的临床疗效.* 甘肃科技, 2022. **38**(04): p. 99-101.

229. 崔延英, 赵岩, and 刘小虎, *自拟祛风止嗽汤治疗慢性咳嗽的效果评价.* 中国现代药物应用, 2021. **15**(16): p. 185-187.

230. 崔华, *中药治疗小儿肝火犯肺型慢性咳嗽38例.* 河南中医, 2013. **33**(11): p. 1954-1955.

231. 沈石红 and 吴斐, *苓甘五味姜辛汤联合甲氧那明胶囊治疗慢性咳嗽临床优势对照观察.* 大医生, 2021. **6**(17): p. 74-75.

232. 沈丽萍 and 吕祺美, *止嗽散加减辨位治疗小儿慢性咳嗽45例.* 中国中医药科技, 2017. **24**(04): p. 531-532.

233. 彭文豪, *中西医结合治疗上呼吸道感染后慢性咳嗽的临床效果分析.* 内蒙古中医药, 2016. **35**(01): p. 75-76.

234. 彭伟, *苓甘五味姜辛汤合止嗽散加减治疗慢性咳嗽临床效果及安全性评价.* 中国社区医师, 2018. **34**(21): p. 96-97.

235. 彭红琼, *分析芪冬润肺汤对气阴亏虚型慢性咳嗽患者的治疗效果.* 中国保健营养, 2017. **27**(36): p. 431.

236. 彭继友, *麻杏石甘汤加减治疗呼吸道感染后慢性咳嗽的临床效果观察.* 家庭医药·就医选药, 2021(2): p. 85.

237. 何强, *滋阴止咳汤治疗小儿慢性咳嗽的临床研究.* 光明中医, 2017. **32**(20): p. 2960-2961.

238. 何建, *健脾止咳汤治疗慢性咳嗽临床研究.* 中国社区医师, 2017. **33**(22): p. 105+107.

239. 何明礼, et al., *用二陈汤合三子养亲汤加减对47例痰湿蕴肺型慢性咳嗽患者进行治疗的效果探讨.* 当代医药论丛, 2018. **16**(18): p. 129-131.

240. 何成诗, *养阴祛风治疗慢性咳嗽的临床疗效观察.* 四川中医, 2010. **28**(2): p. 79-80.

241. 夏玉玺, *中西医结合治疗咳嗽变异性哮喘疗效观察.* 深圳中西医结合杂志, 2019. **29**(6): p. 27-29.

242. 何宛芸 and 卢云, *麻黄细辛附子汤加味治疗上气道咳嗽综合征的临床观察.* 中国民族民间医药, 2017. **26**(1): p. 103-106.

243. 夏正飞, *宁嗽汤治疗慢性咳嗽疗效观察.* 中国中医药信息杂志, 1997(11): p. 28.

244. 夏学英, *中西医结合治疗上呼吸道感染后慢性咳嗽的疗效.* 母婴世界, 2021(14): p. 58.

245. 何胜恬, *祛风通窍汤治疗鼻后滴漏型慢性咳嗽112例.* 浙江中医杂志, 2007(07): p. 410.

246. 叶焰, 金华伟, and 李俐, *从痰论治对改善痰浊阻肺型慢性咳嗽生活质量的影响.* 新中医, 2015. **47**(7): p. 44-45.

247. 叶焰 and 里自然, *止嗽散治疗慢性咳嗽60例临床观察.* 中国医药导报, 2009. **6**(29): p. 86+89.

248. 胡崇静, *加味止嗽颗粒治疗风邪恋肺型慢性咳嗽的临床疗效观察*. 2011, 广州中医药大学.

249. 胡玉梅, *止嗽散加减中药汤剂治疗慢性咳嗽的临床疗效观察.* 健康忠告, 2021. **15**(4): p. 65.

250. 胡妮娜, *半夏厚朴汤联合常规西药治疗慢性咳嗽的有效性及复发率.* 现代养生, 2021. **21**(10): p. 44-46.

251. 胡炜, *滋阴止咳汤治疗小儿慢性咳嗽临床观察.* 中国中医药现代远程教育, 2019. **17**(14): p. 42-44.

252. 胡绍贵, 李红杰, and 彭海平, *止嗽散加味治疗慢性咳嗽229例.* 实用中医内科杂志, 2005(06): p. 565.

253. 回云巍 and 宫晓燕, *疏肝和胃、降逆止咳法治疗慢性咳嗽67例临床观察.* 中国医药指南, 2012. **10**(21): p. 604-605.

254. 丛方方 and 朱姬莲, *沙参麦冬汤合芍药甘草汤治疗肺炎支原体感染后小儿慢性咳嗽的分析.* 健康之路, 2018. **17**(01): p. 199-200.

255. 严蕾, *自拟方辨证治疗慢性咳嗽68例的临床效果.* 健康研究, 2018. **38**(4): p. 467-468,473.

256. 严炜, *止嗽散治疗慢性咳嗽临床观察与分析.* 人人健康, 2016(14): p. 80.

257. 乔明飞, *苓甘五味姜辛汤合止嗽散加减治疗慢性咳嗽临床分析.* 光明中医, 2016. **31**(11): p. 1529-1530.

258. 关登明, *中药射干麻黄汤联合复方甲氧那明胶囊治疗咳嗽变异性哮喘的疗效观察.* 实用心脑肺血管病杂志, 2012. **20**(10): p. 1686-1687.

259. 农志飞 and 蒙美禄, *小柴胡汤合苓桂术甘汤治疗儿童慢性咳嗽临床观察.* 辽宁中医杂志, 2012. **39**(10): p. 1999-2000.

260. 冯奕超 and 王志强, *止咳散加减联合盐酸氨溴索颗粒在慢性咳嗽治疗中的应用分析.* 现代诊断与治疗, 2021. **32**(06): p. 850-851.

261. 冯发扬, *滋阴止咳汤治疗小儿慢性咳嗽临床效果观察.* 人人健康, 2019(10): p. 111.

262. 刘金平 and 贾育新, *三焦辨证治疗慢性咳嗽的疗效探讨.* 甘肃科技, 2021. **37**(8): p. 142-144.

263. 刘丹, *升阳益胃汤治疗慢性咳嗽的病案分析.* 中国继续医学教育, 2016. **8**(34): p. 177-178.

264. 刘世贵 and 李欣, *中西医结合法治疗顽固性咳嗽的疗效分析.* 医药前沿, 2014(24): p. 183-184.

265. 刘小燕, *小柴胡汤加味治疗小儿久咳52例临床观察.* 中医临床研究, 2011. **3**(23): p. 66-67.

266. 刘小平, *六君子汤治疗慢性咳嗽60例报道.* 甘肃中医, 2001(06): p. 15-16.

267. 刘玉, *分析小青龙汤加减治疗慢性咳嗽的临床效果.* 内蒙古中医药, 2017. **36**(02): p. 6.

268. 刘雨萌, *止咳散加减治疗慢性咳嗽的临床效果.* 临床医学研究与实践, 2023. **8**(06): p. 105-107.

269. 刘祖强, *中西医结合治疗儿童呼吸道感染后慢性咳嗽50例.* 江西中医药, 2010. **41**(05): p. 33-34.

270. 刘中友 and 陈丽华, *苓甘五味姜辛汤联合甲氧那明胶囊治疗慢性咳嗽疗效观察.* 实用中医药杂志, 2019. **35**(02): p. 204-205.

271. 刘春晓, *加味六君子汤治疗西宁地区小儿肺炎恢复期96例.* 高原医学杂志, 2009. **19**(3): p. 55-56.

272. 刘通, et al., *温肺化饮法治疗外寒内饮型慢性咳嗽疗效观察.* 饮食科学, 2019(08): p. 72.

273. 刘东宇 and 孔宪军, *止嗽降气汤治疗慢性咳嗽的临床分析.* 中国保健营养, 2019. **29**(33): p. 342.

274. 刘会智, 刘丽, and 陈也, *苓甘五味姜辛汤联合复方甲氧那明治疗慢性咳嗽临床研究.* 西南国防医药, 2017. **27**(06): p. 585-588.

275. 刘兴茂 and 尚红果, *88例慢性咳嗽的临床诊治体会.* 中国保健营养, 2018. **28**(21): p. 103.

276. 刘华蓉 and 刘尚义, *中医辨证论治20例难治性慢性咳嗽.* 贵州医药, 2010. **34**(07): p. 658-659.

277. 刘岚 and 谭梅军, *中西医结合治疗小儿慢性咳嗽临床探究.* 深圳中西医结合杂志, 2020. **30**(03): p. 22-23.

278. 刘晓玲 and 李娜, *半夏泻心汤加减治疗慢性咳嗽的效果评价.* 名医, 2019(11): p. 237.

279. 刘晓乐, *儿童慢性咳嗽的中西医治疗.* 当代临床医刊, 2018. **31**(3): p. 3896-3897.

280. 刘洁, *中西医结合治疗慢性咳嗽临床观察.* 实用中医药杂志, 2019. **35**(09): p. 1135.

281. 刘润, *沙参玉竹汤加减治疗慢性咳嗽的临床效果研究.* 健康之友, 2022(20): p. 230-231,234.

282. 刘瑶, *中西医结合治疗慢性咳嗽68例观察.* 实用中医药杂志, 2015. **31**(08): p. 750.

283. 刘莹, 刘继东, and 刘琳, *中西医结合治疗慢性咳嗽效果观察.* 中外医学研究, 2018. **16**(30): p. 166-168.

284. 刘鑫 and 刘小红, *探讨小青龙汤加减治疗慢性咳嗽的临床疗效.* 母婴世界, 2020(30): p. 112.

285. 刘钰斌, et al., *中医辩证治疗慢性咳嗽的临床疗效研究.* 临床医药文献电子杂志, 2020. **7**(48): p. 78-79.

286. 刘颖, *中药颗粒治疗慢性咳嗽的临床效果分析.* 内蒙古中医药, 2016. **35**(14): p. 45.

287. 卢斌 and 黄慧晖, *探讨中西医结合治疗慢性咳嗽40例的疗效.* 中医临床研究, 2018. **10**(15): p. 22-23+28.

288. 卢云 and 赖文静, *祛风化痰、肺鼻同治法治疗鼻后滴流综合征240例临床疗效观察.* 现代中医临床, 2014. **21**(3): p. 30-33.

289. 卢欣 and 李文, *麻龙定喘汤治疗小儿慢性咳嗽的临床疗效观察.* 养生保健指南, 2021(7): p. 122.

290. 卢转红, *中医辨证治疗慢性咳嗽的临床效果观察.* 临床合理用药杂志, 2019. **12**(02): p. 53-54.

291. 吕秀梅, *自拟祛风止嗽颗粒加减治疗慢性咳嗽342例.* 光明中医, 2015. **30**(09): p. 1899-1900.

292. 吕彩虹 and 彭暾, *加味香砂六君子颗粒治疗小儿肺脾气虚型慢性咳嗽的临床研究.* 内蒙古中医药, 2019. **38**(03): p. 16-17.

293. 吴名德, 何木龙, and 邓宜兴, *大柴胡汤和桃核承气汤加减联合西药治疗慢性咳喘的临床疗效.* 齐齐哈尔医学院学报, 2016. **37**(34): p. 4320-4322.

294. 吴雨平 and 黎秋明, *中西医结合治疗上呼吸道感染后慢性咳嗽的疗效观察.* 广西医学, 2007(02): p. 256-257.

295. 吴丽霞, *益气止咳方治疗慢性咳嗽的疗效观察.* 饮食保健, 2018. **5**(4): p. 86-87.

296. 吴伟伟, *中医治疗儿童感染后慢性咳嗽的临床效果.* 世界最新医学信息文摘, 2016. **16**(23): p. 152+156.

297. 吴剑辉, *中西医结合治疗小儿慢性咳嗽疗效分析.* 广州医药, 2000(03): p. 71-72.

298. 吴静南 and 唐国良, *三拗汤加味治疗慢性咳嗽疗效观察.* 新中医, 2015. **47**(9): p. 56.

299. 喻清和 and 邱志楠, *中西医结合治疗小儿鼻后滴漏致慢性咳嗽疗效观察.* 中医药学刊, 2006(12): p. 2353-2354.

300. 孙建, 侯海慧, and 曹拥军, *杏苏颗粒治疗慢性咳嗽40例临床观察.* 黑龙江中医药, 2014. **43**(06): p. 22-23.

301. 孙娜, *滋阴养肺止咳汤联合阿奇霉素治疗儿童慢性咳嗽的疗效分析.* 中国保健营养, 2020. **30**(36): p. 285.

302. 孙保军, *中西医结合治疗咳嗽变异性哮喘50例.* 中医研究, 2008. **21**(10): p. 28-29.

303. 孙洋, et al., *慢性咳嗽给予半夏泻心汤加减治疗的效果分析.* 饮食保健, 2021(3): p. 81.

304. 孙靖峰, *芪蝉止咳汤联合复方磷酸可待因治疗慢性咳嗽31例.* 河北中医, 2012. **34**(12): p. 1823-1824.

305. 孙志卿, *芪冬润肺方治疗气阴亏虚型慢性咳嗽的临床研究.* 临床医药文献电子杂志, 2016. **3**(2): p. 262-263.

306. 孙亚娟, *止嗽散加减治疗慢性咳嗽的疗效及对血清指标的影响.* 中国民间疗法, 2021. **29**(21): p. 61-63.

307. 孙亚飞, *益气补肺汤联合西药治疗慢性咳嗽43例临床观察.* 中国民族民间医药, 2018. **27**(17): p. 103-105.

308. 宫淑琴 and 郭素香, *中西医结合治疗儿童喉源性咳嗽70例疗效观察.* 河北中医, 2010. **32**(8): p. 1187-1188.

309. 应晶晶, *中西医结合治疗小儿慢性咳嗽的临床研究.* 心理医生, 2015. **21**(20): p. 71-71.

310. 庞家莉, *呼吸内科慢性咳嗽症状临床治疗及观察.* 饮食保健, 2019. **6**(42): p. 27.

311. 张健 and 赵小明, *三拗汤合六君子汤化裁治疗小儿慢性咳嗽的临床疗效.* 临床医学研究与实践, 2018. **3**(26): p. 117-118.

312. 张建忠, *中西医结合治疗慢性鼻窦炎致慢性咳嗽33例分析.* 医学信息, 2015(41): p. 29-30.

313. 张桂英, *止嗽散合三拗汤加减治疗慢性咳嗽的有效性及安全性分析.* 中国保健营养, 2021. **31**(10): p. 250-251.

314. 张雷萍, *中西医结合治疗鼻后滴漏综合征引起的儿童慢性咳嗽30例.* 中国中医药咨讯, 2010. **2**(35): p. 261,263.

315. 张雯, 陈丽萍, and 王伟学, *益气活血肃肺汤治疗小儿慢性咳嗽(肺脾气虚型)的临床研究.* 中华中医药学刊, 2008(02): p. 447-448.

316. 张文仲, *中药治疗儿童咳嗽变异性哮喘.* 实用中西医结合临床, 2006. **6**(4): p. 5-5.

317. 张雯霞, *呼吸内科慢性咳嗽症状的临床观察研究.* 糖尿病天地, 2020. **17**(8): p. 47.

318. 张雪琪 and 周毅, *三拗汤加减联合西药治疗外感后慢性咳嗽疗效研究.* 世界最新医学信息文摘（连续型电子期刊）, 2020. **20**(6): p. 240-242.

319. 张淑英, *小陷胸汤合止嗽散治疗慢性咳嗽临床观察.* 辽宁中医杂志, 2010. **37**(08): p. 1521-1522.

320. 张淑霞, *清肺化痰止咳汤加减治疗慢性咳嗽的临床探讨.* 中外女性健康研究, 2021(23): p. 87-88.

321. 张永丽 and 张俊臻, *中西药合用治疗小儿鼻后滴漏综合征致慢性咳嗽临床观察.* 实用中医药杂志, 2020. **36**(11): p. 1416-1417.

322. 张蕊, *桑栀黛蛤汤治疗肝火犯肺型慢性咳嗽的临床观察.* 中国自然医学杂志, 2009. **11**(04): p. 268-270.

323. 张玉婵, 柏凌云, and 曹文霞, *中西医治疗小儿慢性咳嗽的疗效比较.* 中国全科医学, 2007. **10**(4): p. 321-322.

324. 张一, *苏黄止咳胶囊对慢性咳嗽的疗效分析.* 世界最新医学信息文摘, 2015. **15**(A0): p. 101.

325. 张子燕 and 秦胜娟, *泻肺化痰汤联合孟鲁司特钠治疗非特异性慢性咳嗽痰热壅肺型临床研究.* 实用中医药杂志, 2022. **38**(06): p. 974-975.

326. 张姝, *自拟补益脾肺方治疗肺脾气虚型慢性咳嗽56例临床观察.* 深圳中西医结合杂志, 2017. **27**(1): p. 55-57.

327. 张俊, 倪伟, and 吴淑红, *咳喘六味合剂治疗慢性咳嗽33例.* 光明中医, 2017. **32**(5): p. 650-651.

328. 张中凯, *中药合复方甲氧那明胶囊治疗痰热蕴阻型慢性咳嗽临床疗效探析.* 医药前沿, 2018. **8**(1): p. 327-328.

329. 张志敏 and 王峰, *中医辩证治疗慢性咳嗽的临床疗效研究.* 健康女性, 2022(45): p. 112-113.

330. 张志萍, *射干麻黄汤治疗小儿咳嗽变异性哮喘41例.* 中国中医药现代远程教育, 2013. **11**(19): p. 30-31.

331. 张翠云 and 陈小燕, *自拟益气补肺汤和复方甲氧那敏胶囊治疗肺脾气虚型慢性咳嗽的效果对比.* 内蒙古中医药, 2019. **38**(04): p. 12-13.

332. 张平, 王海东, and 董兰芬, *茯苓白术汤治疗慢性咳嗽58例.* 中国民间疗法, 2003(03): p. 47-48.

333. 张萍 and 张明珠, *中西医结合治疗儿童肺炎支原体感染致慢性咳嗽的疗效分析.* 心理医生, 2015. **21**(18): p. 44-45.

334. 张昊 and 谢永曼, *自拟润肺化痰膏治疗慢性咳嗽60例临床观察.* 中国社区医师, 2015(10): p. 81-81,83.

335. 张劲勋, *补中益气汤治疗慢性咳嗽60例疗效观察.* 中国中医药咨讯, 2011. **3**(10): p. 118.

336. 张国银, *二丁颗粒治疗慢性支气管炎疗效观察.* 河北中医, 2011. **33**(6): p. 911-912.

337. 张烛, 侯飞, and 夏卫良, *中西医结合治疗小儿支气管肺炎43例.* 中国中医药科技, 2021. **28**(2): p. 246-248.

338. 张静, *中医辨证治疗慢性咳嗽的临床疗效及不良反应分析.* 健康大视野, 2021(14): p. 138.

339. 张静 and 冯春丽, *沙参麦冬汤加减方治疗慢性咳嗽的应用及有效性分析.* 医药前沿, 2018. **8**(15): p. 320-321.

340. 张鸿雁, *六君子汤治疗老年慢性咳嗽41例.* 实用中医内科杂志, 2007(03): p. 63.

341. 杨可嘉, *止嗽散治疗慢性咳嗽43例临床观察.* 医学信息, 2015(25): p. 368-368.

342. 杨大海, *陈夏六君汤加减治疗慢性咳嗽的效果和对患者生活质量的影响评价.* 养生大世界, 2021(8): p. 69.

343. 杨玲, *苓甘五味姜辛汤联合西医疗法治疗慢性咳嗽的临床分析.* 药店周刊, 2021(22): p. 131.

344. 杨露, *肃肺止咳汤治疗咳嗽变异性哮喘—慢性咳嗽(风盛挛急证)疗效观察.* 家庭医药, 2019(12): p. 136-137.

345. 杨新平 and 沈娟, *自拟滋阴止咳汤治疗小儿慢性咳嗽的疗效观察.* 人人健康, 2022(26): p. 99-101.

346. 杨永艳, *中西医结合治疗小儿慢性咳嗽的疗效观察.* 中国医药指南, 2016. **14**(01): p. 191.

347. 杨志强, *麦门冬汤加减治疗慢性咳嗽40例.* 实用中医内科杂志, 2011. **25**(12): p. 23-25.

348. 杨芝贵, *清肺止咳汤治疗儿童感染后慢性咳嗽53例临床观察.* 河北中医, 2013. **35**(07): p. 985-986.

349. 杨超, *二陈汤加减治疗慢性咳嗽120例临床观察.* 临床医药文献电子杂志, 2014. **1**(12): p. 2082-2083.

350. 杨春霞, *慢性咳嗽中医治疗效果观察.* 求医问药(下半月), 2012. **10**(09): p. 278-279.

351. 杨春艳, et al., *中西医结合治疗慢性咳嗽体会.* 云南中医中药杂志, 2011. **32**(11): p. 31-32.

352. 杨洪波 and 白丽萍, *地龙三仙饮治疗慢性咳嗽260例.* 基层医学论坛, 2007(16): p. 726.

353. 杨伟岸, *桑柴汤加减治疗慢性咳嗽33例.* 实用中医药杂志, 1999(04): p. 39.

354. 杨凤仙 and 黄振炎, *理中丸治疗慢性咳嗽86例.* 福建中医药, 2004(04): p. 31-32.

355. 杨洁, *补肺止咳方治疗咳嗽变异性哮喘30例临床观察.* 河北中医, 2015(1): p. 42-43,44.

356. 杨赛芬 and 刘海英, *滋阴养肺止咳汤联合阿奇霉素治疗儿童慢性咳嗽的疗效分析.* 中国中医药科技, 2020. **27**(01): p. 154-155.

357. 杨辉, 武志娟, and 张大鹏, *中药二仙饮治疗儿童慢性咳嗽临床观察.* 医学研究杂志, 2011. **40**(05): p. 60-62.

358. 欧书祥, *止嗽散加减治疗慢性咳嗽的有效性临床研究.* 饮食保健, 2018. **5**(15): p. 48-49.

359. 毕慧, *慢性咳嗽给予半夏泻心汤加减治疗的效果分析.* 健康大视野, 2020(1): p. 120.

360. 汤百艳, *慢性咳嗽的中医辨证治疗分析.* 健康大视野, 2018(8): p. 134-135.

361. 温霞, 宁小菊, and 黄宁霞, *通宣理肺汤治疗慢性咳嗽临床观察.* 世界最新医学信息文摘（连续型电子期刊）, 2020. **20**(83): p. 213-214.

362. 温伟波, et al., *中西医结合治疗慢性咳嗽临床体会.* 云南中医中药杂志, 2007(07): p. 17-18.

363. 犹菊, *苓甘五味姜辛汤联合西医疗法治疗慢性咳嗽的临床研究.* 饮食保健, 2019. **6**(24): p. 83.

364. 种博琴, et al., *慢炎平咳方联合西药治疗上气道咳嗽综合征随机平行对照研究.* 实用中医内科杂志, 2017. **31**(2): p. 40-42.

365. 纪燕, 卢云, and 张亚玲, *参苓白术散联合西药治疗慢性咳嗽随机平行对照研究.* 实用中医内科杂志, 2015. **29**(03): p. 127-128.

366. 罗国庆 and 赛力克·吐尔斯巴义, *慢性咳嗽的中医辩证治疗分析.* 医药前沿, 2018. **8**(15): p. 329.

367. 聂付红, *滋阴止咳汤治疗小儿慢性咳嗽临床分析.* 中国保健营养, 2018. **28**(20): p. 280.

368. 苏叶芳, *止嗽散加减治疗慢性咳嗽的临床疗效观察.* 世界最新医学信息文摘, 2018. **18**(91): p. 151.

369. 茆俊卿, *中药颗粒剂(止咳1号)治疗慢性咳嗽的临床研究.* 中医临床研究, 2013. **5**(17): p. 6-7.

370. 荆薇, *应用加味哮咳饮治疗儿童肺炎支原体感染后慢性咳嗽120例临床疗效观察.* 国际感染病学（电子版）, 2020. **9**(3): p. 153-154.

371. 蒋秀英, *小青龙汤联合加巴喷丁治疗难治性慢性咳嗽的效果.* 自我保健, 2021(20): p. 43-44.

372. 蒋雨琪, *三拗汤合六君子汤化裁治疗小儿慢性咳嗽的临床疗效.* 医学食疗与健康, 2019(1): p. 166-167.

373. 许红, *中西医结合治疗咳嗽变异性哮喘40例.* 河南中医, 2007. **27**(7): p. 48-48.

374. 许艳阳, *慢性咳嗽的中医药治疗的分析.* 特别健康, 2019(26): p. 246-247.

375. 谌莹, *加味止嗽散治疗小儿感染后慢性咳嗽的临床观察.* 中医药导报, 2011. **17**(04): p. 44-46.

376. 谢木军 and 谢作权, *桂枝加厚朴杏子汤治疗慢性咳嗽278例.* 实用中医药杂志, 2013. **29**(01): p. 14.

377. 谢玉贤 and 黄晓莉, *自拟祛风止嗽颗粒治疗慢性咳嗽临床效果评价.* 深圳中西医结合杂志, 2019. **29**(05): p. 49-50.

378. 谢中勇, *小儿鼻后滴漏综合征致慢性咳嗽的中西医结合治疗效果分析.* 当代医学, 2014. **20**(22): p. 154-155.

379. 谢波, *疏风止嗽汤治疗慢性咳嗽的临床疗效.* 中国现代医生, 2022. **60**(02): p. 131-133+137.

380. 谭明娜, *益气补肺汤治疗慢性咳嗽的临床研究.* 养生保健指南, 2019(19): p. 295.

381. 谭之能, *呼吸内科慢性咳嗽症状临床特征及治疗体会.* 饮食保健, 2017. **4**(26): p. 153-154.

382. 贺建军, *探讨分析止嗽散合三拗汤加减对慢性咳嗽的临床疗效和用药安全.* 中医临床研究, 2018. **10**(07): p. 111-113.

383. 贺金泉, *80例慢性咳嗽中医治疗效果观察.* 健康必读（中旬刊）, 2013. **12**(12): p. 37.

384. 贾艳平, 元国红, and 梁瑞星, *健脾宣肺方治疗小儿慢性咳嗽的疗效评价.* 深圳中西医结合杂志, 2021. **31**(18): p. 53-55.

385. 赵立新, *参苓白术散加减治疗慢性咳嗽50例.* 实用中医药杂志, 2008(05): p. 286.

386. 赵敏, *中医证象量化积分变化角度评价调理中气法治疗小儿慢性咳嗽的疗效.* 内蒙古中医药, 2019. **38**(09): p. 64-65.

387. 赵影, 袁林, and 练志明, *杏苏散合小柴胡汤加减治疗慢性咳嗽的临床观察.* 中国实用医药, 2020. **15**(12): p. 151-153.

388. 赵春雪, *中西医结合治疗上呼吸道感染后慢性咳嗽的疗效观察.* 养生保健指南, 2020(12): p. 49-50.

389. 赵东凯 and 王檀, *应用乌梅汤治疗慢性咳嗽(脏气虚寒型)60例临床观察.* 中国医学工程, 2010. **18**(03): p. 147.

390. 赵会茹 and 苏春娅, *化痰祛瘀方治疗痰湿犯肺型小儿慢性咳嗽的临床观察及对血清炎性因子的影响.* 中医药信息, 2019. **36**(3): p. 107-110.

391. 赵开华, *小柴胡汤加减治疗慢性咳嗽的临床效果分析.* 饮食保健, 2020(50): p. 39.

392. 赵颖, *中药浓煎剂小儿止咳方治疗慢性咳嗽效果分析.* 中国中医药现代远程教育, 2016. **14**(11): p. 97-98.

393. 连芳, *中西医结合治疗慢性咳嗽42例.* 福建中医药, 2001(01): p. 30-31.

394. 邓健华, *儿咳方治疗小儿慢性咳嗽60例临床观察.* 中国中西医结合儿科学, 2009. **1**(4): p. 352-353.

395. 邓成秀, *加味二陈汤治疗小儿痰湿蕴肺型慢性咳嗽的临床分析.* 中医临床研究, 2017. **9**(01): p. 102-103.

396. 邹晓 and 韩江, *中西医结合治疗气道高反应性慢性咳嗽40例.* 中国中医药科技, 2010. **17**(06): p. 551-552.

397. 郑文龙, *二陈汤+四君子汤加味治疗46例小儿慢性咳嗽疗效观察.* 中医临床研究, 2014. **6**(10): p. 98-99.

398. 郑玉兰, *中医辨证治疗慢性咳嗽疗效观察.* 健康大视野, 2020(13): p. 18.

399. 钟洁梅, *肺脾论治小儿感染后慢性咳嗽的临床疗效分析.* 内蒙古中医药, 2018. **37**(9): p. 6-7.

400. 钱海绵, 张慧, and 柏业军, *润肺汤治疗慢性咳嗽的临床观察.* 中国保健营养（中旬刊）, 2013(8): p. 473-474.

401. 陆晋, *止嗽散合三拗汤加减治疗慢性咳嗽的效果及安全性分析.* 当代医药论丛, 2018. **16**(17): p. 191-193.

402. 陆书琼 and 王宗明, *保和丸合三子养亲汤治疗胃食管反流性咳嗽疗效观察.* 贵阳中医学院学报, 2010. **32**(4): p. 37-39.

403. 陈康桂, 肖波, and 朱康妹, *中医治疗鼻后滴漏综合征所致慢性咳嗽疗效观察.* 医学美学美容（中旬刊）, 2014(10): p. 670-671.

404. 陈健, *杏苏散加减治疗鼻后滴漏致慢性咳嗽疗效观察.* 内蒙古中医药, 2008(15): p. 11.

405. 陈莉, *自拟祛风止嗽颗粒治疗慢性咳嗽.* 医药前沿, 2013(13): p. 334-335.

406. 陈麟, *苓甘五味姜辛汤合止嗽散加减治疗慢性咳嗽的效果.* 健康大视野, 2019(10): p. 97.

407. 陈明静, *芪冬润肺汤治疗气阴亏虚型慢性咳嗽的临床研究.* 中国保健营养, 2020. **30**(20): p. 153.

408. 陈小芳 and 胡晓岚, *中药方剂治疗小儿慢性咳嗽50例临床观察.* 中国社区医师(医学专业), 2012. **14**(32): p. 195.

409. 陈淑婉, *中西医结合治疗慢性咳嗽60例.* 中国中医药现代远程教育, 2013. **11**(15): p. 55.

410. 陈秀丽, *中西结合治疗儿童咳嗽变异性哮喘40例疗效观察.* 中国保健营养（中旬刊）, 2012(10): p. 345.

411. 陈春梅, et al., *养阴清肺汤联合孟鲁司特治疗小儿支原体肺炎后慢性咳嗽效果研究.* 山西医药杂志, 2022. **51**(15): p. 1773-1775.

412. 陈奕庆 and 李珑, *“降气化痰止咳汤”治疗慢性咳嗽266例临床效果观察.* 安徽中医学院学报, 1985(01): p. 10-11.

413. 陈坚志, *养阴通窍利咽汤治疗小儿鼻液后滴综合征48例.* 湖南中医杂志, 2005. **21**(6): p. 56.

414. 陈学晋, *小儿慢性咳嗽124例临床效果观察.* 健康大视野, 2013. **21**(18): p. 415-416.

415. 陈清清, 林国清, and 李学麟, *苓甘五味姜辛汤联合止嗽散加减治疗慢性咳嗽的临床效果.* 中外医疗, 2022. **41**(30): p. 27-30.

416. 陈聪, *慢性咳嗽中医治疗效果观察.* 健康忠告, 2021. **15**(21): p. 149.

417. 韦跃文 and 李志宏, *养阴清肺汤合止嗽散治疗慢性咳嗽疗效观察.* 现代养生, 2017(20): p. 155-156.

418. 韩强, *苓甘五味姜辛汤合止嗽散加减治疗慢性咳嗽临床分析.* 医学食疗与健康, 2019(15): p. 113.

419. 韩俊, *加味二陈汤治疗小儿痰湿蕴肺型慢性咳嗽的疗效分析.* 实用医技杂志, 2016. **23**(11): p. 1239-1240.

420. 顾惠英, *止嗽散治疗慢性咳嗽随机平行对照研究.* 实用中医内科杂志, 2013. **27**(05): p. 39-40.

421. 颜良 and 邢陆英, *中医治疗慢性支气管炎48例临床观察.* 医药前沿, 2017. **7**(31): p. 378-379.

422. 颜蔓仪, *止嗽散合三拗汤加减治疗慢性咳嗽疗效观察.* 亚太传统医药, 2012. **8**(02): p. 73-74.

423. 马世清 and 张玉香, *宣肺止咳颗粒配合西药治疗小儿咳嗽变异性哮喘56例.* 陕西中医, 2012. **33**(7): p. 869-869.

424. 马俊杰 and 周春祥, *小儿久咳方对小儿久咳炎症免疫的调节作用及疗效观察.* 世界科学技术-中医药现代化, 2013(7): p. 1600-1603.

425. 马忠青, 于可战, and 刘金龙, *48例反复呼吸道感染后咳嗽儿童采用玉屏风颗粒联合孟鲁司特治疗的临床疗效分析.* 中国保健营养（上旬刊）, 2014. **24**(3): p. 1589-1590.

426. 马进, *止嗽降气汤治疗慢性咳嗽120例的临床观察.* 内蒙古中医药, 2013. **32**(20): p. 12.

427. 鲁军, *止嗽降气汤治疗慢性咳嗽的临床分析.* 中外医疗, 2018. **37**(13): p. 179-180+183.

428. 麦志广 and 夏鑫华, *天龙咳喘灵组方加味治疗慢性咳嗽146例.* 光明中医, 2009. **24**(1): p. 56-57.

429. 黄冬度, 池坚, and 汤泽生, *平肝清肺汤治疗变异性咳嗽60例观察.* 浙江中医杂志, 2016. **51**(3): p. 180.

430. 黄芳 and 高卫星, *中西医结合治疗气道高反应性慢性咳嗽的疗效.* 包头医学院学报, 2016. **32**(11): p. 102+151.

431. 黄芳, 张纯, and 卢肖霞, *中医辨证治疗慢性咳嗽可行性研究.* 亚太传统医药, 2016. **12**(19): p. 109-110.

432. 黄秀君, *中西医结合治疗小儿慢性咳嗽23例临床观察.* 中医儿科杂志, 2016. **12**(01): p. 40-43.

433. 黄戍成, 童红卫, and 叶圣雅, *射干麻黄汤治疗肺癌根治术后慢性咳嗽的临床观察.* 浙江中医药大学学报, 2012. **36**(04): p. 398+404.

434. 黄淑艳, *肝火犯肺型慢性咳嗽的中医治疗及护理疗效观察.* 养生保健指南, 2018(36): p. 222.

435. 黄珍恺, et al., *祛风止咳汤治疗CVA患者44例临床观察.* 光明中医, 2017. **32**(19): p. 2780-2782.

436. 黄波贞, *半夏厚朴汤合麦门冬汤治疗慢性咳嗽60例临床观察.* 湖南中医杂志, 2018. **34**(11): p. 51-53.

437. 黄慧, 金宁, and 张继友, *止嗽散联合三拗汤加减治疗慢性咳嗽的疗效和安全性分析.* 中国实用医药, 2014. **9**(18): p. 182-183.

438. 黄进, 刘八一, and 邓小敏, *半夏泻心汤化裁治疗慢性咳嗽.* 广西中医学院学报, 2005(02): p. 44-45.

439. 齐密霞, et al., *自拟麻杏汤治疗变异性哮喘慢性咳嗽疗效观察.* 现代中西医结合杂志, 2014. **23**(16): p. 1774-1776.

440. 龙江, *苏黄止咳胶囊治疗慢性咳嗽43例.* 中国中医药现代远程教育, 2013. **11**(20): p. 32-33.

441. Fan, L.F., *Sixty cases of chronic bronchitis at acute stage treated with therapy of integrated traditional Chinese and western medicine.* Henan traditional chinese medicine [he nan zhong yi], 2015. **35**(10): p. 2503‐2504.

442. Mukaida, K., et al., *A pilot study of the multiherb Kampo medicine bakumondoto for cough in patients with chronic obstructive pulmonary disease.* Phytomedicine, 2011. **18**(8-9): p. 625-9.

443. Qin, J., et al., *[Clinical study of strengthen spleen and warm lung on treating chronic cough with throat itching].* Zhong Yao Cai, 2005. **28**(11): p. 1043-5.

444. Zhao Yj, G.B., *Effectiveness observation on inflammation-diminishing and cough-relieving capsule (Xiaoyan Zhike Jiaonang) for senile chronic asthmatic bronchitis patients.* Modern journal of integrated traditional chinese and western medicine, 2007. **16**(19): p. 2679‐2680.

2) not about chronic cough (n = 30)

1. 高龙艳, *止痉润肺汤治疗儿童支原体肺炎后期咳嗽的临床观察.* 健康大视野, 2018(17): p. 98.

2. 金朝晖 and 范伏元, *宣肺止咳汤治疗感冒后咳嗽32例.* 湖南中医杂志, 2011. **27**(3): p. 93-94.

3. 段春香, *中西药结合治疗小儿慢性咳嗽的临床分析.* 吉林医学, 2013. **34**(29): p. 6048-6049.

4. 滕蓓蓓, *小儿消积止咳颗粒联合孟鲁司特钠咀嚼片治疗小儿慢性咳嗽的疗效.* 当代医药论丛, 2021. **19**(17): p. 131-133.

5. 梁刚, et al., *小儿消积止咳口服液对慢性咳嗽患儿症状改善及体液免疫功能的影响.* 世界中医药, 2018. **13**(12): p. 3043-3045,3049.

6. 李宏光, *浅论中医辩证治疗慢性咳嗽的方法与效果.* 当代医药论丛, 2014. **12**(07): p. 52-53.

7. 王寅平, *小五味子汤联合布地奈德治疗风邪伏肺型呼吸道感染后慢性咳嗽的临床观察.* 中国民间疗法, 2022. **30**(02): p. 84-86.

8. 王红玉, *加减清燥救肺汤治疗阴虚燥热型慢性咳嗽46例.* 河南中医, 2015. **35**(04): p. 823-824.

9. 程成龙, *中西医结合治疗儿童鼻后滴漏综合征42例临床观察.* 中医儿科杂志, 2017. **13**(3): p. 53-55.

10. 曾宜干 and 汤燕醒, *探讨中医辨证治疗慢性咳嗽的方法及临床效果.* 内蒙古中医药, 2016. **35**(14): p. 28-29.

11. 蔡安琼, *养阴清肺汤改善阴虚肺热证小儿肺炎恢复期慢性咳嗽的临床效果.* 中外医学研究, 2021. **19**(17): p. 51-53.

12. 蔡霞, *中医辩证治疗慢性咳嗽的临床疗效研究.* 健康必读, 2021(28): p. 235.

13. 洪志丹, 陈建武, and 徐承红, *祛风解痉平哮汤联合西医常规治疗支气管哮喘风哮证32例.* 中国中医药科技, 2021. **28**(4): p. 606-608.

14. 严大樑 and 李建军, *止嗽散联合西药治疗外感顽固性咳嗽随机平行对照研究.* 实用中医内科杂志, 2017. **31**(7): p. 46-47.

15. 刘坤 and 陈超, *桑菊饮加减治疗小儿肺炎支原体感染后慢性咳嗽的疗效观察.* 世界中西医结合杂志, 2022. **17**(09): p. 1811-1814.

16. 吴启富, 赵将, and 吴江华, *疏风解毒胶囊联合常规治疗上气道咳嗽综合征的疗效观察.* 中国药师, 2018. **21**(5): p. 856-858.

17. 张雷英, *止嗽散合黛蛤散治疗外感后慢性咳嗽肝火犯肺证40例.* 中国中医药科技, 2018. **25**(06): p. 896-898.

18. 张茵, *滋阴止咳汤治疗小儿慢性咳嗽的临床效果分析.* 中国医药指南, 2019. **17**(21): p. 174-175.

19. 张俊红, *麻杏薏甘汤合止嗽散治疗慢性咳嗽54例.* 中医研究, 2011. **24**(03): p. 19-20.

20. 张丽辉, 张永昌, and 王兵, *养阴清肺法治疗慢性咳嗽38例.* 光明中医, 2016. **31**(2): p. 239-241.

21. 张红, *滋阴止咳汤治疗小儿慢性咳嗽临床分析.* 医药前沿, 2018. **8**(4): p. 331.

22. 杨梅玉, *麻杏石甘汤治疗呼吸道感染后慢性咳嗽的临床效果.* 中国当代医药, 2022. **29**(08): p. 141-143.

23. 杨胜利, 颜昭君, and 赵家亮, *活血利咽方治疗慢性咳嗽疗效观察.* 中国中医药信息杂志, 2013(12): p. 72-72,73.

24. 谢顺英, *自拟党参固金汤治疗外感后顽固性咳嗽临床观察.* 四川中医, 2015. **33**(8): p. 91-93.

25. 赵生华, *上呼吸道感染后慢性咳嗽患者行中西医结合治疗临床疗效观察.* 母婴世界, 2018(10): p. 76.

26. 陈安, *益气补肺汤辅助治疗慢性咳嗽的疗效分析.* 系统医学, 2021. **6**(11): p. 156-158.

27. 陈玉琴, *小儿支原体肺炎后慢性咳嗽应用养阴清肺汤联合孟鲁司特治疗的效果分析.* 中国保健营养, 2019. **29**(13): p. 361.

28. 马建良, *中西医结合治疗小儿胃食管反流所致慢性咳嗽的疗效观察.* 中国中西医结合儿科学, 2011. **3**(01): p. 61-62.

29. 马志杰, *保肺滋肾汤联合西药治疗慢性咳嗽46例.* 中医研究, 2015. **28**(11): p. 33-34.

30. Wang, H.Y., *46 cases with chronic cough of yin deficiency and dryness-heat pattern treated by modified dryness-clearing and lung-saving decoction.* Henan traditional chinese medicine [henan zhong yi], 2015. **35**(4): p. 823‐824.

3) not reporting cough period (n = 33)

1. 姜晓兰, *支原体肺炎后慢性咳嗽应用养阴清肺汤效果探讨.* 康颐, 2020(9): p. 247.

2. 霍健, et al., *参苓白术散加减对肺癌术后慢性咳嗽的疗效及相关机制研究.* 世界中医药, 2019. **14**(09): p. 2300-2304.

3. 金燕, *中医辨证治疗长期住院的老年慢性支气管炎的临床疗效分析.* 饮食保健, 2020(45): p. 99.

4. 李聪敏, et al., *芪冬润肺方治疗气阴亏虚型慢性咳嗽疗效观察.* 河北医药, 2015(2): p. 214-215.

5. 王秀杰, *中医辨证治疗慢性咳嗽的方法与效果探讨.* 中医临床研究, 2017. **9**(09): p. 15-16.

6. 王婷婷, 王增峰, and 李卫妮, *加味麦味地黄汤治疗小儿慢性咳嗽阴虚肺热证30例的疗效观察.* 特别健康, 2020(33): p. 74-75.

7. 任清发 and 李建明, *中西医结合治疗上呼吸道感染后慢性咳嗽的效果观察.* 中西医结合心血管病电子杂志, 2018. **6**(24): p. 171.

8. 朱文亮, *芪冬润肺汤治疗气阴亏虚型慢性咳嗽38例临床研究.* 饮食保健, 2016. **3**(8): p. 83-84.

9. 朱婴, *自拟益气补肺汤治疗肺脾气虚型慢性咳嗽临床观察.* 四川中医, 2017. **35**(06): p. 166-168.

10. 杭文璐, et al., *苏黄止咳胶囊治疗慢性咳嗽临床观察.* 光明中医, 2020. **35**(23): p. 3678-3681.

11. 夏珑娟, *杏苏散合小柴胡汤加减治疗慢性咳嗽的临床观察.* 东方药膳, 2021(17): p. 110.

12. 叶丹丹 and 毛敏华, *加味三拗汤联合孟鲁司特钠片治疗咳嗽变异性哮喘疗效观察.* 新中医, 2015. **47**(7): p. 48-50.

13. 叶燕琼, *145例慢性咳嗽的临床诊治体会.* 中国保健营养, 2017. **27**(30): p. 132-133.

14. 兰森宁, *止嗽散加减治疗肺结核慢性咳嗽的效果观察.* 中国冶金工业医学杂志, 2020. **37**(02): p. 205-206.

15. 冯彩云, *贪念加味二陈汤治疗小儿痰湿蕴肺型慢性咳嗽的临床效果.* 饮食保健, 2021(42): p. 128-129.

16. 刘靖, et al., *疏风宣肺法治疗慢性咳嗽30例的临床观察.* 临床医药文献电子杂志, 2017. **4**(3): p. 533.

17. 刘超群, *中医活血通络搜风止咳方联合西药治疗慢性咳嗽的临床效果.* 中国社区医师, 2018. **34**(18): p. 83-84.

18. 刘春, *中医治疗慢性咳嗽临床疗效分析.* 大家健康（中旬版）, 2018. **12**(4): p. 25-26.

19. 张雷, *中西医结合治疗气道高反应性慢性咳嗽的疗效探究.* 健康前沿, 2019. **28**(2): p. 226.

20. 张峰, *止嗽散加减治疗慢性咳嗽80例临床观察.* 世界最新医学信息文摘, 2017. **17**(65): p. 192.

21. 罗建君, et al., *升阳益胃法治疗肺脾两虚型肺癌患者慢性咳嗽的临床研究.* 大医生, 2023. **8**(3): p. 81-84.

22. 贾温春, *辛汤合止嗽散加减治疗慢性咳嗽临床效果分析.* 世界最新医学信息文摘, 2018. **18**(65): p. 159.

23. 赵明月, *中西医联合治疗慢性咳嗽及其临床疗效观察.* 卫生职业教育, 2014. **32**(16): p. 124-125.

24. 郑子琦, et al., *探索中成药治疗儿童咳嗽变异性哮喘的有效性与安全性——小儿咳喘颗粒Ⅱ期临床试验方案设计.* 药物评价研究, 2015. **38**(5): p. 527-532.

25. 陈四文 and 高树彬, *益气清鼻散治疗鼻源性咳嗽40例.* 南京中医药大学学报（自然科学版）, 2008. **24**(3): p. 163-164.

26. 陈军红, 张阳阳, and 韩思, *玉屏风颗粒辅助治疗小儿慢性咳嗽临床研究.* 新中医, 2021. **53**(09): p. 13-16.

27. 陈艳洋, 曾莺, and 邓丽莎, *宣肺通窍方治疗小儿鼻源性慢性咳嗽的疗效观察.* 广东医学院学报, 2016. **34**(1): p. 87-89.

28. 韦江红, *苓甘五味姜辛汤合止嗽散加减治疗慢性咳嗽的疗效及不良反应发生率分析.* 中国保健营养, 2021. **31**(21): p. 258.

29. 黄翠, *健脾祛湿方在慢性咳嗽患者治疗中的应用评价.* 黑龙江医药, 2017. **30**(3): p. 601-603.

30. 黄艳春 and 邓小全, *半夏泻心汤治疗慢性咳嗽42例.* 中国中医药现代远程教育, 2014. **12**(20): p. 145+149.

31. 黄载峰, *滋阴止咳汤治疗小儿慢性咳嗽的效果评价.* 临床医药文献电子杂志, 2019. **6**(19): p. 150.

32. Cao, J. and Q. Hao, *Clinical effect and safety of traditional Chinese medicine Maxing Shigan decoction in the treatment of chronic cough after respiratory tract infection.* Women's Health, 2022(24): p. 58-60.

33. Shah, A.H., et al., *Study of warme shoab muzmin (chronic bronchitis) with therapeutic evaluation of a unani formulation.* Indian journal of traditional knowledge, 2011. **10**: p. 706‐710.

4) not about only HM (n = 1)

1. 刘莉, et al., *肝火犯肺型慢性咳嗽的中医治疗及护理疗效观察.* 湖南中医药大学学报, 2018. **38**(02): p. 220-223.

5) using EATM as control (n = 6)

1. 徐昕, *中西医结合治疗鼻后滴漏综合征所致慢性咳嗽疗效观察.* 河北中医, 2010. **32**(03): p. 378-380.

2. 徐华智 and 李莉, *苓甘五味姜辛汤合止嗽散加减治疗慢性咳嗽的临床疗效.* 深圳中西医结合杂志, 2018. **28**(13): p. 64-65.

3. 侯瀚翔 and 刘归, *升阳益胃汤加减联合止嗽散加味治疗慢性咳嗽的临床效果.* 河南医学研究, 2018. **27**(22): p. 4114-4115.

4. 冯丽英, *半夏厚朴汤治疗对慢性咳嗽患者临床疗效、炎性因子水平及复发率的影响.* 首都食品与医药, 2022. **29**(18): p. 132-134.

5. 刘素容, *慢性咳嗽应用止嗽散合三拗汤加减治疗的临床效果观察.* 保健文汇, 2022. **23**(21): p. 245-247.

6. 闫旭明, *二陈汤加味治疗慢性咳嗽痰浊犯肺型50例临床观察.* 中医临床研究, 2015(4): p. 60-62.

6) not reporting outcome of interest (n = 2)

1. 朱晓萌, *滋阴止咳汤联合孟鲁司特治疗支原体肺炎后慢性咳嗽患儿50例.* 中西医结合研究, 2019. **11**(06): p. 300-301.

2. Cai, L., *The efficacy of the Linggan Wuwei Jiangxin decoction plus Zhisou San on senile chronic cough.* Clinical Journal of Chinese Medicine, 2022. **14**(21): p. 59-62.

7) duplicate data (n = 1)

1. 童玉琴, *自拟理中止嗽散治疗慢性咳嗽80例:附西药疗80例对照.* 中华实用中西医杂志, 2004. **017**(8).

**Supplement 3. Characteristics of included studies**

| **Study ID** | **Funding sources** | **Sample size** | **Mean age** | **Mean cough period** | **Cause of cough** | **Pattern identification** | **(A) HM group** | **(B) Control group** | **Outcome of interest** | **Adverse events** |
| --- | --- | --- | --- | --- | --- | --- | --- | --- | --- | --- |
| Chen 2009 | NR | 78(59:19) | (A) 6.09 ± 3.11 (B) 6.57 ± 2.13 | (A) 8.25 ± 3.23mo (B) 8.25 ± 3.23mo | UACS | Wind-heat invading the lung, Spleen deficiency and excessive phlegm, Kidney yang deficiency | HM + (B) | Loratadine, Roxithromycin ->4yr: Myrtle oil enteric-coated capsules -<4yr: N-acetylcysteine | 1. TER 2. Cough recurrence rate | NR |
| Chen 2014 | NR | 116(58:58) | (A) 42.00 ± 11.20 (B) 41.12 ± 12.34 | (A) 9.34 ± 4.50mo (B) 9.42 ± 4.41mo | Unexplained | None | HM + (B) | Guaifenesin, Pseudoephedrine Hydrochloride, Codeine Phosphate | 1. TER | (A) nausea 1 (B) dizziness 2, nausea 5, nausea and vomiting 4 |
| Chen 2016 | Scientific Research Project of Shenzhen Health and Family Planning System (201506081) | 108(54:54) | (A) 7.7 ± 3.3 (B) 7.5 ± 3.0 | (A) 3.3 ± 1.6mo (B) 2.9 ± 1.9mo | Mixed (CVA, post-respiratory infection) | None | HM + (B) | Ambroterol | 1. TER | NR |
| Chen 2019 | NR | 90(45:45) | (A) 38.6 ± 10.6 (B) 37.8 ± 9.9 | (A) 9.4 ± 2.1mo (B) 9.4 ± 2.3mo | UACS | None | HM + (B) | Montelukast Sodium, Cefuroxime (for sinusitis), Loratadine, Ephedrine Hydrochloride nasal drops | 1. TER 2. LCQ | NR |
| Chen 2021a | NR | 90(45:45) | (A) 7.7 ± 2.6 (B) 7.4 ± 2.1 | (A) 2.8 ± 0.6yr (B) 2.6 ± 0.9yr | Nonspecific | None | HM + (B) | Budesonide, Montelukast Sodium | 1. TER | None |
| Chen 2021b | NR | 78(39:39) | (A) 34.50 ± 1.35 (B) 34.68 ± 1.28 | (A) 2.58 ± 0.42yr (B) 2.60 ± 0.35yr | NR | None | HM | conventional medication | 1. TER | (A) nausea 1 (B) dizziness 3, nausea 2, diarrhea 2, rash 2 |
| Cui 2014 | NR | 50(25:25) | (A) 49.3 ± 3.5 (B) 50.1 ± 4.0 | (A) 3.2 ± 0.7mo (B) 3.1 ± 0.9mo | Mixed (UACS, CVA, GERD) | Wind invading the lung | HM + (B) | -UACS: nasal inhalation of glucocorticoids combined with antihistamines, nasal polyp surgery -CVA: bronchodilators, hormone therapy if necessary -GERD: PPI, H2 receptor blockers, gastric motility drugs | 1. TER | None |
| Cui 2016 | NR | 110(55:55) | 4.3 ± 1.2 | 3.8 ± 1.9mo | CVA | None | HM + (B) | Loratadine | 1. TER 2. Cough recurrence rate | NR |
| Deng 2012 | NR | 160(80:80) | (A) 34 ± 5 (B) 36 ± 4 | (A) 0.8 ± 0.2yr (B) 1.0 ± 0.1yr | GERD | None | HM | Domperidone, Omeprazole | 1. TER | NR |
| Diao 2021 | NR | 100(50:50) | 6.13 ± 2.04 | 5.13 ± 1.03wk | post-respiratory infection | Qi deficiency | HM + (B) | Ambroxol Hydrochloride oral solution | 1. TER | NR |
| Fan 2017 | NR | 196(98:98) | (A) 33.5 ± 2.8 (B) 31.2 ± 1.6 | (A) 11.6 ± 1.9mo (B) 10.1 ± 1.5mo | NR | None | HM | Ambroxol Hydrochloride, Clenbuterol Hydrochloride | 1. TER | NR |
| Gan 2013 | NR | 82(41:41) | 43.6 ± 7.1 | 32 ± 4wk | NR | None | HM + (B) | Ambroxol, Dextromethorphan | 1. TER | NR |
| Gao 2017 | Beijing Administration of TCM (JJ2016-02) | 64(32:32) | (A) 54.7 ± 13.0 (B) 49.6 ± 13.8 | (A) 12.4 ± 7.6mo (B) 10.4 ± 6.9mo | GERD | None | HM + (B) | Rabeprazole capsules, Mosapride Citrate tablets | 1. Cough VAS 2. Chronic Cough Impact Scale 3. TER | NR |
| Ge 2017 | Project of Hebei Institute of Science and Technology Information (20160834) | 86(43:43) | (A) 8.39 ± 1.27 (B) 8.41 ± 1.30 | (A) 6.73 ± 1.05mo (B) 6.69 ± 1.11mo | NR | None | HM + (B) | Nebulized inhalation Budesonide aerosol | 1. TER 2. LCQ | None |
| Gong 2022 | NR | 80(40:40) | (A) 6.42 ± 1.13 (B) 7.42 ± 1.15 | (A) 14.33 ± 2.22wk (B) 14.26 ± 2.28wk | NR | Lung and spleen qi deficiency | HM + (B) | Ipratropium Bromide, Budesonide, (if necessary) Cefixime | 1. TER | NR |
| Gu 2020 | NR | 86(43:43) | (A) 40.31 ± 2.12 (B) 40.27 ± 2.15 | (A) 14.26 ± 1.18wk (B) 14.29 ± 1.15wk | NR | None | HM | Compound Methoxyphenamine, Ambroxol Hydrochloride oral solution, (if necessary) Cephalosporin and other antibiotics | 1. TER | (A)<(B)* (A) dizziness 1, drowsiness 1 (B) dizziness 4, drowsiness 3, nausea 3 |
| Guan 2022 | NR | 96(48:48) | (A) 38.26 ± 3.19 (B) 38.17 ± 3.22 | (A) 7.48 ± 1.19mo (B) 7.45 ± 1.20mo | NR | None | HM + (B) | Ambroxol Hydrochloride tablets | 1. TER 2. Cough recurrence rate | N.S (A) nausea and vomiting 2, headache 2 (B) headache 2, dry mouth 2, nausea and vomiting 2 |
| Han 2008 | NR | 65(35:30) | (A) 35.58 ± 3.57 (B) 37.02 ± 2.42 | (A) 12.57 ± 1.56wk (B) 11.12 ± 1.32wk | Mixed (UACS, CVA, eosinophilic bronchitis, post-respiratory infection) | Lung and spleen qi deficiency | HM | Compound Methoxyphenamine capsules | 1. TER | (A) mild gastrointestinal symptoms 2 (B) None |
| He 2021 | NR | 150(75:75) | (A) 5.8 ± 0.7 (B) 5.5 ± 0.5 | (A) 8.0 ± 0.5wk (B) 8.2 ± 0.4wk | NR | None | HM + (B) | Routine treatment such as anti-infection, expectorant, cough suppressant, and gastric function protection | 1. TER 2. SCS | NR |
| Huang 2021 | NR | 268(134:134) | (A) 36.61 ± 8.02 (B) 36.78 ± 8.43 | (A) 1.11 ± 0.42yr (B) 1.02 ± 0.33yr | NR | None | HM + (B) | Aminophylline, Chlorpheniramine Maleate | 1. TER | NR |
| Hui 2020 | NR | 82(41:41) | (A) 39.28 ± 11.61 (B) 40.25 ± 12.49 | (A) 10.79 ± 1.87wk (B) 10.89 ± 1.93wk | NR | Phlegm-dampness accumulation in the lung, Phlegm-heat stagnation in the lung, Liver fire invading the lung, Lung yin depletion | HM | Cefoxitin Sodium injection, Ambroxol Hydrochloride oral solution | 1. TER 2. SCS 3. LCQ | (A)<(B)* (A) nausea and vomiting 1, flatulence 1 (B) rash 2, flatulence 2, nausea and vomiting 3, dizziness 2 |
| Ji 2022 | NR | 240(120:120) | (A) 38 (B) 39 | (A) 11wk (B) 11.5wk | NR | Cold-heat complex | HM + (B) | Compound Methoxyphenamine capsules | 1. TER 2. SCS | NR |
| Lai 2020 | NR | 50(25:25) | (A) 47.41 ± 6.81 (B) 47.36 ± 6.74 | (A) 2.61 ± 1.18yr (B) 2.65 ± 1.21yr | Mixed (CVA, chronic bronchitis, COPD) | None | HM | Compound Methoxyphenamine capsules | 1. TER | (A)<(B)* (A) dry mouth 1, nausea 1 (B) dizziness 1, dry mouth 2, drowsiness 2, nausea 3 |
| Li 2018 | NR | 84(42:42) | (A) 6.89 ± 1.34 (B) 6.97 ± 1.52 | (A) 8.21 ± 1.65wk  (B) 8.57 ± 1.31wk | NR | None | HM | Ambroterol | 1. TER | NR |
| Li 2019 | NR | 58(29:29) | (A) 5.7 ± 2.3 (B) 5.8 ± 2.3 | (A) 2.5 ± 1.5mo (B) 2.6 ± 1.3mo | post-respiratory infection | Phlegm-heat blocking the lung | HM + (B) | Montelukast Sodium | 1. TER | (A) none (B) diarrhea 1 |
| Li 2020 | NR | 100(50:50) | (A) 35.54 ± 8.24 (B) 37.27 ± 16.73 | (A) 17.35 ± 9.67wk (B) 18.25 ± 9.14wk | NR | Wind pathogen subduing the lung, Lung yin deficiency, Liver fire invading the lung, Stomach qi ascending counterflow | HM | Routine conventional medicine treatment, expectorant, cough suppressant, and appropriate application of anti-infection, anti-inflammation, anti-allergy, airway relaxation, and acid suppression treatment according to the cause | 1. TER 2. Cough recurrence rate | NR |
| Li 2021 | Guangxi Zhuang Autonomous Region Health Department Project (Z2013597) | 93(47:46) | (A) 7.12 ± 5.97 (B) 7.98 ± 5.36 | (A) 12.2 ± 6.8wk  (B) 13.6 ± 5.3wk | Mixed (UACS, CVA, eosinophilic bronchitis, GERD, post-respiratory infection) | Lung and spleen qi deficiency | HM + (B) | Montelukast Sodium | 1. TER 2. SCS | (A)<(B)* (A) chills and fever 1, sore throat 2 (B) nausea and vomiting 3, chills and fever 4, sore throat 3, headache 2 |
| Li 2022 | NR | 100(50:50) | (A) 6.90 ± 1.13 (B) 6.55 ± 1.21 | (A) 1.91 ± 0.21mo (B) 1.51 ± 0.32mo | NR | None | HM + (B) | Bacterial Lysate capsule | 1. TER 2. Cough recurrence rate | N.S (A) gastrointestinal discomfort 2, rash 1, throat discomfort 2, fatigue 1 (B) gastrointestinal discomfort 1, fatigue 1 |
| Liang 2022 | NR | 94(48:46) | (A) 5.94 ± 2.07 (B) 5.63 ± 2.18 | (A) 37.73 ± 5.98d (B) 37.22 ± 6.06d | NR | Spleen deficiency and phlegm dampness | HM | Montelukast Sodium chewable tablets | 1. TER | None |
| Lin 2014 | NR | 66(33:33) | (A) 35.6 ± 1.54 (B) 36.3 ± 1.17 | (A) 0.6 ± 0.42yr (B) 0.5 ± 0.61yr | NR | Lung yin depletion | HM + (B) | Ambroxol Hydrochloride injection | 1. TER | NR |
| Liu 2012 | NR | 60(30:30) | (A) 5.3 ± 1.5 (B) 5.6 ± 1.2 | (A) 2.5 ± 1.0mo (B) 2.3 ± 0.8mo | NR | None | HM | Ambroterol oral solution | 1. TER | NR |
| Liu 2020 | NR | 74(37:37) | 50.18 ± 13.85 | 3.69 ± 0.84yr | NR | None | HM | Ambroxol Hydrochloride tablets | 1. TER | (A)<(B)* (A) nausea 1, vomiting 1, dizziness 1 (B) nausea 4, vomiting 3, dizziness 2, drowsiness 2 |
| Liu 2022 | NR | 86(43:43) | (A) 55.84 ± 3.26 (B) 55.29 ± 3.74 | (A) 3.16 ± 1.47yr (B) 3.28 ± 1.52yr | NR | None | HM | Terbutaline | 1. TER | NR |
| Lu 2013 | Scientific Research Project of Hainan Provincial Health Department (Qiongwei 2011-122) | 198(100:98) | (A) 33 (B) 32 | (A) 2.6mo (B) 2.5mo | NR | None | HM | Codeine Phosphate | 1. TER | (A) none (B) dizziness/nausea 1 |
| Lu 2014 | Scientific Research Project of Hainan Provincial Department of Health (2011-122) | 72(36:36) | (A) 38.2 ± 3.1 (B) 37.3 ± 3.2 | (A) 8.2 ± 2.1mo (B) 8.0 ± 1.9mo | Mixed (UACS, CVA, GERD) | None | HM + (B) | Lidocaine, Aminophylline, Chlorpheniramine | 1. TER | NR |
| Luan 2021 | NR | 86(43:43) | (A) 7.56 ± 1.87 (B) 7.28 ± 1.63 | (A) 1.01 ± 0.32yr (B) 0.98 ± 0.36yr | UACS | None | HM + (B) | Budesonide nasal spray | 1. TER 2. Cough frequency (times/day) | N.S (A) nausea 3, diarrhea 1, dry mouth 1, lack of strength 2, dizziness 3 (B) nausea 4, dry mouth 2, lack of strength 1, dizziness 2 |
| Lyu 2022 | Korea Institute of Oriental Medicine (KSN2021210) | 30(15:15) | (A) 40.47 (B) 39.13 | (A) 40.93mo (B) 36.00mo | GERD | Wind-cold, Phlegm turbidity, Fire-heat, Lung deficiency, Kidney yang deficiency | HM | Placebo | 1. Cough VAS 2. Cough diary score 3. LCQ | ankle sprain 1 (do not report which group) |
| Meng 2020 | Shanghai Three-Year Action Plan Project for the Development of TCM [ZY(2018-2020)-ZWB-1001-CPJS09], for Further Accelerating the Development of TCM [ZY(2018-2020)-FWTX-6030] | 60(30:30) | (A) 49.25 ± 10.19 (B) 47.71 ± 8.74 | (A) 4.84 ± 1.03mo (B) 4.59 ± 1.05mo | NR | Wind-cold invading the lung | HM | Dextromethorphan Hydrobromide, Acetylcysteine for sputum | 1. TER 2. SCS 3. LCQ | None |
| Niu 2020 | NR | 94(47:47) | (A) 5.9 ± 2.0 (B) 5.5 ± 2.2 | (A) 6.9 ± 1.1mo (B) 6.6 ± 1.3mo | post-respiratory infection | None | HM + (B) | Montelukast | 1. TER | NR |
| Shen 2017 | Shanghai Municipal Health and Family Planning Commission TCM Research Fund (2016LQ001) | 80(40:40) | (A) 49.92 ± 9.49 (B) 49.45 ± 9.38 | (A) range 10-46wk  (B) range 9-47wk | NR | None | HM + (B) | Methoxyphenamine capsules | 1. TER 2. SCS | NR |
| Shen 2019 | NR | 80(40:40) | (A) 7.4 ± 1.9 (B) 7.8 ± 2.2 | (A) 12.1 ± 2.3wk (B) 12.9 ± 2.6wk | post-respiratory infection | Phlegm-heat blocking the lung | HM + (B) | Montelukast Sodium chewable tablets, Azithromycin dry suspension | 1. TER | (A)<(B)* (A) abdominal pain 1 (B) abdominal pain 4, abdominal distension 2, diarrhea 1, rash 1 |
| Shi 2010 | Shanghai Key Disciplines (Advantageous Disciplines) Funding Project (Y0302-10) | 90(45:45) | (A) 49.2 ± 9.40 (B) 48.1 ± 9.61 | (A) 12.12 ± 5.82wk (B) 11.55 ± 6.38wk | NR | None | HM | Codeine | 1. TER | None |
| Song 2022 | NR | 158(79:79) | (A) 48.97 ± 1.22 (B) 48.93 ± 1.25 | (A) 3.92 ± 0.71yr (B) 3.98 ± 0.63yr | chronic bronchitis | None | HM + (B) | Ambroxol Hydrochloride oral solution | 1. TER | (A)<(B)* (A) nausea and vomiting 2, dry mouth 1, drowsiness 1 (B) dizziness 4, nausea and vomiting 5, dry mouth 3, drowsiness 4 |
| Sun 2020 | NR | 107(54:53) | (A) 41.36 ± 4.22 (B) 40.61 ± 4.85 | (A) 4.84 ± 0.96mo (B) 5.03 ± 0.78mo | NR | Wind-dryness hurting the lung | HM + (B) | Montelukast Sodium, Bromhexine Hydrochloride | 1. TER | NR |
| Tan 2020 | NR | 60(30:30) | (A) 36.57 ± 2.41 (B) 38.70 ± 2.45 | (A) 5.43 ± 3.14mo (B) 5.56 ± 2.87mo | NR | None | HM + (B) | Compound Methoxyphenamine capsule | 1. SCS | (A) none (B) nausea 1 |
| Tang 2020 | Guangzhou Tianhe District Science and Technology Plan Project (201704KW021) | 82(41:41) | (A) 42.9 ± 14.5 (B) 41.4 ± 15.3 | (A) 11.1 ± 6.6wk (B) 11.5 ± 9.1wk | NR | Wind pathogen subduing the lung, Lung and stomach qi counterflow, Liver fire invading the lung, Phlegm dampness inner exuberance, Lung and kidney yang deficiency, Lung and kidney yin deficiency | HM | Compound Methoxyphenamine capsules, Cetirizine Prednisone, Omeprazole, Domperidone | 1. TER 2. SCS 3. LCQ | NR |
| Tian 2023 | NR | 68(34:34) | (A) 5.04 ± 0.52 (B) 4.90 ± 0.51 | (A) 11.01 ± 2.07wk (B) 10.94 ± 2.04wk | NR | Lung and spleen qi deficiency | HM + (B) | Azithromycin dry suspension, Montelukast Sodium chewable tablets | 1. TER 2. Cough VAS | NR |
| Wang 2012 | NR | 68(34:34) | (A) 39.7 ± 15.3 (B) 38.1 ± 17.4 | (A) 1.4 ± 2.7yr (B) 1.5 ± 3.1yr | Unexplained | Liver cough | HM | Ambroxol | 1. TER | NR |
| Wang 2016a | NR | 150(75:75) | (A) 45.22 ± 2.19 (B) 45.14 ± 2.45 | (A) 3.13 ± 0.45 yr (B) 3.12 ± 0.78 yr | NR | Wind-cold hitting the lung | HM + (B) | Cefuroxime Axetil tablet, Ambroxol Hydrochloride syrup | 1. TER | NR |
| Wang 2016b | NR | 121(61:60) | (A) 45.96 ± 3.98 (B) 46.47 ± 2.74 | (A) 9.18 ± 3.82 wk (B) 10.17 ± 2.66 wk | NR | Phlegm heat accumulation | HM + (B) | Compound Methoxyphenamine capsules | 1. TER | NR |
| Wang 2019 | NR | 108(54:54) | (A) 45.26 ± 8.74 (B) 47.05 ± 9.29 | (A) 4.12 ± 0.50 mo (B) 4.30 ± 0.65 mo | NR | Wind cough | HM | Cetirizine Hydrochloride tablets | 1. TER | NR |
| Wang 2020 | Major Science and Technology Project of Henan Province (112101310200) | 120(60:60) | (A) 6.05 ± 0.9 (B) 6.11 ± 1.03 | (A) 10.31 ± 1.68 wk (B) 10.55 ± 1.92 wk | Mixed (CVA, GERD, post-respiratory infection) | Lung yin deficiency | HM + (B) | Azithromycin (infection), oral β2 receptor agonists (CVA), H2 receptor antagonists and prokinetic drugs (GERD), Mucosolvan | 1. TER 2. SCS | None |
| Wang 2022 | NR | 86(43:43) | (A) 51.28 ± 6.20 (B) 51.34 ± 6.23 | (A) 4.41 ± 1.01mo (B) 4.38 ± 1.02mo | NR | None | HM | Compound Methoxyphenamine capsules | 1. TER 2. SCS | (A)<(B)* (A) nausea 1, dry mouth 1 (B) dizziness 2, nausea 3, drowsiness 2, dry mouth 1 |
| Wu 2019 | NR | 60(30:30) | (A) 52.5 (B) 53.4 | (A) 3.6 ± 1.7mo (B) 3.5 ± 1.6mo | NR | None | HM + (B) | Pentoxyverine tablets, Ambroxol tablets | 1. TER | None |
| Wu 2020 | Anhui Provincial TCM Leading Talent Training Object Project (Chinese Medicine Development Secret [2018] No. 23) | 72(36:36) | (A) 49.92 ± 9.49 (B) 49.45 ± 9.38 | (A) 20.73 ± 11.48 wk (B) 20.64 ± 11.56 wk | NR | None | HM | Compound Methoxyphenamine capsules | 1. TER 2. SCS | (A)<(B)* (A) vomiting 1, dizziness 2 (B) rash 6, vomiting 2, dizziness 3, palpitations 1 |
| Xia 2021 | NR | 114(57:57) | (A) 35.49 ± 4.18 (B) 36.76 ± 4.52 | (A) 12.03 ± 0.56wk (B) 11.85 ± 0.57wk | NR | Wind pathogens hidden in the lung | HM + (B) | Doxofylline tablets | 1. TER | NR |
| Xie 2012 | NR | 44(24:20) | (A) range 21-69 (B) range 24-70 | (A) 2mo-2yr (B) 2mo-3yr | NR | Lung yin depletion | HM | Ambroxol Hydrochloride, Ketotifen, Pentoxyverine | 1. TER | NR |
| Xie 2017 | NR | 96(48:48) | (A) 50.2 ± 3.1 (B) 51.1 ± 3.4 | (A) 3.4 ± 1.2mo (B) 3.6 ± 1.3mo | NR | None | HM | Theophylline sustained-release capsules | 1. TER | NR |
| Xu 2017 | NR | 72(36:36) | (A) 74.7 ± 12.3 (B) 73.6 ± 11.8 | (A) 14.8 ± 4.2wk (B) 13.9 ± 3.7wk | Mixed (UACS, CVA, eosinophilic bronchitis, GERD, post-respiratory infection, allergic cough, drug-induced cough) | Lung yang deficiency | HM + (B) | Nasal inhaled glucocorticoids or antihistamines (UACS), Glucocorticoids or bronchodilators (CVA), Inhaled glucocorticoids (Eosinophilic bronchitis), Domperidone and omeprazole (GERD), Antibiotics and short-term application of glucocorticoids (infection), Antihistamines or glucocorticoids (allergic cough) | 1. TER | NR |
| Yan 2018 | NR | 90(45:45) | (A) 9.87 ± 2.42 (B) 9.52 ± 2.37 | (A) 4.28 ± 1.28wk (B) 4.45 ± 1.66wk | NR | None | HM + (B) | Symptomatic treatment (such as antihistamines, anti-inflammatory, expectorant) | 1. TER 2. SCS 3. LCQ | NR |
| Yang 2012 | NR | 102(51:51) | (A) 39.5 (B) 40.7 | (A) 2-18mo (B) 2-16mo | NR | None | HM | Compound Methoxyphenamine capsules | 1. TER | NR |
| Yang 2019 | Beijing Science and Technology Plan Project (D08050703020802) | 90(45:45) | (A) 41.74 ± 9.08 (B) 38.45 ± 8.13 | (A) 16.8 ± 5.2wk (B) 15.6 ± 4.7wk | NR | Wind-dryness hurting the lung | HM + (B) | Compound Methpheniramine capsules, Ambroxol Hydrochloride dispersible tablets | 1. TER 2. LCQ | NR |
| Yang 2020 | NR | 70(35:35) | (A) 44.36 ± 9.27 (B) 45.53 ± 8.95 | (A) 10.56 ± 4.42wk (B) 9.39 ± 6.37wk | Mixed (UACS, CVA, eosinophilic bronchitis, GERD) | Wind pathogens hidden in the lung | HM + (B) | Doxofylline tablets | 1. TER 2. SCS 3. Cough VAS | NR |
| Yang 2022 | NR | 96(48:48) | (A) 5.02 ± 1.55 (B) 5.13 ± 1.39 | (A) 6.04 ± 0.51wk (B) 6.11 ± 0.63wk | NR | Phlegm dampness | HM | Montelukast Sodium chewable tablets | 1. TER | N.S (A) vomiting 2 (B) vomiting 1, sleep disorder 2 |
| Yi 2020 | NR | 60(30:30) | (A) 39.5 ± 5.9 (B) 40.1 ± 6.2 | (A) 2.9 ± 0.7mo (B) 3.1 ± 0.6mo | Mixed (UACS, CVA, eosinophilic bronchitis, GERD, allergic cough) | None | HM | Compound Methoxyphenamine capsules | 1. TER 2. SCS | (A)<(B)* (A) nausea 1 (B) dizziness 1, nausea 1, palpitation 1, drowsiness 1 |
| Yu 2014 | NR | 60(40:20) | (A) 5.37 ± 4.46 (B) 4.98 ± 3.92 | (A) 2.78 ± 1.45mo (B) 2.32 ± 1.43mo | Mixed (UACS, CVA, GERD, post-respiratory infection) | None | HM | Azithromycin dry mixture, Montelukast Sodium, Aerosol inhalation of glucocorticoids, Domperidone suspension, Compound Pseudoephedrine Hydrochloride oral solution, Ambroxol Hydrochloride oral liquid, and other conventional drug treatment | 1. TER | NR |
| Zhang 2016 | The Youth Science and Technology Fund Project of Nantong Health Bureau, Jiangsu Province (WQ2014066) | 90(30:30:30) | (A1) 38.41 ± 6.95 (A2) 28.51 ± 7.57 (B) 30.52 ± 5.31 | 10wk | Unexplained | Liver fire invading the lung | (A1) HM (A2) HM + (B) | Ambroxol Hydrochloride dispersible tablets | 1. TER | None |
| Zhang 2020 | Medical Science and Technology Project of Henan Province (201602123) | 94(47:47) | (A) 7.26 ± 1.09 (B) 7.30 ± 1.11 | (A) 7.05 ± 1.24wk (B) 6.98 ± 1.30wk | NR | None | HM + (B) | Symptomatic treatment, Tulobuterol paste | 1. TER | NR |
| Zhang 2021 | Xu Zhiyin National Famous Chinese Medicine Expert Studio (Ning Wei Finance [2019] No. 26) | 68(34:34) | (A) 50.52 ± 3.49 (B) 51.12 ± 3.41 | (A) 28.56 ± 15.94wk (B) 29.62 ± 16.27wk | NR | None | HM | Compound Methoxyphenamine capsules | 1. TER 2. Cough recurrence rate | (A)<(B)* (A) none (B) anorexia, nausea and vomiting 3, dizziness and palpitation 4 |
| Zhang 2022 | Construction Project of the Third Famous TCM Inheritance Studio in Shaanxi Province | 76(38:38) | (A) 49.72 ± 9.39 (B) 49.46 ± 9.28 | (A) 20.71 ± 11.18wk  (B) 20.64 ± 11.56wk | NR | None | HM + (B) | Methoxyphenamine | 1. TER 2. SCS | (A)<(B)* (A) vomiting 1, palpitation 2 (B) rash 6, vomiting 2, dizziness 3, palpitation 1 |
| Zhao 2022a | NR | 80(40:40) | (A) 5.27 ± 1.68 (B) 5.63 ± 2.12 | (A) 1.56 ± 0.21yr (B) 1.56 ± 0.25yr | NR | None | HM + (B) | Montelukast Sodium tablets, Nebulized Inhalation Budesonide inhalation aerosol | 1. TER 2. Cough recurrence rate | NR |
| Zhao 2022b | Shaanxi Provincial Health Research Fund (2021E013) | 130(65:65) | (A) 7.9 ± 2.4 (B) 8.5 ± 2.5 | (A) 5.0 ± 1.2wk  (B) 5.6 ± 0.8wk | NR | Phlegm dampness accumulation in the lung | HM + (B) | Budesonide, Ipratropium Bromide, Antibiotics | 1. TER | NR |
| Zhao 2022c | NR | 60(30:30) | (A) 46.21 ± 5.37 (B) 46.38 ± 5.61 | (A) 21.88 ± 3.29wk (B) 21.64 ± 3.51wk | NR | None | HM + (B) | Nebulized Budesonide inhalation | 1. TER 2. CSS 3. Cough recurrence rate | NR |
| Zhou 2011 | NR | 84(42:42) | (A) 5.4 ± 2.3 (B) 5.3 ± 2.4 | (A) 3.8 ± 0.8mo (B) 3.6 ± 0.5mo | post-respiratory infection | None | HM + (B) | Roxithromycin Dispersible Tablets | 1. TER | NR |
| Zhou 2017 | Hebei Institute of Science and Technology Information Research Project (20160834) | 88(44:44) | (A) 7.13 ± 1.24 (B) 7.20 ± 1.31 | (A) 4.72 ± 1.03mo (B) 4.68 ± 1.12mo | NR | None | HM + (B) | Compound Methoxyphenamine capsules | 1. TER | NR |
| Zhou 2018 | NR | 100(50:50) | (A) 57.3 ± 6.1 (B) 56.3 ± 5.2 | (A) 1.6 ± 0.3yr (B) 1.7 ± 0.4yr | NR | None | HM + (B) | Compound Methoxyphenamine capsules | 1. TER | (A) dry mouth 1, nausea 1, drowsiness 1 (B) dry mouth 1, nausea 1 |
| Zhou 2021 | Hainan Provincial Health and Family Planning Industry Research Project (18A200090) | 100(50:50) | (A) 36.4 ± 7.3 (B) 35.9 ± 7.8 | (A) 21.6 ± 6.4wk (B) 21.4 ± 5.3wk | Mixed (UACS, CVA, eosinophilic bronchitis, GERD) | Liver fire invading the lung | HM | Budesonide | 1. TER | (A)<(B)* (A) none (B) mid throat discomfort 5 |
| Zhou 2022 | NR | 86(43:43) | (A) 22.0 ± 3.1 (B) 23.0 ± 2.8 | (A) 9.1 ± 0.8wk (B) 8.9 ± 1.3wk | NR | Cold fluid accumulating in the lung | HM | Ambroxol Hydrochloride oral solution, Dextromethorphan Hydrobromide oral solution, (if necessary) Cephalosporins for anti-infection and aerosol therapy | 1. TER 2. Cough recurrence rate | (A)<(B)* (A) fatigue 1, dizziness 1, dry mouth 1 (B) fatigue 4, dizziness 3, dry mouth 1, nausea 2 |
| Zhu 2012 | NR | 135(68:67) | (A) 46.6 (B) 44.9 | (A) 8.2yr (B) 7.9yr | NR | Lung yin deficiency | HM | Pentoxyverine Citrate tablets | 1. TER | NR |
| Zhu 2017 | NR | 98(49:49) | (A) 47.3 ± 5.1 (B) 46.6 ± 5.7 | (A) 13.9 ± 1.7wk (B) 13.4 ± 1.9wk | Mixed (UACS, CVA, eosinophilic bronchitis, GERD) | None | HM | Compound Methoxyphenamine | 1. TER 2. LCQ | NR |

COPD, chronic obstructive pulmonary disease; CSS, cough symptom score; CVA, cough variant asthma; GERD, gastroesophageal reflux disease; HM, herbal medicine; LCQ, Leicester cough questionnaire; NR, not reported; N.S, not significant between the groups; PPI, proton pump inhibitor; SCS, simplified cough score; TCM, traditional Chinese Medicine; TER, total effective rate; UACS, upper airway cough syndrome; VAS, visual analog scale.

*, p<0.05

**Supplement 4. Details of herbal medicine used in the included studies**

| **Study ID** | **Name of herbal medicine** | **Dosage form** | **Basic herbal medicine prescription (per 1 day)** | **Additional components** | **Pharmaceutical producer** | **Quality control measures reported** | **Chemical analysis reported** | **Administration period** | **Follow-up period** |
| --- | --- | --- | --- | --- | --- | --- | --- | --- | --- |
| Chen 2009 | 1) Wind-heat invading the lung: Sangju-yin 2) Spleen deficiency and excessive phlegm: Erchen-tang 3) Kidney yang deficiency: Shuquan-wan | Decoction | 1) Wind-heat invading the lung: Xanthium strumarium L. [Asteraceae; Xanthii Fructus] 15 g, Forsythia suspensa (Thunb.) Vahl [Oleaceae; Forsythiae Fructus] 12 g, Chrysanthemum × morifolium (Ramat.) Hemsl. [Asteraceae; Chrysanthmi Flos] 10 g, Platycodon grandiflorus (Jacq.) A.DC. [Campanulaceae; Platycodonis Radix] 10 g, Adenophora triphylla (Thunb.) A.DC. [Campanulaceae; Adenophorae Radix] 10 g, Morus alba L. [Moraceae; Mori Folium] 6 g, Mentha canadensis L. [Lamiaceae; Menthae Herba] 6 g, Glycyrrhiza glabra L. [Fabaceae; Glycyrrhizae Radix et Rhizoma] 6 g, Scaphium affine (Mast.) Pierre [Malvaceae; Semen Sterculiae Lychnophorae] 6 g, Cryptotympana dubia (Haupt) [Cicadidae; Cicadidae Periostracum] 6 g 2) Spleen deficiency and excessive phlegm: Fritillaria cirrhosa D.Don [Liliaceae; Fritillariae Cirrhosae Bulbus] 15 g, Perilla frutescens (L.) Britton [Lamiaceae; Perillae Fructus] 12 g, Citrus × aurantium f. deliciosa (Ten.) M.Hiroe [Rutaceae; Citri Unshius Pericarpium] 10 g, Pinellia ternata (Thunb.) Makino [Araceae; Pinelliae Tuber] 10 g, Poria cocos Wolf [Polyporaceae; Poria Sclerotium] 10 g, Prunus armeniaca L. [Rosaceae; Armeniacae Semen] 10 g, Platycodon grandiflorus (Jacq.) A.DC. [Campanulaceae; Platycodonis Radix] 10 g, Raphanus raphanistrum subsp. sativus (L.) Domin [Brassicaceae; Raphani Semen] 6 g, Gleditsia sinensis Lam. [Fabaceae; Gleditsiae Fructus] 6 g, Glycyrrhiza glabra L. [Fabaceae; Glycyrrhizae Radix et Rhizoma] 6 g 3) Kidney yang deficiency: Dioscorea oppositifolia L. [Dioscoreaceae; Dioscoreae Rhizoma] 15 g, Alpinia oxyphylla Miq. [Zingiberaceae; Alpiniae Oxyphyllae Fructus] 12 g, Lindera aggregata (Sims) Kosterm. [Lauraceae; Linderae Radix] 10 g, Atractylodes lancea (Thunb.) DC. [Asteraceae; Atractylodis Rhizoma] 10 g, Terminalia chebula Retz. [Combretaceae; Terminaliae Fructus] 10 g, Punica granatum L. [Lythraceae; Pericarpium Granati] 10 g, Magnolia officinalis Rehder & E.H.Wilson [Magnoliaceae; Magnoliae Flos] 10 g, Prunus mume (Siebold) Siebold & Zucc. [Rosaceae; Mume Fructus] 10 g | NA | NA | None | None | 2 weeks | 1 month |
| Chen 2014 | Bufei-tang | Decoction | Glehnia littoralis (A.Gray) F.Schmidt ex Miq. [Apiaceae; Glehniae Radix] 30 g, Asparagus cochinchinensis (Lour.) Merr. [Asparagaceae; Asparagi Tuber] 15 g, Lilium lancifolium Thunb. [Liliaceae; Lilii Bulbus] 15 g, Schisandra chinensis (Turcz.) Baill. [Schisandraceae; Schisandrae Fructus] 12 g, Polygonatum odoratum (Mill.) Druce [Asparagaceae; Polygonati Odorati Rhizoma] 10 g, Fritillaria cirrhosa D.Don [Liliaceae; Fritillariae Cirrhosae Bulbus] 10 g, Morus alba L. [Moraceae; Mori Radicis Cortex] 10 g, Aster tataricus L.f. [Asteraceae; Asteris Radix et Rhizoma] 10 g, Tussilago farfara L. [Asteraceae; Farfarae Flos] 9 g, Prunus armeniaca L. [Rosaceae; Armeniacae Semen] 9 g, Glycyrrhiza glabra L. [Fabaceae; Glycyrrhizae Radix et Rhizoma] 6 g | 1) Severe cough: add Ephedra sinica Stapf [Ephedraceae; Ephedrae Herba] 6 g, Galanthus nivalis L. [Amaryllidaceae; Lumbricus] 15 g 2) Qi deficiency: add Astragalus mongholicus Bunge [Fabaceae; Astragali Radix] 30 g, Pseudostellaria heterophylla (Miq.) Pax [Caryophyllaceae; Pseudostellariae Radix] 30 g 3) Night sweating: add Triticum aestivum L. [Poaceae; Tritici Fructus Levis] 15 g, Ostrea gigas Thunberg [Ostreidae; Ostreae Testa] 15 g 4) Heat in the palms and soles: add Anemarrhena asphodeloides Bunge [Asparagaceae; Anemarrhenae Rhizoma] 10 g, Phellodendron amurense Rupr. [Rutaceae; Phellodendri Cortex] 10 g 5) Intestinal dryness and constipation: add Trichosanthes kirilowii Maxim. [Cucurbitaceae; Trichosanthis Semen] 15 g, Rheum officinale Baill. [Polygonaceae; Rhei Radix et Rhizoma] 6 g | NA | None | None | 2 weeks | None |
| Chen 2016 | Zhisou-san | Decoction | Aster tataricus L.f. [Asteraceae; Asteris Radix et Rhizoma] 10 g, Stemona tuberosa Lour. [Stemonaceae; Stemonae Radix] 10 g, Tussilago farfara L. [Asteraceae; Farfarae Flos] 10 g, Vincetoxicum stauntonii (Decne.) C.Y.Wu & D.Z.Li [Apocynaceae; Cynanchi Stauntonii Rhizoma Et Radix] 10 g, Scutellaria baicalensis Georgi [Lamiaceae; Scutellariae Radix] 10 g, Galanthus nivalis L. [Amaryllidaceae; Lumbricus] 9 g, Atractylodes lancea (Thunb.) DC. [Asteraceae; Atractylodis Rhizoma] 6 g, Platycodon grandiflorus (Jacq.) A.DC. [Campanulaceae; Platycodonis Radix] 6 g, Glycyrrhiza glabra L. [Fabaceae; Glycyrrhizae Radix et Rhizoma] 6 g, Schisandra chinensis (Turcz.) Baill. [Schisandraceae; Schisandrae Fructus] 6 g | 1) Runny nose: add Zingiber officinale Roscoe [Zingiberaceae; Zingiberis Rhizoma] 2) Phlegm (yellow and sticky): add Fritillaria cirrhosa D.Don [Liliaceae; Fritillariae Cirrhosae Bulbus] | NA | None | None | 3 weeks | None |
| Chen 2019 | Sanren-tang | Decoction | Coix lacryma-jobi var. ma-yuen (Rom.Caill.) Stapf [Poaceae; Coicis Semen] 30 g, Wurfbainia compacta (Sol. ex Maton) Škorničk. & A.D.Poulsen [Zingiberaceae; Amomi Fructus Rotundus] 9 g, Prunus armeniaca L. [Rosaceae; Armeniacae Semen] 15 g, Magnolia officinalis Rehder & E.H.Wilson [Magnoliaceae; Magnoliae Cortex] 15 g, Phyllostachy nigra (Lodd.) Munro var. henonis (Mitf.) Stapf ex Rendle [Gramineae; Bambusae Folium] 12 g, Pinellia ternata (Thunb.) Makino [Araceae; Pinelliae Tuber] 12 g, Talcum 25 g, Tetrapanax papyrifer (Hook.) K.Koch [Araliaceae; Tetrapanacis Medulla] 8 g | 1) Poor dry stool: add Areca catechu L. [Arecaceae; Arecae Semen] 12 g, Trichosanthes kirilowii Maxim. [Cucurbitaceae; Trichosanthis Semen] 12 g 2) Severe cough: add Tussilago farfara L. [Asteraceae; Farfarae Flos] 12 g, Aster tataricus L.f. [Asteraceae; Asteris Radix et Rhizoma] 12 g 3) Loose stool, dampness heavier than heat: add Citrus × aurantium f. deliciosa (Ten.) M.Hiroe [Rutaceae; Citri Unshius Pericarpium] 12 g, Atractylodes lancea (Thunb.) DC. [Asteraceae; Atractylodis Rhizoma] 10 g 4) Heat havier than dampness, sore throat: add Iris domestica (L.) Goldblatt & Mabb. [Iridaceae; Belamcandae Rhizoma] 10 g, Scutellaria baicalensis Georgi [Lamiaceae; Scutellariae Radix] 12 g | NA | None | None | 4 weeks | None |
| Chen 2021a | Erchen-tang combined with Xiaoqinglong-tang | Decoction | Poria cocos Wolf [Polyporaceae; Poria Sclerotium] 10 g, Pinellia ternata (Thunb.) Makino [Araceae; Pinelliae Tuber] 5 g, Citrus × aurantium f. deliciosa (Ten.) M.Hiroe [Rutaceae; Citri Unshius Pericarpium] 5 g, Asarum heterotropoides F.Schmidt [Aristolochiaceae; Asiasari Radix et Rhizoma] 3 g, Schisandra chinensis (Turcz.) Baill. [Schisandraceae; Schisandrae Fructus] 8 g, Paeonia lactiflora Pall. [Paeoniaceae; Paeoniae Radix] 10 g, Glycyrrhiza glabra L. [Fabaceae; Glycyrrhizae Radix et Rhizoma] 5 g, Neolitsea cassia (L.) Kosterm. [Lauraceae; Cinnamomi Ramulus] 10 g, Ephedra sinica Stapf [Ephedraceae; Ephedrae Herba] 4 g, Ziziphus jujuba Mill. [Rhamnaceae; Zizyphi Fructus] 5 pieces, Zingiber officinale Roscoe [Zingiberaceae; Zingiberis Rhizoma] 3 g | 1) Excessive sputum: add Sinapis alba L. [Brassicaceae; Sinapis Semen Alba], Platycodon grandiflorus (Jacq.) A.DC. [Campanulaceae; Platycodonis Radix] 2) Obvious yellow phlegm: add Fritillaria thunbergii Miq. [Liliaceae; Fritillariae Thunbergii Bulbus], Scutellaria baicalensis Georgi [Lamiaceae; Scutellariae Radix], Prunus armeniaca L. [Rosaceae; Armeniacae Semen] 3) Spontaneous sweat, fatigue: add Astragalus mongholicus Bunge [Fabaceae; Astragali Radix], Saposhnikovia divaricata (Turcz. ex Ledeb.) Schischk. [Apiaceae; Saposhnikoviae Radix], Atractylodes lancea (Thunb.) DC. [Asteraceae; Atractylodis Rhizoma] | NA | None | None | 4 weeks | 2 months |
| Chen 2021b | Zhike-san combined with Sanao-tang | Decoction | Vincetoxicum stauntonii (Decne.) C.Y.Wu & D.Z.Li [Apocynaceae; Cynanchi Stauntonii Rhizoma Et Radix] 15 g, Platycodon grandiflorus (Jacq.) A.DC. [Campanulaceae; Platycodonis Radix] 15 g, Scutellaria baicalensis Georgi [Lamiaceae; Scutellariae Radix] 15 g, Stemona tuberosa Lour. [Stemonaceae; Stemonae Radix] 15 g, Galanthus nivalis L. [Amaryllidaceae; Lumbricus] 15 g, Prunus armeniaca L. [Rosaceae; Armeniacae Semen] 15 g, Sesamum indicum L. [Pedaliaceae; Schizonepetae Spica] 15 g, Bombyx mori (Linné) [Bombycidae; Batryticatus Bombyx] 15 g, Ophiopogon japonicus (Thunb.) Ker Gawl. [Asparagaceae; Liriopis seu Ophiopogonis Tuber] 15 g, Aster tataricus L.f. [Asteraceae; Asteris Radix et Rhizoma] 15 g, Citrus × aurantium f. deliciosa (Ten.) M.Hiroe [Rutaceae; Citri Unshius Pericarpium] 10 g, Ephedra sinica Stapf [Ephedraceae; Ephedrae Herba] 10 g, Pinellia ternata (Thunb.) Makino [Araceae; Pinelliae Tuber] 10 g, Glycyrrhiza glabra L. [Fabaceae; Glycyrrhizae Radix et Rhizoma] 5 g | 1) Fever: add Lonicera japonica Thunb. [Caprifoliaceae; Lonicerae Flos] 10 g, Forsythia suspensa (Thunb.) Vahl [Oleaceae; Forsythiae Fructus] 10 g 2) Asthma: add Perilla frutescens (L.) Britton [Lamiaceae; Perillae Fructus] 10 g 3) Cough and yellow phlegm: add Houttuynia cordata Thunb. [Saururaceae; Houttuyniae Herba] 30 g, Scutellaria baicalensis Georgi [Lamiaceae; Scutellariae Radix] 10 g 4) Constipation: add Rheum officinale Baill. [Polygonaceae; Rhei Radix et Rhizoma] 6 g | NA | None | None | 2 weeks | None |
| Cui 2014 | Shufeng Xuanfei-tang | Decoction | Platycodon grandiflorus (Jacq.) A.DC. [Campanulaceae; Platycodonis Radix] 6 g, Sesamum indicum L. [Pedaliaceae; Schizonepetae Spica] 10 g, Poria cocos Wolf [Polyporaceae; Poria Sclerotium] 10 g, Kitagawia praeruptora (Dunn) Pimenov [Apiaceae; Peucedani Radix] 10 g, Ephedra sinica Stapf [Ephedraceae; Ephedrae Herba] 3 g, Citrus × aurantium f. deliciosa (Ten.) M.Hiroe [Rutaceae; Citri Unshius Pericarpium] 10 g, Fritillaria cirrhosa D.Don [Liliaceae; Fritillariae Cirrhosae Bulbus] 6 g, Citrus × aurantium L. [Rutaceae; Aurantii Fructus Immaturus] 10 g, Stemona tuberosa Lour. [Stemonaceae; Stemonae Radix] 10 g, Prunus armeniaca L. [Rosaceae; Armeniacae Semen] 10 g, Pinellia ternata (Thunb.) Makino [Araceae; Pinelliae Tuber] 10 g, Glycyrrhiza glabra L. [Fabaceae; Glycyrrhizae Radix et Rhizoma] 5 g, Mentha canadensis L. [Lamiaceae; Menthae Herba] 6 g | 1) Qi deficiency cough and weakness: add Astragalus mongholicus Bunge [Fabaceae; Astragali Radix] 2) Cough with excessive sputum, white or foamy sputum: add Trichosanthes kirilowii Maxim. [Cucurbitaceae; Trichosanthis Pericarpium], Morus alba L. [Moraceae; Mori Radicis Cortex] 3) Oropharyngeal dryness: add Ophiopogon japonicus (Thunb.) Ker Gawl. [Asparagaceae; Liriopis seu Ophiopogonis Tuber], Adenophora triphylla (Thunb.) A.DC. [Campanulaceae; Adenophorae Radix], Trichosanthes kirilowii Maxim. [Cucurbitaceae; Trichosanthis Radix] 4) Night sweating: add Schisandra chinensis (Turcz.) Baill. [Schisandraceae; Schisandrae Fructus] | NA | None | None | 4 weeks | None |
| Cui 2016 | Zhisou-san | Powder | Platycodon grandiflorus (Jacq.) A.DC. [Campanulaceae; Platycodonis Radix], Sesamum indicum L. [Pedaliaceae; Schizonepetae Spica], Aster tataricus L.f. [Asteraceae; Asteris Radix et Rhizoma], Stemona tuberosa Lour. [Stemonaceae; Stemonae Radix], Vincetoxicum stauntonii (Decne.) C.Y.Wu & D.Z.Li [Apocynaceae; Cynanchi Stauntonii Rhizoma Et Radix] 500 g, Glycyrrhiza glabra L. [Fabaceae; Glycyrrhizae Radix et Rhizoma] 200 g, Citrus × aurantium f. deliciosa (Ten.) M.Hiroe [Rutaceae; Citri Unshius Pericarpium] 250 g (9 g per serving) | NA | NA | None | None | 2 weeks | None |
| Deng 2012 | Miao medicine and Zuojin-wan | Decoction | Aletris spicata (Thunb.) Franch. [Nartheciaceae; Aletris spicata] 10 g, Sinomenium acutum (Thunb.) Rehder & E.H.Wilson [Menispermaceae; Sinomenium Acutum] 10 g, Tengligen 10 g, Sepiella maindroni de Rochebrune [Sepiidae; Sepiae Endoconcha] 10 g, Coptis chinensis Franch. [Ranunculaceae; Coptidis Rhizoma] 6 g, Magnolia officinalis Rehder & E.H.Wilson [Magnoliaceae; Magnoliae Cortex] 6 g, Gardenia jasminoides J.Ellis [Rubiaceae; Gardeniae Fructus] 6 g, Tetradium ruticarpum (A.Juss.) T.G.Hartley [Rutaceae; Evodiae Fructus] 3 g, Valeriana jatamansi Jones ex Roxb. [Caprifoliaceae; Jatamans Valeriana] 2 pieces, Lysionotus pauciflorus var. ikedae (Hatus.) W.T.Wang [Gesneriaceae; Lysionotus pauciflorus Maxim.] 15 g | NA | NA | None | None | 0.5 month | None |
| Diao 2021 | Cansu-yin | Decoction | Codonopsis pilosula (Franch.) Nannf. [Campanulaceae; Codonopsis Pilosulae Radix] 7 g, Perilla frutescens (L.) Britton [Lamiaceae; Perillae Folium] 8 g, Pueraria montana var. lobata (Willd.) Maesen & S.M.Almeida ex Sanjappa & Predeep [Fabaceae; Puerariae Radix] 8 g, Pinellia ternata (Thunb.) Makino [Araceae; Pinelliae Tuber] 5 g, Kitagawia praeruptora (Dunn) Pimenov [Apiaceae; Peucedani Radix] 8 g, Poria cocos Wolf [Polyporaceae; Poria Sclerotium] 8 g, Citrus × aurantium L. [Rutaceae; Aurantii Fructus Immaturus] 7 g, Dolomiaea costus (Falc.) Kasana & A.K.Pandey [Asteraceae; Aucklandiae Radix] 5 g, Citrus × aurantium f. deliciosa (Ten.) M.Hiroe [Rutaceae; Citri Unshius Pericarpium] 7 g, Glycyrrhiza glabra L. [Fabaceae; Glycyrrhizae Radix et Rhizoma] 5 g, Platycodon grandiflorus (Jacq.) A.DC. [Campanulaceae; Platycodonis Radix] 5 g, Aster tataricus L.f. [Asteraceae; Asteris Radix et Rhizoma] 7 g, Stemona tuberosa Lour. [Stemonaceae; Stemonae Radix] 5 g, Prunus mume (Siebold) Siebold & Zucc. [Rosaceae; Mume Fructus] 7 g, Zingiber officinale Roscoe [Zingiberaceae; Zingiberis Rhizoma] 1 piece, Ziziphus jujuba Mill. [Rhamnaceae; Zizyphi Fructus] 1 piece | 1) Sputum yellowish white and sticky: add Houttuynia cordata Thunb. [Saururaceae; Houttuyniae Herba] 10 g, Lonicera japonica Thunb. [Caprifoliaceae; Lonicerae Flos] 7 g 2) Thin white sputum, aggravated by wind: add Ephedra sinica Stapf [Ephedraceae; Ephedrae Herba] 5 g, Neolitsea cassia (L.) Kosterm. [Lauraceae; Cinnamomi Ramulus] 7 g 3) Excessive phlegm: add Morus alba L. [Moraceae; Mori Radicis Cortex] 7 g 4) Unwilling to eat: add Crataegus monogyna Jacq. [Rosaceae; Crataegi Fructus] 7 g, Massa Medicata Fermentata 7 g, Hordeum vulgare L. [Poaceae; Hordei Fructus Germinatus] 7 g, Raphanus raphanistrum subsp. sativus (L.) Domin [Brassicaceae; Raphani Semen] 7 g 5) Severe cough: add Fritillaria cirrhosa D.Don [Liliaceae; Fritillariae Cirrhosae Bulbus] 3 g | NA | None | None | 2 weeks | None |
| Fan 2017 | Zhike Jiangqi-tang | Decoction | Inula japonica Thunb. [Asteraceae; Inulae Flos] 15 g, Bupleurum falcatum L. [Apiaceae; Bupleuri Radix] 12 g, Ephedra sinica Stapf [Ephedraceae; Ephedrae Herba] 10 g, Prunus armeniaca L. [Rosaceae; Armeniacae Semen] 10 g, Citrus × aurantium L. [Rutaceae; Aurantii Fructus Immaturus] 10 g, Citrus trifoliata L. [Rutaceae; Ponciri Fructus Immaturus] 10 g, Tussilago farfara L. [Asteraceae; Farfarae Flos] 10 g, Aster tataricus L.f. [Asteraceae; Asteris Radix et Rhizoma] 10 g, Kitagawia praeruptora (Dunn) Pimenov [Apiaceae; Peucedani Radix] 10 g, Perilla frutescens (L.) Britton [Lamiaceae; Perillae Folium] 10 g, Glycyrrhiza glabra L. [Fabaceae; Glycyrrhizae Radix et Rhizoma] 6 g | NA | NA | None | None | 1 week | None |
| Gan 2013 | Yiqi Yangyin Runfei Zhike-tang | Decoction | Poria cocos Wolf [Polyporaceae; Poria Sclerotium] 10 g, Aristolochia debilis Siebold & Zucc. [Aristolochiaceae; Aristolochiae Fructus] 10 g, Fritillaria cirrhosa D.Don [Liliaceae; Fritillariae Cirrhosae Bulbus] 3 g, Ophiopogon japonicus (Thunb.) Ker Gawl. [Asparagaceae; Liriopis seu Ophiopogonis Tuber] 10 g, Equus asinus Linne [Equidae; Asini Corii Colla] 6 g, Paeonia lactiflora Pall. [Paeoniaceae; Paeoniae Radix] 15 g, Inula japonica Thunb. [Asteraceae; Inulae Flos] 2 g, Schisandra chinensis (Turcz.) Baill. [Schisandraceae; Schisandrae Fructus] 6 g, Aster tataricus L.f. [Asteraceae; Asteris Radix et Rhizoma] 10 g, Prunus armeniaca L. [Rosaceae; Armeniacae Semen] 10 g, Stemona tuberosa Lour. [Stemonaceae; Stemonae Radix] 10 g, Adenophora triphylla (Thunb.) A.DC. [Campanulaceae; Adenophorae Radix] 10 g, Pseudostellaria heterophylla (Miq.) Pax [Caryophyllaceae; Pseudostellariae Radix] 10 g, Glycyrrhiza glabra L. [Fabaceae; Glycyrrhizae Radix et Rhizoma] 5 g | 1) Both hypochondriac pain: add Cucumis melo L. [Cucurbitaceae; Retinervus Luffae Fructus] 10 g, Bupleurum falcatum L. [Apiaceae; Bupleuri Radix] 10 g 2) Sore throat and cramping cough: add Buthus martensii Karsch [Buthidae; Scorpio] 2 pieces, Scolopendra subspinipes mutilans Linné Koch [Scolopendridae; Scolopendra] 2 pieces 3) Stuffy and itchy nose: add Sesamum indicum L. [Pedaliaceae; Schizonepetae Spica] 10 g, Xanthium strumarium L. [Asteraceae; Xanthii Fructus] 10 g, Magnolia officinalis Rehder & E.H.Wilson [Magnoliaceae; Magnoliae Flos] 10 g 4) Chest tightness: add Citrus × aurantium L. [Rutaceae; Aurantii Fructus Immaturus] 10 g, Trichosanthes kirilowii Maxim. [Cucurbitaceae; Trichosanthis Pericarpium] 10 g | NA | None | None | 10 days | None |
| Gao 2017 | Shumu Yuntu Zhike-fang | Decoction | Bupleurum falcatum L. [Apiaceae; Bupleuri Radix] 10 g, Angelica gigas Nakai [Apiaceae; Angelicae Gigantis Radix] 10 g, Paeonia lactiflora Pall. [Paeoniaceae; Paeoniae Radix] 10 g, Citrus trifoliata L. [Rutaceae; Ponciri Fructus Immaturus] 10 g, Poria cocos Wolf [Polyporaceae; Poria Sclerotium] 5 g, Atractylodes lancea (Thunb.) DC. [Asteraceae; Atractylodis Rhizoma] 5 g, Zingiber officinale Roscoe [Zingiberaceae; Zingiberis Rhizoma] 2 g, Gardenia jasminoides J.Ellis [Rubiaceae; Gardeniae Fructus] 3 g, Paeonia × suffruticosa Andrews [Paeoniaceae; Moutan Radicis Cortex] 3 g, Mentha canadensis L. [Lamiaceae; Menthae Herba] 3 g, Fritillaria cirrhosa D.Don [Liliaceae; Fritillariae Cirrhosae Bulbus] 10 g, Morus alba L. [Moraceae; Mori Folium] 10 g, Prunus armeniaca L. [Rosaceae; Armeniacae Semen] 5 g, Adenophora triphylla (Thunb.) A.DC. [Campanulaceae; Adenophorae Radix] 10 g | 1) Obvious reflux and heartburn: add Scapharca subcrenata (Lischke) [Arcidae; Scapharcae seu Tegillarcae Concha] 15 g, Sepiella maindroni de Rochebrune [Sepiidae; Sepiae Endoconcha] 15 g 2) Cannot digest food: add Raphanus raphanistrum subsp. sativus (L.) Domin [Brassicaceae; Raphani Semen] 10 g, Forsythia suspensa (Thunb.) Vahl [Oleaceae; Forsythiae Fructus] 6 g 3) Severe cough: add Kitagawia praeruptora (Dunn) Pimenov [Apiaceae; Peucedani Radix] 6 g, Tussilago farfara L. [Asteraceae; Farfarae Flos] 6 g 4) Chest tightness and suffocation: add Trichosanthes kirilowii Maxim. [Cucurbitaceae; Trichosanthis Semen] 12 g, Allium chinense G.Don [Amaryllidaceae; Allii Macrostemi Bulbus] 3 g 5) Aggravated activities: add Astragalus mongholicus Bunge [Fabaceae; Astragali Radix] 10 g, Codonopsis pilosula (Franch.) Nannf. [Campanulaceae; Codonopsis Pilosulae Radix] 10 g | NA | None | None | 8 weeks | None |
| Ge 2017 | NR | Decoction | Codonopsis pilosula (Franch.) Nannf. [Campanulaceae; Codonopsis Pilosulae Radix] 30 g, Astragalus mongholicus Bunge [Fabaceae; Astragali Radix] 15 g, Poria cocos Wolf [Polyporaceae; Poria Sclerotium] 12 g, Atractylodes lancea (Thunb.) DC. [Asteraceae; Atractylodis Rhizoma] 9 g, Platycodon grandiflorus (Jacq.) A.DC. [Campanulaceae; Platycodonis Radix] 6 g, Citrus × aurantium f. deliciosa (Ten.) M.Hiroe [Rutaceae; Citri Unshius Pericarpium] 6 g, Gallus gallus domesticus Brisson [Phasianidae; Galli Gigeriae Endothelium Corneum] 9 g, Pinellia ternata (Thunb.) Makino [Araceae; Pinelliae Tuber] 6 g, Kitagawia praeruptora (Dunn) Pimenov [Apiaceae; Peucedani Radix] 9 g, Prunus armeniaca L. [Rosaceae; Armeniacae Semen] 12 g, Aster tataricus L.f. [Asteraceae; Asteris Radix et Rhizoma] 9 g, Perilla frutescens (L.) Britton [Lamiaceae; Perillae Folium] 9 g, Glycyrrhiza glabra L. [Fabaceae; Glycyrrhizae Radix et Rhizoma] 3 g | NA | NA | None | None | 1 month | None |
| Gong 2022 | Shenling Baizhu-san combined with Yupingfeng-san | Decoction | Pseudostellaria heterophylla (Miq.) Pax [Caryophyllaceae; Pseudostellariae Radix] 10 g, Poria cocos Wolf [Polyporaceae; Poria Sclerotium] 10 g, Atractylodes lancea (Thunb.) DC. [Asteraceae; Atractylodis Rhizoma] 8 g, Aster tataricus L.f. [Asteraceae; Asteris Radix et Rhizoma] 5 g, Fritillaria thunbergii Miq. [Liliaceae; Fritillariae Thunbergii Bulbus] 10 g, Dioscorea oppositifolia L. [Dioscoreaceae; Dioscoreae Rhizoma] 10 g, Coix lacryma-jobi var. ma-yuen (Rom.Caill.) Stapf [Poaceae; Coicis Semen] 15 g, Stemona tuberosa Lour. [Stemonaceae; Stemonae Radix] 10 g, Polygala senega L. [Polygalaceae; Polygalae Radix] 6 g, Astragalus mongholicus Bunge [Fabaceae; Astragali Radix] 10 g, Saposhnikovia divaricata (Turcz. ex Ledeb.) Schischk. [Apiaceae; Saposhnikoviae Radix] 5 g, Citrus × aurantium f. deliciosa (Ten.) M.Hiroe [Rutaceae; Citri Unshius Pericarpium] 6 g, Glycyrrhiza glabra L. [Fabaceae; Glycyrrhizae Radix et Rhizoma] 10 g, Ziziphus jujuba Mill. [Rhamnaceae; Zizyphi Fructus] 3 pieces | 1) Excessive sweating: add Neolitsea cassia (L.) Kosterm. [Lauraceae; Cinnamomi Ramulus], Paeonia lactiflora Pall. [Paeoniaceae; Paeoniae Radix], Fossilia Ossis Mastodi, Ostrea gigas Thunberg [Ostreidae; Ostreae Testa] 2) Excessive phlegm clear thin: add Pinellia ternata (Thunb.) Makino [Araceae; Pinelliae Tuber], Vincetoxicum stauntonii (Decne.) C.Y.Wu & D.Z.Li [Apocynaceae; Cynanchi Stauntonii Rhizoma Et Radix] 3) Anorexia: add Crataegus monogyna Jacq. [Rosaceae; Crataegi Fructus], Massa Medicata Fermentata, Oryza sativa L. [Poaceae; Oryzae Fructus Germinatus] | NA | None | None | 15 days | None |
| Gu 2020 | Linggan Wuwei Jiangxin-tang combined with Zhisou-san | Decoction | Vincetoxicum stauntonii (Decne.) C.Y.Wu & D.Z.Li [Apocynaceae; Cynanchi Stauntonii Rhizoma Et Radix] 15 g, Poria cocos Wolf [Polyporaceae; Poria Sclerotium] 15 g, Schisandra chinensis (Turcz.) Baill. [Schisandraceae; Schisandrae Fructus] 15 g, Zingiber officinale Roscoe [Zingiberaceae; Zingiberis Rhizoma] 15 g, Aster tataricus L.f. [Asteraceae; Asteris Radix et Rhizoma] 15 g, Pinellia ternata (Thunb.) Makino [Araceae; Pinelliae Tuber] 10 g, Platycodon grandiflorus (Jacq.) A.DC. [Campanulaceae; Platycodonis Radix] 10 g, Glycyrrhiza glabra L. [Fabaceae; Glycyrrhizae Radix et Rhizoma] 10 g, Citrus × aurantium f. deliciosa (Ten.) M.Hiroe [Rutaceae; Citri Unshius Pericarpium] 6 g, Asarum heterotropoides F.Schmidt [Aristolochiaceae; Asiasari Radix et Rhizoma] 3 g | 1) Severe cough: add Ephedra sinica Stapf [Ephedraceae; Ephedrae Herba], Prunus armeniaca L. [Rosaceae; Armeniacae Semen] 2) Aversion to cold: add Astragalus mongholicus Bunge [Fabaceae; Astragali Radix], Saposhnikovia divaricata (Turcz. ex Ledeb.) Schischk. [Apiaceae; Saposhnikoviae Radix] 3) Excessive phlegm and dampness, slimy fur: add Magnolia officinalis Rehder & E.H.Wilson [Magnoliaceae; Magnoliae Cortex] | NA | None | None | 1 month | None |
| Guan 2022 | Banxia Houpu-tang combined with Zhisou-san | Decoction | Platycodon grandiflorus (Jacq.) A.DC. [Campanulaceae; Platycodonis Radix] 15 g, Aster tataricus L.f. [Asteraceae; Asteris Radix et Rhizoma] 15 g, Magnolia officinalis Rehder & E.H.Wilson [Magnoliaceae; Magnoliae Cortex] 15 g, Stemona tuberosa Lour. [Stemonaceae; Stemonae Radix] 15 g, Pinellia ternata (Thunb.) Makino [Araceae; Pinelliae Tuber] 10 g, Perilla frutescens (L.) Britton [Lamiaceae; Perillae Folium] 10 g, Sesamum indicum L. [Pedaliaceae; Schizonepetae Spica] 10 g, Citrus × aurantium f. deliciosa (Ten.) M.Hiroe [Rutaceae; Citri Unshius Pericarpium] 10 g, Vincetoxicum stauntonii (Decne.) C.Y.Wu & D.Z.Li [Apocynaceae; Cynanchi Stauntonii Rhizoma Et Radix] 10 g, Glycyrrhiza glabra L. [Fabaceae; Glycyrrhizae Radix et Rhizoma] 5 g | NA | NA | None | None | 2 weeks | 6 months |
| Han 2008 | Manhai-yin | Decoction | Astragalus mongholicus Bunge [Fabaceae; Astragali Radix] 20 g, Platycodon grandiflorus (Jacq.) A.DC. [Campanulaceae; Platycodonis Radix] 10 g, Atractylodes lancea (Thunb.) DC. [Asteraceae; Atractylodis Rhizoma] 10 g, Saposhnikovia divaricata (Turcz. ex Ledeb.) Schischk. [Apiaceae; Saposhnikoviae Radix] 10 g, Stemona tuberosa Lour. [Stemonaceae; Stemonae Radix] 10 g, Citrus × aurantium f. deliciosa (Ten.) M.Hiroe [Rutaceae; Citri Unshius Pericarpium] 10 g, Prunus armeniaca L. [Rosaceae; Armeniacae Semen] 10 g, Forsythia suspensa (Thunb.) Vahl [Oleaceae; Forsythiae Fructus] 15 g, Paeonia lactiflora Pall. [Paeoniaceae; Radix Paeoniae Rubra] 10 g, Schisandra chinensis (Turcz.) Baill. [Schisandraceae; Schisandrae Fructus] 10 g, Cryptotympana dubia (Haupt) [Cicadidae; Cicadidae Periostracum] 5 g, Galanthus nivalis L. [Amaryllidaceae; Lumbricus] 10 g, Mentha canadensis L. [Lamiaceae; Menthae Herba] 5 g, Glycyrrhiza glabra L. [Fabaceae; Glycyrrhizae Radix et Rhizoma] 5 g | 1) Obvious external symptom: add Sesamum indicum L. [Pedaliaceae; Schizonepetae Spica], Perilla frutescens (L.) Britton [Lamiaceae; Perillae Folium] 2) Obvious phlegm and heat: add Trichosanthes kirilowii Maxim. [Cucurbitaceae; Trichosanthis Semen], Rhaphiolepis bibas (Lour.) Galasso & Banfi [Rosaceae; Eriobotryae Folium] 3) Yin deficinecy: add Adenophora triphylla (Thunb.) A.DC. [Campanulaceae; Adenophorae Radix], Polygonatum odoratum (Mill.) Druce [Asparagaceae; Polygonati Odorati Rhizoma] | NA | None | None | 2 weeks | None |
| He 2021 | Bufei Zhike-tang | Decoction | Glehnia littoralis (A.Gray) F.Schmidt ex Miq. [Apiaceae; Glehniae Radix] 9 g, Ophiopogon japonicus (Thunb.) Ker Gawl. [Asparagaceae; Liriopis seu Ophiopogonis Tuber] 9 g, Rehmannia glutinosa (Gaertner) Liboschitz ex Steudel [Scrophulariaceae; Rehmanniae Radix Recens] 9 g, Scrophularia ningpoensis Hemsl. [Scrophulariaceae; Scrophulariae Radix] 6 g, Fritillaria cirrhosa D.Don [Liliaceae; Fritillariae Cirrhosae Bulbus] 9 g, Trichosanthes kirilowii Maxim. [Cucurbitaceae; Trichosanthis Radix] 9 g, Polygonatum odoratum (Mill.) Druce [Asparagaceae; Polygonati Odorati Rhizoma] 9 g, Morus alba L. [Moraceae; Mori Folium] 9 g, Anemarrhena asphodeloides Bunge [Asparagaceae; Anemarrhenae Rhizoma] 6 g, Platycodon grandiflorus (Jacq.) A.DC. [Campanulaceae; Platycodonis Radix] 6 g, Panax ginseng C.A.Mey. [Araliaceae; Ginseng Radix] 9 g, Ziziphus jujuba Mill. [Rhamnaceae; Zizyphi Fructus] 6 pieces, Glycyrrhiza glabra L. [Fabaceae; Glycyrrhizae Radix et Rhizoma] 6 g | 1) Heat in the palms and soles: add Phellodendron amurense Rupr. [Rutaceae; Phellodendri Cortex] 6 g, Ligustrum lucidum W.T.Aiton [Oleaceae; Ligustri Fructus] 9 g, Schisandra chinensis (Turcz.) Baill. [Schisandraceae; Schisandrae Fructus] 6 g 2) Night sweating: add Prunus mume (Siebold) Siebold & Zucc. [Rosaceae; Mume Fructus] 9 g, Ostrea gigas Thunberg [Ostreidae; Ostreae Testa] 15 g, Triticum aestivum L. [Poaceae; Tritici Fructus Levis] 15 g 3) Bloody sputum: add Paeonia × suffruticosa Andrews [Paeoniaceae; Moutan Radicis Cortex] 9 g, Agrimonia pilosa Ledeb. [Rosaceae; Herba Agrimoniae] 15 g, Nelumbo nucifera Gaertn. [Nelumbonaceae; Nodus Nelumbinis Rhizomatis] 9 g 4) Tidal fever: add Artemisia annua L. [Asteraceae; Artemisiae Annuae Herba] 9 g, Pelodiscus sinensis (Wiegmann) [Trionychidae; Pelodiscis Carapax] 9 g, Coptis chinensis Franch. [Ranunculaceae; Coptidis Rhizoma] 9 g 5) Shortness of breath: Schisandra chinensis (Turcz.) Baill. [Schisandraceae; Schisandrae Fructus] 6 g, Terminalia chebula Retz. [Combretaceae; Terminaliae Fructus] 9 g | NA | None | None | 2 weeks | None |
| Huang 2021 | Zhisou-san | Decoction | Sesamum indicum L. [Pedaliaceae; Schizonepetae Spica] 12 g, Aster tataricus L.f. [Asteraceae; Asteris Radix et Rhizoma] 12 g, Platycodon grandiflorus (Jacq.) A.DC. [Campanulaceae; Platycodonis Radix] 12 g, Stemona tuberosa Lour. [Stemonaceae; Stemonae Radix] 12 g, Vincetoxicum stauntonii (Decne.) C.Y.Wu & D.Z.Li [Apocynaceae; Cynanchi Stauntonii Rhizoma Et Radix] 12 g, Citrus × aurantium f. deliciosa (Ten.) M.Hiroe [Rutaceae; Citri Unshius Pericarpium] 6 g, Glycyrrhiza glabra L. [Fabaceae; Glycyrrhizae Radix et Rhizoma] 4 g | 1) wind-cold: add Ephedra sinica Stapf [Ephedraceae; Ephedrae Herba] 6 g, Prunus armeniaca L. [Rosaceae; Armeniacae Semen] 12 g, Kitagawia praeruptora (Dunn) Pimenov [Apiaceae; Peucedani Radix] 12 g 2) wind-heat: add Morus alba L. [Moraceae; Mori Folium] 12 g, Chrysanthemum × morifolium (Ramat.) Hemsl. [Asteraceae; Chrysanthmi Flos] 12 g, Mentha canadensis L. [Lamiaceae; Menthae Herba] 12 g, Arctium lappa L. [Asteraceae; Arctii Fructus] 12 g 3) Wind-dryness hurts the lung: add Morus alba L. [Moraceae; Mori Folium] 12 g, Mentha canadensis L. [Lamiaceae; Menthae Herba] 12 g, Adenophora triphylla (Thunb.) A.DC. [Campanulaceae; Adenophorae Radix] 9 g, Trichosanthes kirilowii Maxim. [Cucurbitaceae; Trichosanthis Radix] 9 g, Phragmites australis (Cav.) Trin. ex Steud. [Poaceae; Phragmitis Rhizoma] 9 g 4) Phlegm-dampness: add Pinellia ternata (Thunb.) Makino [Araceae; Pinelliae Tuber] 12 g, Poria cocos Wolf [Polyporaceae; Poria Sclerotium] 12 g, Atractylodes lancea (Thunb.) DC. [Asteraceae; Atractylodis Rhizoma] 12 g, Tussilago farfara L. [Asteraceae; Farfarae Flos] 12 g 5) Phlegm-heat stagnation in the lung: add Scutellaria baicalensis Georgi [Lamiaceae; Scutellariae Radix] 12 g, Gardenia jasminoides J.Ellis [Rubiaceae; Gardeniae Fructus] 12 g, Anemarrhena asphodeloides Bunge [Asparagaceae; Anemarrhenae Rhizoma] 12 g, Morus alba L. [Moraceae; Mori Radicis Cortex] 12 g 6) Liver fire invading the lung: add Morus alba L. [Moraceae; Mori Radicis Cortex] 12 g, Lycium barbarum L. [Solanaceae; Lycii Radicis Cortex] 12 g, Gardenia jasminoides J.Ellis [Rubiaceae; Gardeniae Fructus] 12 g, Paeonia × suffruticosa Andrews [Paeoniaceae; Moutan Radicis Cortex] 12 g 7) Lung yin depletion: add Adenophora triphylla (Thunb.) A.DC. [Campanulaceae; Adenophorae Radix] 12 g, Ophiopogon japonicus (Thunb.) Ker Gawl. [Asparagaceae; Liriopis seu Ophiopogonis Tuber] 12 g, Trichosanthes kirilowii Maxim. [Cucurbitaceae; Trichosanthis Radix] 12 g | NA | None | None | 4 weeks | None |
| Hui 2020 | 1) Phlegm-dampness accumulation in the lung: Pingwei Erchen-tang combined with Sanzi Yangqin-tang 2) Phlegm-heat stagnation in the lung: Qingjin Huatan-tang 3) Liver fire invading the lung: Xiebai-san combined with Daiha-san 4) Lung yin depletion: Shashen Maidong-tang | Decoction | 1) Phlegm-dampness accumulation in the lung: Raphanus raphanistrum subsp. sativus (L.) Domin [Brassicaceae; Raphani Semen] 15 g, Sinapis alba L. [Brassicaceae; Sinapis Semen Alba] 15 g, Perilla frutescens (L.) Britton [Lamiaceae; Perillae Fructus] 15 g, Glycyrrhiza glabra L. [Fabaceae; Glycyrrhizae Radix et Rhizoma] 10 g, Prunus mume (Siebold) Siebold & Zucc. [Rosaceae; Mume Fructus] 10 g, Citrus × aurantium f. deliciosa (Ten.) M.Hiroe [Rutaceae; Citri Unshius Pericarpium] 15 g, Poria cocos Wolf [Polyporaceae; Poria Sclerotium] 15 g, Pinellia ternata (Thunb.) Makino [Araceae; Pinelliae Tuber] 9 g, Zingiber officinale Roscoe [Zingiberaceae; Zingiberis Rhizoma] 10 g 2) Phlegm-heat stagnation in the lung: Scutellaria baicalensis Georgi [Lamiaceae; Scutellariae Radix] 12 g, Gardenia jasminoides J.Ellis [Rubiaceae; Gardeniae Fructus] 12 g, Morus alba L. [Moraceae; Mori Radicis Cortex] 12 g, Trichosanthes kirilowii Maxim. [Cucurbitaceae; Trichosanthis Semen] 12 g, Anemarrhena asphodeloides Bunge [Asparagaceae; Anemarrhenae Rhizoma] 10 g, Citrus × aurantium f. deliciosa (Ten.) M.Hiroe [Rutaceae; Citri Unshius Pericarpium] 10 g, Fritillaria cirrhosa D.Don [Liliaceae; Fritillariae Cirrhosae Bulbus] 9 g, Ophiopogon japonicus (Thunb.) Ker Gawl. [Asparagaceae; Liriopis seu Ophiopogonis Tuber] 6 g, Poria cocos Wolf [Polyporaceae; Poria Sclerotium] 6 g, Platycodon grandiflorus (Jacq.) A.DC. [Campanulaceae; Platycodonis Radix] 6 g, Glycyrrhiza glabra L. [Fabaceae; Glycyrrhizae Radix et Rhizoma] 3 g 3) Liver fire invading the lung: Persicaria tinctoria (Aiton) Spach [Polygonaceae; Indigo Pulverata Levis] 12 g, Morus alba L. [Moraceae; Mori Radicis Cortex] 12 g, Lycium barbarum L. [Solanaceae; Lycii Radicis Cortex] 10 g, Meretrix meretrix Linné [Veneridae; Meretricis Concha] 10 g, Scutellaria baicalensis Georgi [Lamiaceae; Scutellariae Radix] 10 g, Trichosanthes kirilowii Maxim. [Cucurbitaceae; Trichosanthis Radix] 10 g, Glycyrrhiza glabra L. [Fabaceae; Glycyrrhizae Radix et Rhizoma] 6 g 4) Lung yin depletion: Adenophora triphylla (Thunb.) A.DC. [Campanulaceae; Adenophorae Radix] 20 g, Ophiopogon japonicus (Thunb.) Ker Gawl. [Asparagaceae; Liriopis seu Ophiopogonis Tuber] 20 g, Polygonatum odoratum (Mill.) Druce [Asparagaceae; Polygonati Odorati Rhizoma] 10 g, Lablab purpureus subsp. purpureus [Fabaceae; Dolichoris Semen] 10 g, Morus alba L. [Moraceae; Mori Folium] 15 g, Trichosanthes kirilowii Maxim. [Cucurbitaceae; Trichosanthis Radix] 15 g, Glycyrrhiza glabra L. [Fabaceae; Glycyrrhizae Radix et Rhizoma] 5 g | NA | NA | None | None | 10 days | 6 months |
| Ji 2022 | Mashe Donghua-tang | Decoction | Ephedra sinica Stapf [Ephedraceae; Ephedrae Herba] 9 g, Iris domestica (L.) Goldblatt & Mabb. [Iridaceae; Belamcandae Rhizoma] 9 g, Tussilago farfara L. [Asteraceae; Farfarae Flos] 15 g, Prunus armeniaca L. [Rosaceae; Armeniacae Semen ]10 g, Fritillaria thunbergii Miq. [Liliaceae; Fritillariae Thunbergii Bulbus] 12 g, Kitagawia praeruptora (Dunn) Pimenov [Apiaceae; Peucedani Radix] 15 g, Aster tataricus L.f. [Asteraceae; Asteris Radix et Rhizoma] 10 g, Rhaphiolepis bibas (Lour.) Galasso & Banfi [Rosaceae; Eriobotryae Folium] 15 g, Platycodon grandiflorus (Jacq.) A.DC. [Campanulaceae; Platycodonis Radix] 10 g, Arctium lappa L. [Asteraceae; Arctii Fructus] 15 g, Scutellaria baicalensis Georgi [Lamiaceae; Scutellariae Radix] 6 g, Glycyrrhiza glabra L. [Fabaceae; Glycyrrhizae Radix et Rhizoma] 6 g, Stemona tuberosa Lour. [Stemonaceae; Stemonae Radix] 15 g, Perilla frutescens (L.) Britton [Lamiaceae; Perillae Folium] 10 g, Houttuynia cordata Thunb. [Saururaceae; Houttuyniae Herba] 15 g, Bombyx mori (Linné) [Bombycidae; Batryticatus Bombyx] 12 g, Galanthus nivalis L. [Amaryllidaceae; Lumbricus] 15 g | 1) Chest distress, dry stool: add Trichosanthes kirilowii Maxim. [Cucurbitaceae; Trichosanthis Semen] 15 g, Gypsum Fibrosum 20 g 2) Dry throat, sore throat: remove Aster tataricus L.f. [Asteraceae; Asteris Radix et Rhizoma], Stemona tuberosa Lour. [Stemonaceae; Stemonae Radix], add Forsythia suspensa (Thunb.) Vahl [Oleaceae; Forsythiae Fructus] 12 g, Anemarrhena asphodeloides Bunge [Asparagaceae; Anemarrhenae Rhizoma] 15 g 3) Itchy throat and easy cough: add Cryptotympana dubia (Haupt) [Cicadidae; Cicadidae Periostracum] 6 g | NA | None | None | 2 weeks | None |
| Lai 2020 | Linggan Wuwei Jiangxin-tang combined with Zhisou-san | Decoction | Poria cocos Wolf [Polyporaceae; Poria Sclerotium] 18 g, Vincetoxicum stauntonii (Decne.) C.Y.Wu & D.Z.Li [Apocynaceae; Cynanchi Stauntonii Rhizoma Et Radix] 10 g, Glycyrrhiza glabra L. [Fabaceae; Glycyrrhizae Radix et Rhizoma] 9 g, Zingiber officinale Roscoe [Zingiberaceae; Zingiberis Rhizoma] 9 g, Aster tataricus L.f. [Asteraceae; Asteris Radix et Rhizoma] 6 g, Pinellia ternata (Thunb.) Makino [Araceae; Pinelliae Tuber] 6 g, Schisandra chinensis (Turcz.) Baill. [Schisandraceae; Schisandrae Fructus] 5 g, Asarum heterotropoides F.Schmidt [Aristolochiaceae; Asiasari Radix et Rhizoma] 3 g | 1) Aversion to cold: add Astragalus mongholicus Bunge [Fabaceae; Astragali Radix] 5 g, Saposhnikovia divaricata (Turcz. ex Ledeb.) Schischk. [Apiaceae; Saposhnikoviae Radix] 5 g 2) Severe cough: add Prunus armeniaca L. [Rosaceae; Armeniacae Semen] 5 g, Ephedra sinica Stapf [Ephedraceae; Ephedrae Herba] 5 g 3) Blood stasis and dampness, slimy fur: add Magnolia officinalis Rehder & E.H.Wilson [Magnoliaceae; Magnoliae Cortex] 5 g | NA | None | None | 1 month | None |
| Li 2018 | Zhisou-san | Decoction | Citrus × aurantium f. deliciosa (Ten.) M.Hiroe [Rutaceae; Citri Unshius Pericarpium] 3 g, Platycodon grandiflorus (Jacq.) A.DC. [Campanulaceae; Platycodonis Radix] 3 g, Kitagawia praeruptora (Dunn) Pimenov [Apiaceae; Peucedani Radix] 6 g, Glycyrrhiza glabra L. [Fabaceae; Glycyrrhizae Radix et Rhizoma] 6 g, Stemona tuberosa Lour. [Stemonaceae; Stemonae Radix] 8 g, Sesamum indicum L. [Pedaliaceae; Schizonepetae Spica] 3 g, Aster tataricus L.f. [Asteraceae; Asteris Radix et Rhizoma] 6 g | 1) Gastric cough: add Raphanus raphanistrum subsp. sativus (L.) Domin [Brassicaceae; Raphani Semen] 3-6 g, Crataegus monogyna Jacq. [Rosaceae; Crataegi Fructus] 9-12 g, Poria cocos Wolf [Polyporaceae; Poria Sclerotium] 6-9 g, Massa Medicata Fermentata 6-9 g -Obvious stomach fever: add Forsythia suspensa (Thunb.) Vahl [Oleaceae; Forsythiae Fructus] 3-6 g 2) Pharyngeal cough: add Ternate Grape Fern Herb 3-8 g, Adenophora triphylla (Thunb.) A.DC. [Campanulaceae; Adenophorae Radix] 3-6 g, Paris yunnanensis Franch. [Melanthiaceae; Rhizoma Paridis] 3-5 g, Forsythia suspensa (Thunb.) Vahl [Oleaceae; Forsythiae Fructus] 3-8 g, Arctium lappa L. [Asteraceae; Arctii Fructus] 3-6 g -Allergic symptoms: add Cryptotympana dubia (Haupt) [Cicadidae; Cicadidae Periostracum] 3 g -Significant nasal symptoms: add Magnolia officinalis Rehder & E.H.Wilson [Magnoliaceae; Magnoliae Flos] 3-5 g 3) Lung cough: add Fagopyrum cymosum (Trevir.) Meisn. [Polygonaceae; Fagopyrum dibotrys (D. Don) Hara] 6-8 g, Pinellia ternata (Thunb.) Makino [Araceae; Pinelliae Tuber] 3-5 g, Geranium carolinianum L. [Geraniaceae; Carolina Cranesbill] 6-8 g, Galanthus nivalis L. [Amaryllidaceae; Lumbricus] 3-5 g, Houttuynia cordata Thunb. [Saururaceae; Houttuyniae Herba] 6-8 g | NA | None | None | 2 weeks | 1 month |
| Li 2019 | Yangyin Qingfei-tang | Decoction | Rehmannia glutinosa (Gaertner) Liboschitz ex Steudel [Scrophulariaceae; Rehmanniae Radix Recens] 6 g, Ophiopogon japonicus (Thunb.) Ker Gawl. [Asparagaceae; Liriopis seu Ophiopogonis Tuber] 5 g, Paeonia lactiflora Pall. [Paeoniaceae; Paeoniae Radix] 5 g, Scrophularia ningpoensis Hemsl. [Scrophulariaceae; Scrophulariae Radix] 4 g, Paeonia × suffruticosa Andrews [Paeoniaceae; Moutan Radicis Cortex] 4 g, Fritillaria thunbergii Miq. [Liliaceae; Fritillariae Thunbergii Bulbus] 3 g, Mentha canadensis L. [Lamiaceae; Menthae Herba] 2 g, Glycyrrhiza glabra L. [Fabaceae; Glycyrrhizae Radix et Rhizoma] 10 g | NA | NA | None | None | 2 weeks | None |
| Li 2020 | Banxia Houpu-tang combined with Maimendong-tang | Decoction | Pinellia ternata (Thunb.) Makino [Araceae; Pinelliae Tuber] 10 g, Magnolia officinalis Rehder & E.H.Wilson [Magnoliaceae; Magnoliae Cortex] 15 g, Poria cocos Wolf [Polyporaceae; Poria Sclerotium] 20 g, Perilla frutescens (L.) Britton [Lamiaceae; Perillae Fructus] 10 g, Zingiber officinale Roscoe [Zingiberaceae; Zingiberis Rhizoma] 10 g, Ophiopogon japonicus (Thunb.) Ker Gawl. [Asparagaceae; Liriopis seu Ophiopogonis Tuber] 30 g, Codonopsis pilosula (Franch.) Nannf. [Campanulaceae; Codonopsis Pilosulae Radix] 20 g, Dioscorea oppositifolia L. [Dioscoreaceae; Dioscoreae Rhizoma] 20 g, Ziziphus jujuba Mill. [Rhamnaceae; Zizyphi Fructus] 15 g, Glycyrrhiza glabra L. [Fabaceae; Glycyrrhizae Radix et Rhizoma] 10 g | NA | NA | None | None | 12 weeks | 3 months |
| Li 2021 | Runfei Pingchuan-tang | Decoction | Codonopsis pilosula (Franch.) Nannf. [Campanulaceae; Codonopsis Pilosulae Radix] 20 g, Atractylodes lancea (Thunb.) DC. [Asteraceae; Atractylodis Rhizoma] 20 g, Poria cocos Wolf [Polyporaceae; Poria Sclerotium] 15 g, Citrus × aurantium f. deliciosa (Ten.) M.Hiroe [Rutaceae; Citri Unshius Pericarpium] 15 g, Dioscorea oppositifolia L. [Dioscoreaceae; Dioscoreae Rhizoma] 10 g, Coix lacryma-jobi var. ma-yuen (Rom.Caill.) Stapf [Poaceae; Coicis Semen] 10 g, Schisandra chinensis (Turcz.) Baill. [Schisandraceae; Schisandrae Fructus] 10 g, Pinellia ternata (Thunb.) Makino [Araceae; Pinelliae Tuber] 10 g, Glycyrrhiza glabra L. [Fabaceae; Glycyrrhizae Radix et Rhizoma] 6 g | 1) Cough, shortness of breath, phlegm and chest tightness: add Vincetoxicum stauntonii (Decne.) C.Y.Wu & D.Z.Li [Apocynaceae; Cynanchi Stauntonii Rhizoma Et Radix], Perilla frutescens (L.) Britton [Lamiaceae; Perillae Fructus] 2) Be afraid of the wind and cold: add Saposhnikovia divaricata (Turcz. ex Ledeb.) Schischk. [Apiaceae; Saposhnikoviae Radix] 3) Sweating: add Astragalus mongholicus Bunge [Fabaceae; Astragali Radix], Ziziphus jujuba Mill. [Rhamnaceae; Zizyphi Fructus] 4) Eat less: add Fructus Citri Sarcodactylis, Crataegus monogyna Jacq. [Rosaceae; Crataegi Fructus] 5) Excessive phlegm: add Kitagawia praeruptora (Dunn) Pimenov [Apiaceae; Peucedani Radix], Prunus armeniaca L. [Rosaceae; Armeniacae Semen] 6) Dry stool: add Raphanus raphanistrum subsp. sativus (L.) Domin [Brassicaceae; Raphani Semen], Citrus trifoliata L. [Rutaceae; Ponciri Fructus Immaturus] | NA | None | None | 2 weeks | **2 months** |
| Li 2022 | Yupingfeng oral liquid | Oral liquid | Astragalus mongholicus Bunge [Fabaceae; Astragali Radix], Atractylodes lancea (Thunb.) DC. [Asteraceae; Atractylodis Rhizoma], Saposhnikovia divaricata (Turcz. ex Ledeb.) Schischk. [Apiaceae; Saposhnikoviae Radix] | NA | Hubei Dongxin Pharmaceutical Co., Ltd. | None | None | 3 months | None |
| Liang 2022 | Suju oral liquid | Oral liquid | Perilla frutescens (L.) Britton [Lamiaceae; Perillae Fructus], Platycodon grandiflorus (Jacq.) A.DC. [Campanulaceae; Platycodonis Radix], Aster tataricus L.f. [Asteraceae; Asteris Radix et Rhizoma], Tussilago farfara L. [Asteraceae; Farfarae Flos], Pinellia ternata (Thunb.) Makino [Araceae; Pinelliae Tuber], Raphanus raphanistrum subsp. sativus (L.) Domin [Brassicaceae; Raphani Semen], Trichosanthes kirilowii Maxim. [Cucurbitaceae; Trichosanthis Semen], Kitagawia praeruptora (Dunn) Pimenov [Apiaceae; Peucedani Radix], Fritillaria thunbergii Miq. [Liliaceae; Fritillariae Thunbergii Bulbus], Descurainia sophia (L.) Webb ex Prantl [Brassicaceae; Lepidii seu Descurainiae Semen], Houttuynia cordata Thunb. [Saururaceae; Houttuyniae Herba], Glycyrrhiza glabra L. [Fabaceae; Glycyrrhizae Radix et Rhizoma] | NA | In-hospital preparations of Guangdong Second Hospital of Traditional Chinese Medicine | None | None | 2 weeks | None |
| Lin 2014 | Beiling-capsule | Capsule | Fritillaria cirrhosa D.Don [Liliaceae; Fritillariae Cirrhosae Bulbus], Gazella subgutturosa (Guldenstaedt) [Bovidae; Gazellae seu Saigae Cornu], Abelmoschus moschatus Medik. [Malvaceae; Moschus], Aquilaria malaccensis Lam. [Thymelaeaceae; Aquilariae Lignum], Realgar, Chloriti Lapis, Borax | NA | Shanghai Leiyunshang Pharmaceutical Co., Ltd. | None | None | 2 weeks | None |
| Liu 2012 | Zhisou-san | Decoction | Aster tataricus L.f. [Asteraceae; Asteris Radix et Rhizoma] 10 g, Stemona tuberosa Lour. [Stemonaceae; Stemonae Radix] 10 g, Tussilago farfara L. [Asteraceae; Farfarae Flos] 10 g, Vincetoxicum stauntonii (Decne.) C.Y.Wu & D.Z.Li [Apocynaceae; Cynanchi Stauntonii Rhizoma Et Radix] 10 g, Scutellaria baicalensis Georgi [Lamiaceae; Scutellariae Radix] 10 g, Galanthus nivalis L. [Amaryllidaceae; Lumbricus] 9 g, Atractylodes lancea (Thunb.) DC. [Asteraceae; Atractylodis Rhizoma] 6 g, Platycodon grandiflorus (Jacq.) A.DC. [Campanulaceae; Platycodonis Radix] 6 g, Glycyrrhiza glabra L. [Fabaceae; Glycyrrhizae Radix et Rhizoma] 6 g, Schisandra chinensis (Turcz.) Baill. [Schisandraceae; Schisandrae Fructus] 6 g | 1) Runny nose like water: add Zingiber officinale Roscoe [Zingiberaceae; Zingiberis Rhizoma] 2) Thick yellow sputum: add Fritillaria thunbergii Miq. [Liliaceae; Fritillariae Thunbergii Bulbus] | NA | None | None | 3 weeks | None |
| Liu 2020 | Linggan Wuwei Jiangxin-tang combined with Zhike-san | Decoction | Vincetoxicum stauntonii (Decne.) C.Y.Wu & D.Z.Li [Apocynaceae; Cynanchi Stauntonii Rhizoma Et Radix] 15 g, Schisandra chinensis (Turcz.) Baill. [Schisandraceae; Schisandrae Fructus] 10 g, Glycyrrhiza glabra L. [Fabaceae; Glycyrrhizae Radix et Rhizoma] 10 g, Platycodon grandiflorus (Jacq.) A.DC. [Campanulaceae; Platycodonis Radix] 10 g, Citrus × aurantium f. deliciosa (Ten.) M.Hiroe [Rutaceae; Citri Unshius Pericarpium] 10 g, Aster tataricus L.f. [Asteraceae; Asteris Radix et Rhizoma] 10 g, Poria cocos Wolf [Polyporaceae; Poria Sclerotium] 10 g, Pinellia ternata (Thunb.) Makino [Araceae; Pinelliae Tuber] 10 g, Zingiber officinale Roscoe [Zingiberaceae; Zingiberis Rhizoma] 10 g, Asarum heterotropoides F.Schmidt [Aristolochiaceae; Asiasari Radix et Rhizoma] 3 g | 1) Severe cough: add Prunus armeniaca L. [Rosaceae; Armeniacae Semen], Ephedra sinica Stapf [Ephedraceae; Ephedrae Herba] 2) Severe aversion to cold: add Saposhnikovia divaricata (Turcz. ex Ledeb.) Schischk. [Apiaceae; Saposhnikoviae Radix], Astragalus mongholicus Bunge [Fabaceae; Astragali Radix] | NA | None | None | 1 month | None |
| Liu 2022 | Chenxia Liujun-tang | Decoction | Coix lacryma-jobi var. ma-yuen (Rom.Caill.) Stapf [Poaceae; Coicis Semen] 20 g, Poria cocos Wolf [Polyporaceae; Poria Sclerotium] 15 g, Phragmites australis (Cav.) Trin. ex Steud. [Poaceae; Phragmitis Rhizoma] 15 g, Prunus persica (L.) Batsch [Rosaceae; Persicae Semen] 10 g, Aster tataricus L.f. [Asteraceae; Asteris Radix et Rhizoma] 10 g, Trichosanthes kirilowii Maxim. [Cucurbitaceae; Trichosanthis Pericarpium] 10 g, Pinellia ternata (Thunb.) Makino [Araceae; Pinelliae Tuber] 10 g, Benincasa hispida (Thunb.) Cogn. [Cucurbitaceae; Benincasae Semen] 10 g, Platycodon grandiflorus (Jacq.) A.DC. [Campanulaceae; Platycodonis Radix] 10 g, Prunus armeniaca L. [Rosaceae; Armeniacae Semen] 10 g, Trichosanthes kirilowii Maxim. [Cucurbitaceae; Trichosanthis Semen] 10 g, Massa Medicata Fermentata 10 g, Kitagawia praeruptora (Dunn) Pimenov [Apiaceae; Peucedani Radix] 10 g, Glycyrrhiza glabra L. [Fabaceae; Glycyrrhizae Radix et Rhizoma] 6 g, Citrus × aurantium f. deliciosa (Ten.) M.Hiroe [Rutaceae; Citri Unshius Pericarpium] 6 g, Ephedra sinica Stapf [Ephedraceae; Ephedrae Herba] 6 g | 1) Deficiency in the excess: add Cuscuta chinensis Lam. [Convolvulaceae; Cuscutae Semen] 10 g, Codonopsis pilosula (Franch.) Nannf. [Campanulaceae; Codonopsis Pilosulae Radix] 10 g 2) Excessive yellow phlegm: add Phyllostachys nigra Munro var. henonsis Stapf [Gramineae; Phyllostachyos Caulis in Taeniam] 10 g, Houttuynia cordata Thunb. [Saururaceae; Houttuyniae Herba] 15 g 3) Nocturnal CVA: add Galanthus nivalis L. [Amaryllidaceae; Lumbricus] 10 g, Iris domestica (L.) Goldblatt & Mabb. [Iridaceae; Belamcandae Rhizoma] 10 g | NA | None | None | 2 weeks | None |
| Lu 2013 | Banxia Xiexin-tang | Decoction | Pinellia ternata (Thunb.) Makino [Araceae; Pinelliae Tuber] 10 g, Zingiber officinale Roscoe [Zingiberaceae; Zingiberis Rhizoma] 10 g, Coptis chinensis Franch. [Ranunculaceae; Coptidis Rhizoma] 5 g, Scutellaria baicalensis Georgi [Lamiaceae; Scutellariae Radix] 10 g, Glycyrrhiza glabra L. [Fabaceae; Glycyrrhizae Radix et Rhizoma] 10 g, Adenophora triphylla (Thunb.) A.DC. [Campanulaceae; Adenophorae Radix] 30 g, Ziziphus jujuba Mill. [Rhamnaceae; Zizyphi Fructus] 10 g | 1) GERD: add Scapharca subcrenata (Lischke) [Arcidae; Scapharcae seu Tegillarcae Concha], Sepiella maindroni de Rochebrune [Sepiidae; Sepiae Endoconcha] 2) CVA: add Rhizoma Dioscoreae Nipponicae, Cryptotympana dubia (Haupt) [Cicadidae; Cicadidae Periostracum], Perilla frutescens (L.) Britton [Lamiaceae; Perillae Folium], Pheretima 3) UACS: add Xanthium strumarium L. [Asteraceae; Xanthii Fructus], Asarum heterotropoides F.Schmidt [Aristolochiaceae; Asiasari Radix et Rhizoma], Magnolia officinalis Rehder & E.H.Wilson [Magnoliaceae; Magnoliae Flos], Polistes mandarinus Saussure et Geer [Vespidae; Vespae Nidus], Angelica dahurica (Hoffm.) Benth. & Hook.f. ex Franch. & Sav. [Apiaceae; Angelicae Dahuricae Radix] 4) Spleen qi deficiency: add Astragalus mongholicus Bunge [Fabaceae; Astragali Radix], Codonopsis pilosula (Franch.) Nannf. [Campanulaceae; Codonopsis Pilosulae Radix], Atractylodes lancea (Thunb.) DC. [Asteraceae; Atractylodis Rhizoma], Pseudostellaria heterophylla (Miq.) Pax [Caryophyllaceae; Pseudostellariae Radix] 5) Severe yin deficiency: add Adenophora triphylla (Thunb.) A.DC. [Campanulaceae; Adenophorae Radix], Ophiopogon japonicus (Thunb.) Ker Gawl. [Asparagaceae; Liriopis seu Ophiopogonis Tuber], Rehmannia glutinosa (Gaertner) Liboschitz ex Steudel [Scrophulariaceae; Rehmanniae Radix Recens] 6) Severe cough: add Tussilago farfara L. [Asteraceae; Farfarae Flos], Kitagawia praeruptora (Dunn) Pimenov [Apiaceae; Peucedani Radix], Fritillaria cirrhosa D.Don [Liliaceae; Fritillariae Cirrhosae Bulbus], Prunus armeniaca L. [Rosaceae; Armeniacae Semen], Rhaphiolepis bibas (Lour.) Galasso & Banfi [Rosaceae; Eriobotryae Folium], Aster tataricus L.f. [Asteraceae; Asteris Radix et Rhizoma] 7) Phlegm dampness heavier: add Citrus × aurantium f. deliciosa (Ten.) M.Hiroe [Rutaceae; Citri Unshius Pericarpium], Atractylodes lancea (Thunb.) DC. [Asteraceae; Atractylodis Rhizoma] 8) Bowel qi blocked: add Rheum officinale Baill. [Polygonaceae; Rhei Radix et Rhizoma], Magnolia officinalis Rehder & E.H.Wilson [Magnoliaceae; Magnoliae Cortex], Citrus trifoliata L. [Rutaceae; Ponciri Fructus Immaturus] 9) Cough for a long time, the lungs are easy to dissipate: add Ginkgo biloba L. [Ginkgoaceae; Ginkgonis Semen] | NA | None | None | 2 weeks | None |
| Lu 2014 | Banxia Xiexin-tang | Decoction | Pinellia ternata (Thunb.) Makino [Araceae; Pinelliae Tuber] 10 g, Zingiber officinale Roscoe [Zingiberaceae; Zingiberis Rhizoma] 10 g, Coptis chinensis Franch. [Ranunculaceae; Coptidis Rhizoma] 5 g, Scutellaria baicalensis Georgi [Lamiaceae; Scutellariae Radix] 10 g, Glycyrrhiza glabra L. [Fabaceae; Glycyrrhizae Radix et Rhizoma] 10 g, Adenophora triphylla (Thunb.) A.DC. [Campanulaceae; Adenophorae Radix] 30 g, Ziziphus jujuba Mill. [Rhamnaceae; Zizyphi Fructus] 3 pieces | 1) CVA: add Cryptotympana dubia (Haupt) [Cicadidae; Cicadidae Periostracum], Galanthus nivalis L. [Amaryllidaceae; Lumbricus] 2) UACS: add Magnolia officinalis Rehder & E.H.Wilson [Magnoliaceae; Magnoliae Flos], Angelica dahurica (Hoffm.) Benth. & Hook.f. ex Franch. & Sav. [Apiaceae; Angelicae Dahuricae Radix], Asarum heterotropoides F.Schmidt [Aristolochiaceae; Asiasari Radix et Rhizoma] 3) GERD: add Scapharca subcrenata (Lischke) [Arcidae; Scapharcae seu Tegillarcae Concha], Sepiella maindroni de Rochebrune [Sepiidae; Sepiae Endoconcha] 4) Abdominal pain: add Cyperus rotundus L. [Cyperaceae; Cyperi Rhizoma] 5) Phlegm dampness: add Atractylodes lancea (Thunb.) DC. [Asteraceae; Atractylodis Rhizoma] 6) Dyspepsia: add Hordeum vulgare L. [Poaceae; Hordei Fructus Germinatus] | NA | None | None | 1 week | None |
| Luan 2021 | Tongqiao Zhike-tang | Granule | Coix lacryma-jobi var. ma-yuen (Rom.Caill.) Stapf [Poaceae; Coicis Semen] 4 g, Atractylodes lancea (Thunb.) DC. [Asteraceae; Atractylodis Rhizoma] 4 g, Scutellaria baicalensis Georgi [Lamiaceae; Scutellariae Radix] 4 g, Magnolia officinalis Rehder & E.H.Wilson [Magnoliaceae; Magnoliae Flos] 4 g, Acorus gramineus Aiton [Acoraceae; Acori Graminei Rhizoma] 4 g, Imperata cylindrica (L.) Raeusch. [Poaceae; Imperatae Rhizoma] 9 g, Phragmites australis (Cav.) Trin. ex Steud. [Poaceae; Phragmitis Rhizoma] 9 g, Ostrea gigas Thunberg [Ostreidae; Ostreae Testa] 9 g, Allium chinense G.Don [Amaryllidaceae; Allii Macrostemi Bulbus] 5 g, Curcuma longa L. [Zingiberaceae; Curcumae Radix] 5 g, Trichosanthes kirilowii Maxim. [Cucurbitaceae; Trichosanthis Semen] 5 g, Cucumis melo L. [Cucurbitaceae; Retinervus Luffae Fructus] 5 g, Fritillaria thunbergii Miq. [Liliaceae; Fritillariae Thunbergii Bulbus] 3 g, Glycyrrhiza glabra L. [Fabaceae; Glycyrrhizae Radix et Rhizoma] 2 g | NA | NA | None | None | 12 weeks | None |
| Lyu 2022 | Ojeok-san plus Saengmaek-san | Granule | Three times a day 1) Ojeok-san: Atractylodes lancea (Thunb.) DC. [Asteraceae; Atractylodis Rhizoma] 0.95 g, Ephedra sinica Stapf [Ephedraceae; Ephedrae Herba] 0.2 g, Citrus × aurantium f. deliciosa (Ten.) M.Hiroe [Rutaceae; Citri Unshius Pericarpium] 0.4 g, Magnolia officinalis Rehder & E.H.Wilson [Magnoliaceae; Magnoliae Cortex] 0.08 g, Platycodon grandiflorus (Jacq.) A.DC. [Campanulaceae; Platycodonis Radix] 0.43 g, Citrus × aurantium L. [Rutaceae; Aurantii Fructus Immaturus] 0.31 g, Angelica gigas Nakai [Apiaceae; Angelicae Gigantis Radix] 0.37, Zingiber officinale Roscoe [Zingiberaceae; Zingiberis Rhizoma] 0.22 g, Paeonia lactiflora Pall. [Paeoniaceae; Paeoniae Radix] 0.27 g, Poria cocos Wolf [Polyporaceae; Poria Sclerotium] 0.02 g, Conioselinum anthriscoides ‘Chuanxiong’ [Apiaceae; Cnidii Rhizoma] 0.3 g, Angelica dahurica (Hoffm.) Benth. & Hook.f. ex Franch. & Sav. [Apiaceae; Angelicae Dahuricae Radix] 0.31 g, Pinellia ternata (Thunb.) Makino [Araceae; Pinelliae Tuber] 0.22 g, Neolitsea cassia (L.) Kosterm. [Lauraceae; Cinnamomi Ramulus] 0.04 g, Glycyrrhiza glabra L. [Fabaceae; Glycyrrhizae Radix et Rhizoma] 0.03 g 2) Saengmaek-san: Ophiopogon japonicus (Thunb.) Ker Gawl. [Asparagaceae; Liriopis seu Ophiopogonis Tuber] 0.75 g, Panax ginseng C.A.Mey. [Araliaceae; Ginseng Radix] 0.30 g, Schisandra chinensis (Turcz.) Baill. [Schisandraceae; Schisandrae Fructus] 0.36 g | NA | Han Kook Shin Yak Pharm Co. Ltd. | None | None | 6 weeks | 8 weeks |
| Meng 2020 | Xuanfu Xiamaxiong Shaocao-tang | Decoction | Inula japonica Thunb. [Asteraceae; Inulae Flos] 9 g, Pinellia ternata (Thunb.) Makino [Araceae; Pinelliae Tuber] 9 g, Ephedra sinica Stapf [Ephedraceae; Ephedrae Herba] 6 g, Paeonia lactiflora Pall. [Paeoniaceae; Paeoniae Radix] 9 g, Conioselinum anthriscoides ‘Chuanxiong’ [Apiaceae; Cnidii Rhizoma] 9 g, Prunus armeniaca L. [Rosaceae; Armeniacae Semen] 9 g, Platycodon grandiflorus (Jacq.) A.DC. [Campanulaceae; Platycodonis Radix] 9 g, Houttuynia cordata Thunb. [Saururaceae; Houttuyniae Herba] 30 g, Glycyrrhiza glabra L. [Fabaceae; Glycyrrhizae Radix et Rhizoma] 6 g | 1) Sore throat: add Iris domestica (L.) Goldblatt & Mabb. [Iridaceae; Belamcandae Rhizoma] 6 g, Alkekengi officinarum var. franchetii (Mast.) R.J.Wang [Solanaceae; Physalis alkekengi var. franchetii] 9 g, Mentha canadensis L. [Lamiaceae; Menthae Herba] 3 g 2) Thick and less phlegm: add Fritillaria thunbergii Miq. [Liliaceae; Fritillariae Thunbergii Bulbus] 9 g, Morus alba L. [Moraceae; Mori Folium] 9 g 3) Aversion to wind, cold hands and feet: add Vincetoxicum mukdenense Kitag. [Apocynaceae; Radix Cynanchi Paniculati] 9 g, Sesamum indicum L. [Pedaliaceae; Schizonepetae Spica] 9 g | NA | None | None | 3 weeks | None |
| Niu 2020 | Zhisou-san | Decoction | Glycyrrhiza glabra L. [Fabaceae; Glycyrrhizae Radix et Rhizoma] 6 g, Prunus armeniaca L. [Rosaceae; Armeniacae Semen] 10 g, Ephedra sinica Stapf [Ephedraceae; Ephedrae Herba] 6 g, Platycodon grandiflorus (Jacq.) A.DC. [Campanulaceae; Platycodonis Radix] 10 g, Sesamum indicum L. [Pedaliaceae; Schizonepetae Spica] 6 g, Aster tataricus L.f. [Asteraceae; Asteris Radix et Rhizoma] 10 g, Vincetoxicum stauntonii (Decne.) C.Y.Wu & D.Z.Li [Apocynaceae; Cynanchi Stauntonii Rhizoma Et Radix] 10 g, Tussilago farfara L. [Asteraceae; Farfarae Flos] 10 g, Perilla frutescens (L.) Britton [Lamiaceae; Perillae Fructus] 10 g, Stemona tuberosa Lour. [Stemonaceae; Stemonae Radix] 10 g, Kitagawia praeruptora (Dunn) Pimenov [Apiaceae; Peucedani Radix] 10 g, Rhaphiolepis bibas (Lour.) Galasso & Banfi [Rosaceae; Eriobotryae Folium] 10 g | NA | NA | None | None | 1 week | None |
| Shen 2017 | Linggan Wuwei Jiangxin-tang | Decoction | Asarum heterotropoides F.Schmidt [Aristolochiaceae; Asiasari Radix et Rhizoma] 5 g, Schisandra chinensis (Turcz.) Baill. [Schisandraceae; Schisandrae Fructus] 5 g, Poria cocos Wolf [Polyporaceae; Poria Sclerotium] 12 g, Glycyrrhiza glabra L. [Fabaceae; Glycyrrhizae Radix et Rhizoma] 9 g, Zingiber officinale Roscoe [Zingiberaceae; Zingiberis Rhizoma] 9 g | 1) Phlegm dampness and slimy fur: add Magnolia officinalis Rehder & E.H.Wilson [Magnoliaceae; Magnoliae Cortex] 2) Aversion to cold: add Astragalus mongholicus Bunge [Fabaceae; Astragali Radix], Saposhnikovia divaricata (Turcz. ex Ledeb.) Schischk. [Apiaceae; Saposhnikoviae Radix] 3) Frequent cough: add Prunus armeniaca L. [Rosaceae; Armeniacae Semen], Ephedra sinica Stapf [Ephedraceae; Ephedrae Herba] | NA | None | None | 1 month | None |
| Shen 2019 | Xiaoer Xiaoji Zhike oral liquid | Oral liquid | Citrus trifoliata L. [Rutaceae; Ponciri Fructus Immaturus], Forsythia suspensa (Thunb.) Vahl [Oleaceae; Forsythiae Fructus], Crataegus monogyna Jacq. [Rosaceae; Crataegi Fructus], Cryptotympana dubia (Haupt) [Cicadidae; Cicadidae Periostracum], Platycodon grandiflorus (Jacq.) A.DC. [Campanulaceae; Platycodonis Radix], Areca catechu L. [Arecaceae; Arecae Semen], Trichosanthes kirilowii Maxim. [Cucurbitaceae; Trichosanthis Semen], Rhaphiolepis bibas (Lour.) Galasso & Banfi [Rosaceae; Eriobotryae Folium], Raphanus raphanistrum subsp. sativus (L.) Domin [Brassicaceae; Raphani Semen], Descurainia sophia (L.) Webb ex Prantl [Brassicaceae; Lepidii seu Descurainiae Semen] | NA | Lunan Houpu Pharmaceutical Co., Ltd. | None | None | 2 weeks | None |
| Shi 2010 | Pingjin-fang | Decoction | Stemona tuberosa Lour. [Stemonaceae; Stemonae Radix], Aster tataricus L.f. [Asteraceae; Asteris Radix et Rhizoma], Ephedra sinica Stapf [Ephedraceae; Ephedrae Herba], Prunus armeniaca L. [Rosaceae; Armeniacae Semen], Vincetoxicum stauntonii (Decne.) C.Y.Wu & D.Z.Li [Apocynaceae; Cynanchi Stauntonii Rhizoma Et Radix], Platycodon grandiflorus (Jacq.) A.DC. [Campanulaceae; Platycodonis Radix], Cryptotympana dubia (Haupt) [Cicadidae; Cicadidae Periostracum], Bombyx mori (Linné) [Bombycidae; Batryticatus Bombyx], Bupleurum falcatum L. [Apiaceae; Bupleuri Radix], Scutellaria baicalensis Georgi [Lamiaceae; Scutellariae Radix], Codonopsis pilosula (Franch.) Nannf. [Campanulaceae; Codonopsis Pilosulae Radix], Ophiopogon japonicus (Thunb.) Ker Gawl. [Asparagaceae; Liriopis seu Ophiopogonis Tuber], Glycyrrhiza glabra L. [Fabaceae; Glycyrrhizae Radix et Rhizoma] | NA | NA | None | None | 2 weeks | None |
| Song 2022 | Linggan Wuwei Jiangxin-tang combined with Zhisou-san | Decoction | Vincetoxicum stauntonii (Decne.) C.Y.Wu & D.Z.Li [Apocynaceae; Cynanchi Stauntonii Rhizoma Et Radix] 15 g, Poria cocos Wolf [Polyporaceae; Poria Sclerotium] 15 g, Citrus × aurantium f. deliciosa (Ten.) M.Hiroe [Rutaceae; Citri Unshius Pericarpium] 15 g, Schisandra chinensis (Turcz.) Baill. [Schisandraceae; Schisandrae Fructus] 10 g, Zingiber officinale Roscoe [Zingiberaceae; Zingiberis Rhizoma] 10 g, Aster tataricus L.f. [Asteraceae; Asteris Radix et Rhizoma] 10 g, Pinellia ternata (Thunb.) Makino [Araceae; Pinelliae Tuber] 10 g, Platycodon grandiflorus (Jacq.) A.DC. [Campanulaceae; Platycodonis Radix] 10 g, Glycyrrhiza glabra L. [Fabaceae; Glycyrrhizae Radix et Rhizoma] 10 g, Asarum heterotropoides F.Schmidt [Aristolochiaceae; Asiasari Radix et Rhizoma] 3 g, | 1) Aversion to cold: add Astragalus mongholicus Bunge [Fabaceae; Astragali Radix] 9 g, Saposhnikovia divaricata (Turcz. ex Ledeb.) Schischk. [Apiaceae; Saposhnikoviae Radix] 9 g 2) Severe cough: add Prunus armeniaca L. [Rosaceae; Armeniacae Semen] 6 g, Ephedra sinica Stapf [Ephedraceae; Ephedrae Herba] 6 g 3) Phlegm dampness and slimy fur: add Magnolia officinalis Rehder & E.H.Wilson [Magnoliaceae; Magnoliae Cortex] 9 g | NA | None | None | 1 month | None |
| Sun 2020 | Runzao Zhisou-tang | Decoction | Cryptotympana dubia (Haupt) [Cicadidae; Cicadidae Periostracum] 6 g, Adenophora triphylla (Thunb.) A.DC. [Campanulaceae; Adenophorae Radix] 9 g, Fritillaria thunbergii Miq. [Liliaceae; Fritillariae Thunbergii Bulbus] 6 g, The peel of rosaceous plant Pyrus bretschneideri, Nashi Pear or Pyrus ussuriensis Maxim etc [Pericarpium Pyri] 15 g, Prunus armeniaca L. [Rosaceae; Armeniacae Semen] 10 g, Platycodon grandiflorus (Jacq.) A.DC. [Campanulaceae; Platycodonis Radix] 9 g, Aster tataricus L.f. [Asteraceae; Asteris Radix et Rhizoma] 12 g, Morus alba L. [Moraceae; Mori Folium] 10 g, Mentha canadensis L. [Lamiaceae; Menthae Herba] 10 g, Ophiopogon japonicus (Thunb.) Ker Gawl. [Asparagaceae; Liriopis seu Ophiopogonis Tuber] 10 g, Stemona tuberosa Lour. [Stemonaceae; Stemonae Radix] 12 g, Glycyrrhiza glabra L. [Fabaceae; Glycyrrhizae Radix et Rhizoma] 10 g | 1) Severe fluid loss: add Polygonatum odoratum (Mill.) Druce [Asparagaceae; Polygonati Odorati Rhizoma] 2) Damaged lung collaterals, blood in phlegm: add Imperata cylindrica (L.) Raeusch. [Poaceae; Imperatae Rhizoma] 3) Thick sputum, difficult to cough up: add Trichosanthes kirilowii Maxim. [Cucurbitaceae; Trichosanthis Semen] | NA | None | None | 4 weeks | None |
| Tan 2020 | Xiaochaihu-tang | Decoction | Bupleurum falcatum L. [Apiaceae; Bupleuri Radix] 15 g, Pinellia ternata (Thunb.) Makino [Araceae; Pinelliae Tuber] 12 g, Panax ginseng C.A.Mey. [Araliaceae; Ginseng Radix] 10 g, Glycyrrhiza glabra L. [Fabaceae; Glycyrrhizae Radix et Rhizoma] 6 g, Scutellaria baicalensis Georgi [Lamiaceae; Scutellariae Radix] 6 g, Zingiber officinale Roscoe [Zingiberaceae; Zingiberis Rhizoma] 9 g, Magnolia officinalis Rehder & E.H.Wilson [Magnoliaceae; Magnoliae Cortex] 9 g, Poria cocos Wolf [Polyporaceae; Poria Sclerotium] 12 g, Prunus armeniaca L. [Rosaceae; Armeniacae Semen] 6 g | NA | NA | None | None | 1 week | None |
| Tang 2020 | 1) Wind pathogen subduing the lung: Sanao-tang combined with Zhisou-san 2) Lung and stomach qi counterflow: Xuanfu Daizhe-tang combined with Banxia Xiexin-tang 3) Liver fire invading the lung: Huangqin Xiebai-san combined with Danzhi Xiaoyao-wan 4) Phlegm dampness inner exuberance: Chenxia Liujunzi-tang 5) Lung and kidney yang deficiency: Xiaoqinglong-tang combined with Jinkui Shenqi-wan 6) Lung and kidney yin deficiency: Shashen Maidong-tang combined with Baihe Gujin-tang | Decoction | 1) Wind pathogen subduing the lung: Ephedra sinica Stapf [Ephedraceae; Ephedrae Herba] 10 g, Prunus armeniaca L. [Rosaceae; Armeniacae Semen] 15 g, Platycodon grandiflorus (Jacq.) A.DC. [Campanulaceae; Platycodonis Radix] 10 g, Sesamum indicum L. [Pedaliaceae; Schizonepetae Spica] 15 g, Aster tataricus L.f. [Asteraceae; Asteris Radix et Rhizoma] 15 g, Stemona tuberosa Lour. [Stemonaceae; Stemonae Radix] 15 g, Vincetoxicum stauntonii (Decne.) C.Y.Wu & D.Z.Li [Apocynaceae; Cynanchi Stauntonii Rhizoma Et Radix] 10 g, Citrus × aurantium f. deliciosa (Ten.) M.Hiroe [Rutaceae; Citri Unshius Pericarpium] 10 g, Glycyrrhiza glabra L. [Fabaceae; Glycyrrhizae Radix et Rhizoma] 5 g 2) Lung and stomach qi counterflow: Inula japonica Thunb. [Asteraceae; Inulae Flos] 10 g, Codonopsis pilosula (Franch.) Nannf. [Campanulaceae; Codonopsis Pilosulae Radix] 15 g, Zingiber officinale Roscoe [Zingiberaceae; Zingiberis Rhizoma] 3 pieces, Haematitum 10 g, Glycyrrhiza glabra L. [Fabaceae; Glycyrrhizae Radix et Rhizoma] 8 g, Pinellia ternata (Thunb.) Makino [Araceae; Pinelliae Tuber] 10 g, Ziziphus jujuba Mill. [Rhamnaceae; Zizyphi Fructus] 10 g, Coptis chinensis Franch. [Ranunculaceae; Coptidis Rhizoma] 3 g, Scutellaria baicalensis Georgi [Lamiaceae; Scutellariae Radix] 5 g, Zingiber officinale Roscoe [Zingiberaceae; Zingiberis Rhizoma] 5 g 3) Liver fire invading the lung: Scutellaria baicalensis Georgi [Lamiaceae; Scutellariae Radix] 10 g, Morus alba L. [Moraceae; Mori Radicis Cortex] 15 g, Lycium barbarum L. [Solanaceae; Lycii Radicis Cortex] 10 g, Glycyrrhiza glabra L. [Fabaceae; Glycyrrhizae Radix et Rhizoma] 5 g, Dioscorea oppositifolia L. [Dioscoreaceae; Dioscoreae Rhizoma] 15 g, Paeonia × suffruticosa Andrews [Paeoniaceae; Moutan Radicis Cortex] 10 g, Gardenia jasminoides J.Ellis [Rubiaceae; Gardeniae Fructus] 10 g, Angelica gigas Nakai [Apiaceae; Angelicae Gigantis Radix] 5 g, Paeonia lactiflora Pall. [Paeoniaceae; Paeoniae Radix] 10 g, Bupleurum falcatum L. [Apiaceae; Bupleuri Radix] 10 g, Poria cocos Wolf [Polyporaceae; Poria Sclerotium] 15 g, Atractylodes lancea (Thunb.) DC. [Asteraceae; Atractylodis Rhizoma] 15 g, Zingiber officinale Roscoe [Zingiberaceae; Zingiberis Rhizoma] 3 pieces 4) Phlegm dampness inner exuberance: Citrus × aurantium f. deliciosa (Ten.) M.Hiroe [Rutaceae; Citri Unshius Pericarpium] 15 g, Pinellia ternata (Thunb.) Makino [Araceae; Pinelliae Tuber] 15 g, Codonopsis pilosula (Franch.) Nannf. [Campanulaceae; Codonopsis Pilosulae Radix] 20 g, Atractylodes lancea (Thunb.) DC. [Asteraceae; Atractylodis Rhizoma] 15 g, Poria cocos Wolf [Polyporaceae; Poria Sclerotium] 15 g, Glycyrrhiza glabra L. [Fabaceae; Glycyrrhizae Radix et Rhizoma] 5 g, Atractylodes lancea (Thunb.) DC. [Asteraceae; Atractylodis Rhizoma] 10 g, Magnolia officinalis Rehder & E.H.Wilson [Magnoliaceae; Magnoliae Cortex] 10 g, Zingiber officinale Roscoe [Zingiberaceae; Zingiberis Rhizoma] 3 pieces, Prunus mume (Siebold) Siebold & Zucc. [Rosaceae; Mume Fructus] 10 g 5) Lung and kidney yang deficiency: Ephedra sinica Stapf [Ephedraceae; Ephedrae Herba] 10 g, Paeonia lactiflora Pall. [Paeoniaceae; Paeoniae Radix] 10 g, Schisandra chinensis (Turcz.) Baill. [Schisandraceae; Schisandrae Fructus] 5 g, Asarum heterotropoides F.Schmidt [Aristolochiaceae; Asiasari Radix et Rhizoma] 3 g, Zingiber officinale Roscoe [Zingiberaceae; Zingiberis Rhizoma] 5 g, Neolitsea cassia (L.) Kosterm. [Lauraceae; Cinnamomi Ramulus] 10 g, Glycyrrhiza glabra L. [Fabaceae; Glycyrrhizae Radix et Rhizoma] 5 g, Rehmannia glutinosa (Gaertn.) DC. [Orobanchaceae; Rehmanniae Radix Preparata] 15 g, Dioscorea oppositifolia L. [Dioscoreaceae; Dioscoreae Rhizoma] 15 g, Cornus officinalis Siebold & Zucc. [Cornaceae; Corni Fructus] 10 g, Alisma plantago-aquatica subsp. orientale (Sam.) Sam. [Alismataceae; Alismatis Rhizoma] 10 g, Paeonia × suffruticosa Andrews [Paeoniaceae; Moutan Radicis Cortex] 10 g, Poria cocos Wolf [Polyporaceae; Poria Sclerotium] 10 g, Aconitum carmichaelii Debeaux [Ranunculaceae; Aconiti Lateralis Radix Preparata] 10 g, Pinellia ternata (Thunb.) Makino [Araceae; Pinelliae Tuber] 10 g 6) Lung and kidney yin deficiency: Ophiopogon japonicus (Thunb.) Ker Gawl. [Asparagaceae; Liriopis seu Ophiopogonis Tuber] 20 g, Pinellia ternata (Thunb.) Makino [Araceae; Pinelliae Tuber] 5 g, Codonopsis pilosula (Franch.) Nannf. [Campanulaceae; Codonopsis Pilosulae Radix] 15 g, Glycyrrhiza glabra L. [Fabaceae; Glycyrrhizae Radix et Rhizoma] 5 g, Ziziphus jujuba Mill. [Rhamnaceae; Zizyphi Fructus] 10 g, Lilium lancifolium Thunb. [Liliaceae; Lilii Bulbus] 15 g, Rehmannia glutinosa (Gaertn.) DC. [Orobanchaceae; Rehmanniae Radix Preparata] 10 g, Rehmannia glutinosa (Gaertner) Liboschitz ex Steudel [Scrophulariaceae; Rehmanniae Radix Recens] 10 g, Angelica gigas Nakai [Apiaceae; Angelicae Gigantis Radix] 5 g, Paeonia lactiflora Pall. [Paeoniaceae; Paeoniae Radix] 10 g, Platycodon grandiflorus (Jacq.) A.DC. [Campanulaceae; Platycodonis Radix] 10 g, Scrophularia ningpoensis Hemsl. [Scrophulariaceae; Scrophulariae Radix] 10 g, Fritillaria cirrhosa D.Don [Liliaceae; Fritillariae Cirrhosae Bulbus] 5 g, Adenophora triphylla (Thunb.) A.DC. [Campanulaceae; Adenophorae Radix] 15 g | NA | NA | None | None | 6 weeks | None |
| Tian 2023 | Yifei Huatan-tang | Decoction | Sinapis alba L. [Brassicaceae; Sinapis Semen Alba] 10 g, Raphanus raphanistrum subsp. sativus (L.) Domin [Brassicaceae; Raphani Semen] 10 g, Perilla frutescens (L.) Britton [Lamiaceae; Perillae Fructus] 10 g, Descurainia sophia (L.) Webb ex Prantl [Brassicaceae; Lepidii seu Descurainiae Semen] 10 g, Atractylodes lancea (Thunb.) DC. [Asteraceae; Atractylodis Rhizoma] 10 g, Citrus × aurantium f. deliciosa (Ten.) M.Hiroe [Rutaceae; Citri Unshius Pericarpium] 10 g, Pinellia ternata (Thunb.) Makino [Araceae; Pinelliae Tuber] 10 g, Astragalus mongholicus Bunge [Fabaceae; Astragali Radix] 15 g, Schisandra chinensis (Turcz.) Baill. [Schisandraceae; Schisandrae Fructus] 6 g, Saposhnikovia divaricata (Turcz. ex Ledeb.) Schischk. [Apiaceae; Saposhnikoviae Radix] 6 g, Poria cocos Wolf [Polyporaceae; Poria Sclerotium] 12 g, Glycyrrhiza glabra L. [Fabaceae; Glycyrrhizae Radix et Rhizoma] 9 g, Prunus armeniaca L. [Rosaceae; Armeniacae Semen] 8 g, Magnolia officinalis Rehder & E.H.Wilson [Magnoliaceae; Magnoliae Cortex] 8 g | NA | NA | None | None | 2 weeks | None |
| Wang 2012 | Diaogan Hefei-tang | Decoction | Bupleurum falcatum L. [Apiaceae; Bupleuri Radix] 10 g, Paeonia lactiflora Pall. [Paeoniaceae; Paeoniae Radix] 15 g, Scutellaria baicalensis Georgi [Lamiaceae; Scutellariae Radix] 15 g, Pinellia ternata (Thunb.) Makino [Araceae; Pinelliae Tuber] 12 g, Citrus × aurantium f. deliciosa (Ten.) M.Hiroe [Rutaceae; Citri Unshius Pericarpium Immaturus] 10 g, Uncaria rhynchophylla (Miq.) Miq. [Rubiaceae; Uncariae Ramulus cum Uncus] 15 g, Adenophora triphylla (Thunb.) A.DC. [Campanulaceae; Adenophorae Radix] 30 g, Kitagawia praeruptora (Dunn) Pimenov [Apiaceae; Peucedani Radix] 12 g, Glycyrrhiza glabra L. [Fabaceae; Glycyrrhizae Radix et Rhizoma] 3 g, Platycodon grandiflorus (Jacq.) A.DC. [Campanulaceae; Platycodonis Radix] 12 g, Citrus × aurantium L. [Rutaceae; Aurantii Fructus Immaturus] 12 g, Citrus × aurantium f. deliciosa (Ten.) M.Hiroe [Rutaceae; Citri Unshius Pericarpium] 12 g, Aster tataricus L.f. [Asteraceae; Asteris Radix et Rhizoma] 15 g | NA | NA | None | None | 2 weeks | None |
| Wang 2016a | Linggan Wuwei Jiangxin-tang | Decoction | Vincetoxicum stauntonii (Decne.) C.Y.Wu & D.Z.Li [Apocynaceae; Cynanchi Stauntonii Rhizoma Et Radix] 20 g, Poria cocos Wolf [Polyporaceae; Poria Sclerotium] 15 g, Citrus × aurantium f. deliciosa (Ten.) M.Hiroe [Rutaceae; Citri Unshius Pericarpium] 15 g, Schisandra chinensis (Turcz.) Baill. [Schisandraceae; Schisandrae Fructus] 10 g, Zingiber officinale Roscoe [Zingiberaceae; Zingiberis Rhizoma] 10 g, Aster tataricus L.f. [Asteraceae; Asteris Radix et Rhizoma] 10 g, Platycodon grandiflorus (Jacq.) A.DC. [Campanulaceae; Platycodonis Radix] 10 g, Pinellia ternata (Thunb.) Makino [Araceae; Pinelliae Tuber] 10 g, Glycyrrhiza glabra L. [Fabaceae; Glycyrrhizae Radix et Rhizoma] 10 g, Asarum heterotropoides F.Schmidt [Aristolochiaceae; Asiasari Radix et Rhizoma] 3 g | 1) Severe sore throat: add Arctium lappa L. [Asteraceae; Arctii Fructus] 10 g 2) Cough like wheezing: add Prunus armeniaca L. [Rosaceae; Armeniacae Semen] 10 g 3) Aversion to wind and cold: add Saposhnikovia divaricata (Turcz. ex Ledeb.) Schischk. [Apiaceae; Saposhnikoviae Radix] 10 g | NA | None | None | 4 weeks | None |
| Wang 2016b | NR | Decoction | Bupleurum falcatum L. [Apiaceae; Bupleuri Radix] 10 g, Aster tataricus L.f. [Asteraceae; Asteris Radix et Rhizoma] 10 g, Morus alba L. [Moraceae; Mori Folium] 10 g, Morus alba L. [Moraceae; Mori Radicis Cortex] 10 g, Citrus × aurantium L. [Rutaceae; Aurantii Fructus Immaturus] 10 g, Trichosanthes kirilowii Maxim. [Cucurbitaceae; Trichosanthis Semen] 10 g, Galanthus nivalis L. [Amaryllidaceae; Lumbricus] 10 g, Prunus armeniaca L. [Rosaceae; Armeniacae Semen] 10 g, Platycodon grandiflorus (Jacq.) A.DC. [Campanulaceae; Platycodonis Radix] 6 g, Coix lacryma-jobi var. ma-yuen (Rom.Caill.) Stapf [Poaceae; Coicis Semen] 30 g | 1) Nausea and anorexia: add Rhaphiolepis bibas (Lour.) Galasso & Banfi [Rosaceae; Eriobotryae Folium] 10 g 2) Cough with dyspnea: add Inula japonica Thunb. [Asteraceae; Inulae Flos] 5 g 3) Sticky sputum: add Pumex 10 g, Stemona tuberosa Lour. [Stemonaceae; Stemonae Radix] 10 g | NA | None | None | 2 weeks | None |
| Wang 2019 | Qingre Xuanfei Qufeng Liyan-fang | Decoction | Fagopyrum dibotrys (D.Don)Hara [Polygonaceae; Fagopyrum dibotrys] 30 g, Coix lacryma-jobi var. ma-yuen (Rom.Caill.) Stapf [Poaceae; Coicis Semen] 30 g, Fritillaria thunbergii Miq. [Liliaceae; Fritillariae Thunbergii Bulbus] 20 g, Scutellaria baicalensis Georgi [Lamiaceae; Scutellariae Radix] 20 g, Usnea [Usneaceae; Usnea diffracta Vain.] 15 g, Calamitas Urinae Hominis 15 g, Daiha-san 15 g, Erodium stephanianum Willd. [Geraniaceae; Herba Erodiiherba Geranii] 15 g, Platycodon grandiflorus (Jacq.) A.DC. [Campanulaceae; Platycodonis Radix] 12 g, Morus alba L. [Moraceae; Mori Radicis Cortex] 12 g, Bambusa textilis McClure [Poaceae; Concretio Silicea Bambusae] 12 g, Perilla frutescens (L.) Britton [Lamiaceae; Caulis Perillae] 12 g, Pumex 12 g, Spirodela polyrhiza (L.) Schleid. [Araceae; Spirodelae Herba] 12 g, Bassia scoparia (L.) A.J.Scott [Amaranthaceae; Kochiae Fructus] 12 g, Oroxylum indicum (L.) Kurz [Bignoniaceae; Semen Oroxyli] 9 g, Gleditsia sinensis Lam. [Fabaceae; Gleditsiae Spina] 9 g | 1) Cough decreases but phlegm persists: add Prunus persica (L.) Batsch [Rosaceae; Persicae Semen] 15 g, Benincasa hispida (Thunb.) Cogn. [Cucurbitaceae; Benincasae Semen] 30 g, Phragmites australis (Cav.) Trin. ex Steud. [Poaceae; Phragmitis Rhizoma] 30 g 2) After the symptoms are relieved: add Astragalus mongholicus Bunge [Fabaceae; Astragali Radix] 15 g, Epimedium sagittatum (Siebold & Zucc.) Maxim. [Berberidaceae; Epimedii Herba] 20 g, Saposhnikovia divaricata (Turcz. ex Ledeb.) Schischk. [Apiaceae; Saposhnikoviae Radix] 12 g, Atractylodes lancea (Thunb.) DC. [Asteraceae; Atractylodis Rhizoma] 12 g | NA | None | None | 4 weeks | None |
| Wang 2020 | Bufei Zhike-tang | Decoction | Glehnia littoralis (A.Gray) F.Schmidt ex Miq. [Apiaceae; Glehniae Radix] 9 g, Ophiopogon japonicus (Thunb.) Ker Gawl. [Asparagaceae; Liriopis seu Ophiopogonis Tuber] 9 g, Rehmannia glutinosa (Gaertner) Liboschitz ex Steudel [Scrophulariaceae; Rehmanniae Radix Recens] 9 g, Scrophularia ningpoensis Hemsl. [Scrophulariaceae; Scrophulariae Radix] 9 g, Fritillaria cirrhosa D.Don [Liliaceae; Fritillariae Cirrhosae Bulbus] 9 g, Trichosanthes kirilowii Maxim. [Cucurbitaceae; Trichosanthis Radix] 9 g, Polygonatum odoratum (Mill.) Druce [Asparagaceae; Polygonati Odorati Rhizoma] 9 g, Morus alba L. [Moraceae; Mori Folium] 9 g, Anemarrhena asphodeloides Bunge [Asparagaceae; Anemarrhenae Rhizoma] 9 g, Platycodon grandiflorus (Jacq.) A.DC. [Campanulaceae; Platycodonis Radix] 6 g, Panax ginseng C.A.Mey. [Araliaceae; Ginseng Radix] 9 g, Ziziphus jujuba Mill. [Rhamnaceae; Zizyphi Fructus] 6 pieces, Glycyrrhiza glabra L. [Fabaceae; Glycyrrhizae Radix et Rhizoma] 6 g | 1) Blood in phlegm: add Paeonia × suffruticosa Andrews [Paeoniaceae; Moutan Radicis Cortex] 9 g, Agrimonia eupatoria L. [Rosaceae; Agrimoniae Herba] 15 g, Nelumbo nucifera Gaertn. [Nelumbonaceae; Nelumbinis Rhizomatis Nodus] 9 g 2) Tidal fever: add Artemisia annua L. [Asteraceae; Artemisiae Annuae Herba] 9 g, Pelodiscus sinensis (Wiegmann) [Trionychidae; Pelodiscis Carapax] 9 g, Coptis chinensis Franch. [Ranunculaceae; Coptidis Rhizoma] 9 g 3) Coughing and shortness of breath: add Schisandra chinensis (Turcz.) Baill. [Schisandraceae; Schisandrae Fructus] 6 g, Terminalia chebula Retz. [Combretaceae; Terminaliae Fructus] 9 g | NA | None | None | 2 weeks | None |
| Wang 2022 | Linggan Wuwei Jiangxin-tang combined with Zhisou-san | Decoction | Zingiber officinale Roscoe [Zingiberaceae; Zingiberis Rhizoma] 10 g, Citrus × aurantium f. deliciosa (Ten.) M.Hiroe [Rutaceae; Citri Unshius Pericarpium] 10 g, Pinellia ternata (Thunb.) Makino [Araceae; Pinelliae Tuber] 10 g, Glycyrrhiza glabra L. [Fabaceae; Glycyrrhizae Radix et Rhizoma] 10 g, Platycodon grandiflorus (Jacq.) A.DC. [Campanulaceae; Platycodonis Radix] 10 g, Poria cocos Wolf [Polyporaceae; Poria Sclerotium] 10 g, Aster tataricus L.f. [Asteraceae; Asteris Radix et Rhizoma] 10 g, Vincetoxicum stauntonii (Decne.) C.Y.Wu & D.Z.Li [Apocynaceae; Cynanchi Stauntonii Rhizoma Et Radix] 15 g, Asarum heterotropoides F.Schmidt [Aristolochiaceae; Asiasari Radix et Rhizoma] 3 g | 1) Sever cough: add Ephedra sinica Stapf [Ephedraceae; Ephedrae Herba] 8 g, Prunus armeniaca L. [Rosaceae; Armeniacae Semen] 8 g 2) Aversion to cold: add Saposhnikovia divaricata (Turcz. ex Ledeb.) Schischk. [Apiaceae; Saposhnikoviae Radix] 8 g, Astragalus mongholicus Bunge [Fabaceae; Astragali Radix] 8 g 3) Phlegm dampness and slimy fur: add Magnolia officinalis Rehder & E.H.Wilson [Magnoliaceae; Magnoliae Cortex] 8 g | NA | None | None | 1 month | None |
| Wu 2019 | Zhike-tablets | Tablet | Aster tataricus L.f. [Asteraceae; Asteris Radix et Rhizoma], Platycodon grandiflorus (Jacq.) A.DC. [Campanulaceae; Platycodonis Radix], Stemona tuberosa Lour. [Stemonaceae; Stemonae Radix], Kitagawia praeruptora (Dunn) Pimenov [Apiaceae; Peucedani Radix], Citrus × aurantium f. deliciosa (Ten.) M.Hiroe [Rutaceae; Citri Unshius Pericarpium], Agrimonia pilosa Ledeb. [Rosaceae; Herba Agrimoniae], Platycladus orientalis (L.) Franco [Cupressaceae; Thujae Orientalis Folium], Sesamum indicum L. [Pedaliaceae; Schizonepetae Spica], Glycyrrhiza glabra L. [Fabaceae; Glycyrrhizae Radix et Rhizoma] | NA | NA | None | None | 4 weeks | None |
| Wu 2020 | NR | Decoction | Rhaphiolepis bibas (Lour.) Galasso & Banfi [Rosaceae; Eriobotryae Folium] 10 g, Platycodon grandiflorus (Jacq.) A.DC. [Campanulaceae; Platycodonis Radix] 10 g, Schisandra chinensis (Turcz.) Baill. [Schisandraceae; Schisandrae Fructus] 10 g, Actaea racemosa L. [Ranunculaceae; Cimicifugae Rhizoma] 10 g, Inula japonica Thunb. [Asteraceae; Inulae Flos] 10 g, Raphanus raphanistrum subsp. sativus (L.) Domin [Brassicaceae; Raphani Semen] 10 g, Achyranthes bidentata Blume [Amaranthaceae; Achyranthis Radix] 10 g, Alisma plantago-aquatica subsp. orientale (Sam.) Sam. [Alismataceae; Alismatis Rhizoma] 10 g, Prunus armeniaca L. [Rosaceae; Armeniacae Semen] 7 g, Haematitum 20 g, Magenetitum 20 g, Qing Long Chi 20 g | 1) Gastroesophageal reflux: add Sepiella maindroni de Rochebrune [Sepiidae; Sepiae Endoconcha] 2) Heat in the upper energizer: add Oroxylum indicum (L.) Kurz [Bignoniaceae; Semen Oroxyli], Scutellaria baicalensis Georgi [Lamiaceae; Scutellariae Radix] 3) Yin deficiency: add Ophiopogon japonicus (Thunb.) Ker Gawl. [Asparagaceae; Liriopis seu Ophiopogonis Tuber] 4) Severe cough: add Tussilago farfara L. [Asteraceae; Farfarae Flos], Kitagawia praeruptora (Dunn) Pimenov [Apiaceae; Peucedani Radix] 5) Mixed with phlegm and dampness: add Atractylodes lancea (Thunb.) DC. [Asteraceae; Atractylodis Rhizoma], Citrus × aurantium f. deliciosa (Ten.) M.Hiroe [Rutaceae; Citri Unshius Pericarpium] 6) Hard to sleep: add Poria cocos Wolf [Polyporaceae; Poria Sclerotium], Poria cocos Wolf [Polyporaceae; Poria Sclertum Cum Pini Radix], Reynoutria multiflora (Thunb.) Moldenke [Polygonaceae; Polygoni Multiflori Caulis] | NA | None | None | 2 weeks | None |
| Xia 2021 | Xuanfei Zhisou-tang | Decoction | Scrophularia ningpoensis Hemsl. [Scrophulariaceae; Scrophulariae Radix] 15 g, Prunus armeniaca L. [Rosaceae; Armeniacae Semen] 10 g, Ephedra sinica Stapf [Ephedraceae; Ephedrae Herba] 10 g, Platycodon grandiflorus (Jacq.) A.DC. [Campanulaceae; Platycodonis Radix] 10 g, Aster tataricus L.f. [Asteraceae; Asteris Radix et Rhizoma] 10 g, Sesamum indicum L. [Pedaliaceae; Schizonepetae Spica] 10 g, Tussilago farfara L. [Asteraceae; Farfarae Flos] 10 g, Cryptotympana dubia (Haupt) [Cicadidae; Cicadidae Periostracum] 5 g, Perilla frutescens (L.) Britton [Lamiaceae; Perillae Fructus] 15 g, Kitagawia praeruptora (Dunn) Pimenov [Apiaceae; Peucedani Radix] 10 g, Schisandra chinensis (Turcz.) Baill. [Schisandraceae; Schisandrae Fructus] 5 g, Arctium lappa L. [Asteraceae; Arctii Fructus] 15 g, Glycyrrhiza glabra L. [Fabaceae; Glycyrrhizae Radix et Rhizoma] 10 g | NA | NA | None | None | 2 weeks | None |
| Xie 2012 | Shashen Maidong-tang | Decoction | Adenophora triphylla (Thunb.) A.DC. [Campanulaceae; Adenophorae Radix] 20 g, Ophiopogon japonicus (Thunb.) Ker Gawl. [Asparagaceae; Liriopis seu Ophiopogonis Tuber] 20 g, Polygonatum odoratum (Mill.) Druce [Asparagaceae; Polygonati Odorati Rhizoma] 10 g, Morus alba L. [Moraceae; Mori Folium] 15 g, Trichosanthes kirilowii Maxim. [Cucurbitaceae; Trichosanthis Radix] 15 g, Lablab purpureus subsp. purpureus [Fabaceae; Dolichoris Semen] 10 g, Glycyrrhiza glabra L. [Fabaceae; Glycyrrhizae Radix et Rhizoma] 5 g | 1) Shortness of breath worsened by cough: add Schisandra chinensis (Turcz.) Baill. [Schisandraceae; Schisandrae Fructus] 2) Tidal fever: add Folium Mahonae, Stellariae seu Gypsophilae Radix, Lycium barbarum L. [Solanaceae; Lycii Radicis Cortex] 3) Night sweating: add Prunus mume (Siebold) Siebold & Zucc. [Rosaceae; Mume Fructus], Triticum aestivum L. [Poaceae; Tritici Fructus Levis] 4) Spitting yellow phlegm: add Meretrix meretrix Linné [Veneridae; Meretricis Concha], Anemarrhena asphodeloides Bunge [Asparagaceae; Anemarrhenae Rhizoma], Scutellaria baicalensis Georgi [Lamiaceae; Scutellariae Radix] 5) Bloody sputum: add Paeonia × suffruticosa Andrews [Paeoniaceae; Moutan Radicis Cortex], Gardenia jasminoides J.Ellis [Rubiaceae; Gardeniae Fructus], Nelumbo nucifera Gaertn. [Nelumbonaceae; Nelumbinis Rhizomatis Nodus] | NA | None | None | 2 weeks | None |
| Xie 2017 | Zhike No.1 granule | Granule | Morus alba L. [Moraceae; Mori Radicis Cortex] 10 g, Prunus armeniaca L. [Rosaceae; Armeniacae Semen] 10 g, Scutellaria baicalensis Georgi [Lamiaceae; Scutellariae Radix] 10 g, Taraxacum sect. Taraxacum F.H.Wigg. [Asteraceae; Taraxaci Herba] 10 g, Rhaphiolepis bibas (Lour.) Galasso & Banfi [Rosaceae; Eriobotryae Folium] 10 g, Stemona tuberosa Lour. [Stemonaceae; Stemonae Radix] 10 g, Citrus × aurantium f. deliciosa (Ten.) M.Hiroe [Rutaceae; Citri Unshius Pericarpium] 10 g, Iris domestica (L.) Goldblatt & Mabb. [Iridaceae; Belamcandae Rhizoma] 10 g, Platycodon grandiflorus (Jacq.) A.DC. [Campanulaceae; Platycodonis Radix] 10 g, Citrus × aurantium L. [Rutaceae; Aurantii Fructus Immaturus] 10 g, Trichosanthes kirilowii Maxim. [Cucurbitaceae; Trichosanthis Semen] 10 g, Vincetoxicum stauntonii (Decne.) C.Y.Wu & D.Z.Li [Apocynaceae; Cynanchi Stauntonii Rhizoma Et Radix] 10 g, Glycyrrhiza glabra L. [Fabaceae; Glycyrrhizae Radix et Rhizoma] 6 g | 1) Excessive phlegm: add Fritillaria cirrhosa D.Don [Liliaceae; Fritillariae Cirrhosae Bulbus], Gleditsia sinensis Lam. [Fabaceae; Gleditsiae Spina] 2) Sore throat: add Melicope pteleifolia (Champ. ex Benth.) T.G.Hartley [Rutaceae; Melicope pteleifolia], Centella asiatica (L.) Urb. [Apiaceae; Herba Centellae] 3) Red tongue and white fur: add Asarum heterotropoides F.Schmidt [Aristolochiaceae; Asiasari Radix et Rhizoma], Perilla frutescens (L.) Britton [Lamiaceae; Perillae Folium] | NA | None | None | 3 weeks | 3 months |
| Xu 2017 | Bufei-tang | Decoction | Astragalus mongholicus Bunge [Fabaceae; Astragali Radix] 20 g, Cullen corylifolium (L.) Medik. [Fabaceae; Psoraleae Semen] 15, g Salvia miltiorrhiza Bunge [Lamiaceae; Salviae Miltiorrhizae Radix] 15 g, Codonopsis pilosula (Franch.) Nannf. [Campanulaceae; Codonopsis Pilosulae Radix] 10 g, Citrus × aurantium f. deliciosa (Ten.) M.Hiroe [Rutaceae; Citri Unshius Pericarpium] 10 g, Morus alba L. [Moraceae; Mori Radicis Cortex] 10 g, Stemona tuberosa Lour. [Stemonaceae; Stemonae Radix] 10 g | NA | NA | None | None | 4 weeks | None |
| Yan 2018 | Yupingfeng-san | Powder | Astragalus mongholicus Bunge [Fabaceae; Astragali Radix], Atractylodes lancea (Thunb.) DC. [Asteraceae; Atractylodis Rhizoma], Saposhnikovia divaricata (Turcz. ex Ledeb.) Schischk. [Apiaceae; Saposhnikoviae Radix] | NA | Guangdong Universal Pharmaceutical Co. | None | None | 2 weeks | None |
| Yang 2012 | Jiangxin Huatan Zhike-fang | Decoction | Zingiber officinale Roscoe [Zingiberaceae; Zingiberis Rhizoma] 15-30 g, Asarum heterotropoides F.Schmidt [Aristolochiaceae; Asiasari Radix et Rhizoma] 3 g, Pinellia ternata (Thunb.) Makino [Araceae; Pinelliae Tuber] 10 g, Arisaema erubescens (Wall.) Schott [Araceae; Arisaematis Rhizoma] 9 g, Sinapis alba L. [Brassicaceae; Sinapis Semen Alba] 10 g, Aster tataricus L.f. [Asteraceae; Asteris Radix et Rhizoma] 10 g, Tussilago farfara L. [Asteraceae; Farfarae Flos] 10 g, Inula japonica Thunb. [Asteraceae; Inulae Flos] 10 g, Pseudostellaria heterophylla (Miq.) Pax [Caryophyllaceae; Pseudostellariae Radix] 15 g | 1) Sinusitis: add Angelica dahurica (Hoffm.) Benth. & Hook.f. ex Franch. & Sav. [Apiaceae; Angelicae Dahuricae Radix] 10 g, Lonicera japonica Thunb. [Caprifoliaceae; Lonicerae Flos] 15 g, Forsythia suspensa (Thunb.) Vahl [Oleaceae; Forsythiae Fructus] 15 g, Scutellaria baicalensis Georgi [Lamiaceae; Scutellariae Radix] 10 g 2) Belching and acid regurgitation: add Citrus trifoliata L. [Rutaceae; Ponciri Fructus Immaturus] 12 g, Areca catechu L. [Arecaceae; Arecae Pericarpium] 12 g, Scapharca subcrenata (Lischke) [Arcidae; Scapharcae seu Tegillarcae Concha] 20 g 3) Night cough: add Ginkgo biloba L. [Ginkgoaceae; Ginkgonis Semen] 9 g, Galanthus nivalis L. [Amaryllidaceae; Lumbricus] 10 g | NA | None | None | 10 days | None |
| Yang 2019 | Qingfei Runzao-fang | Decoction | Morus alba L. [Moraceae; Mori Folium] 15 g, Prunus armeniaca L. [Rosaceae; Armeniacae Semen] 10 g, Kitagawia praeruptora (Dunn) Pimenov [Apiaceae; Peucedani Radix] 10 g, Fritillaria thunbergii Miq. [Liliaceae; Fritillariae Thunbergii Bulbus] 10 g, Forsythia suspensa (Thunb.) Vahl [Oleaceae; Forsythiae Fructus] 15 g, Stemona tuberosa Lour. [Stemonaceae; Stemonae Radix] 10 g, Angelica gigas Nakai [Apiaceae; Angelicae Gigantis Radix] 10 g, Arctium lappa L. [Asteraceae; Arctii Fructus] 10 g, Phragmites australis (Cav.) Trin. ex Steud. [Poaceae; Phragmitis Rhizoma] 30 g, Lilium lancifolium Thunb. [Liliaceae; Lilii Bulbus] 20 g, Platycodon grandiflorus (Jacq.) A.DC. [Campanulaceae; Platycodonis Radix] 10 g, Rhaphiolepis bibas (Lour.) Galasso & Banfi [Rosaceae; Eriobotryae Folium] 10 g, Glycyrrhiza glabra L. [Fabaceae; Glycyrrhizae Radix et Rhizoma] 6 g | 1) Severe fluid loss: add Glehnia littoralis (A.Gray) F.Schmidt ex Miq. [Apiaceae; Glehniae Radix], Ophiopogon japonicus (Thunb.) Ker Gawl. [Asparagaceae; Liriopis seu Ophiopogonis Tuber] 2) Phlegm sticky hard to come out: add Aster tataricus L.f. [Asteraceae; Asteris Radix et Rhizoma], Trichosanthes kirilowii Maxim. [Cucurbitaceae; Trichosanthis Semen] 3) Blood in the phlegm: add Rehmannia glutinosa (Gaertner) Liboschitz ex Steudel [Scrophulariaceae; Rehmanniae Radix Recens], Imperata cylindrica (L.) Raeusch. [Poaceae; Imperatae Rhizoma] | NA | None | None | 2 weeks | None |
| Yang 2020 | Xuanfei Zhisou-tang | Granule | Ephedra sinica Stapf [Ephedraceae; Ephedrae Herba] 10 g, Prunus armeniaca L. [Rosaceae; Armeniacae Semen] 10 g, Scrophularia ningpoensis Hemsl. [Scrophulariaceae; Scrophulariae Radix] 15 g, Platycodon grandiflorus (Jacq.) A.DC. [Campanulaceae; Platycodonis Radix] 10 g, Sesamum indicum L. [Pedaliaceae; Schizonepetae Spica] 10 g, Aster tataricus L.f. [Asteraceae; Asteris Radix et Rhizoma] 10 g, Tussilago farfara L. [Asteraceae; Farfarae Flos] 10 g, Perilla frutescens (L.) Britton [Lamiaceae; Perillae Fructus] 15 g, Cryptotympana dubia (Haupt) [Cicadidae; Cicadidae Periostracum] 5 g, Kitagawia praeruptora (Dunn) Pimenov [Apiaceae; Peucedani Radix] 10 g, Arctium lappa L. [Asteraceae; Arctii Fructus] 15 g, Schisandra chinensis (Turcz.) Baill. [Schisandraceae; Schisandrae Fructus] 5 g, Glycyrrhiza glabra L. [Fabaceae; Glycyrrhizae Radix et Rhizoma] 10 g | NA | Jiangyin Tianjiang Pharmaceutical Co., Ltd. | None | None | 2 weeks | None |
| Yang 2022 | Shansan-tang | Decoction | Ophiopogon japonicus (Thunb.) Ker Gawl. [Asparagaceae; Liriopis seu Ophiopogonis Tuber] 10-15 g, Poria cocos Wolf [Polyporaceae; Poria Sclerotium] 10-15 g, Scrophularia ningpoensis Hemsl. [Scrophulariaceae; Scrophulariae Radix] 5-10 g, Asparagus cochinchinensis (Lour.) Merr. [Asparagaceae; Asparagi Tuber] 5-10 g, Perilla frutescens (L.) Britton [Lamiaceae; Perillae Folium] 3-6 g, Fritillaria thunbergii Miq. [Liliaceae; Fritillariae Thunbergii Bulbus] 10-15 g, Glycyrrhiza glabra L. [Fabaceae; Glycyrrhizae Radix et Rhizoma] 3-6 g, Tussilago farfara L. [Asteraceae; Farfarae Flos] 3-6 g, Scutellaria baicalensis Georgi [Lamiaceae; Scutellariae Radix] 5-10 g | 1) Excessive, thick and white sputum: add Pinellia ternata (Thunb.) Makino [Araceae; Pinelliae Tuber] 3-6 g 2) Sticky stool: add Atractylodes lancea (Thunb.) DC. [Asteraceae; Atractylodis Rhizoma] 3-6 g 3) Vomiting when coughing: add Rhaphiolepis bibas (Lour.) Galasso & Banfi [Rosaceae; Eriobotryae Folium] 3-6 g 4) Obvious rhinorrhea: add Acorus gramineus Aiton [Acoraceae; Acori Graminei Rhizoma] 3-6 g, Angelica dahurica (Hoffm.) Benth. & Hook.f. ex Franch. & Sav. [Apiaceae; Angelicae Dahuricae Radix] 3-6 g | NA | None | None | 1 week | None |
| Yi 2020 | Shashen Yuzhu-tang | Decoction | Adenophora triphylla (Thunb.) A.DC. [Campanulaceae; Adenophorae Radix] 30 g, Polygonatum odoratum (Mill.) Druce [Asparagaceae; Polygonati Odorati Rhizoma] 15 g, Dendrobium nobile Lindl. [Orchidaceae; Herba Dendrobii] 12 g, Morus alba L. [Moraceae; Mori Folium] 15 g, The peel of rosaceous plant Pyrus bretschneideri, Nashi Pear or Pyrus ussuriensis Maxim etc [Pericarpium Pyri] 10 g, Glycyrrhiza glabra L. [Fabaceae; Glycyrrhizae Radix et Rhizoma] 6 g | 1) Excessive phlegm and chest tightness: add Perilla frutescens (L.) Britton [Lamiaceae; Perillae Fructus], Raphanus raphanistrum subsp. sativus (L.) Domin [Brassicaceae; Raphani Semen], Sinapis alba L. [Brassicaceae; Sinapis Semen Alba] 2) Severe cough: add Fritillaria cirrhosa D.Don [Liliaceae; Fritillariae Cirrhosae Bulbus], Prunus armeniaca L. [Rosaceae; Armeniacae Semen], Stemona tuberosa Lour. [Stemonaceae; Stemonae Radix] | NA | None | None | 4 weeks | 4 weeks |
| Yu 2014 | NR | Decoction | 1) Loss of lung circulation and liver stagnation: Bupleurum falcatum L. [Apiaceae; Bupleuri Radix] 6 g, Perilla frutescens (L.) Britton [Lamiaceae; Caulis Perillae] 5 g, Platycodon grandiflorus (Jacq.) A.DC. [Campanulaceae; Platycodonis Radix] 5 g, Curcuma longa L. [Zingiberaceae; Curcumae Radix] 10 g, Morus alba L. [Moraceae; Mori Folium] 10 g, Prunus armeniaca L. [Rosaceae; Armeniacae Semen] 10 g, Citrus × aurantium f. deliciosa (Ten.) M.Hiroe [Rutaceae; Citri Unshius Pericarpium] 10 g, Oroxylum indicum (L.) Kurz [Bignoniaceae; Semen Oroxyli] 3 g 2) Liver fire attack lung: Gazella subgutturosa (Guldenstaedt) [Bovidae; Gazellae seu Saigae Cornu] 0.3 g, Nardotidis seu Sulculii Concha 10 g, Scutellaria baicalensis Georgi [Lamiaceae; Scutellariae Radix] 10 g, Lycium barbarum L. [Solanaceae; Lycii Radicis Cortex] 10 g, Rhaphiolepis bibas (Lour.) Galasso & Banfi [Rosaceae; Eriobotryae Folium] 6 g 3) Liver qi ascending counterflow and lung heat: Gentiana scabra Bunge [Gentianaceae; Gentianae Scabrae Radix et Rhizoma] 10 g, Scutellaria baicalensis Georgi [Lamiaceae; Scutellariae Radix] 12 g, Morus alba L. [Moraceae; Mori Radicis Cortex] 12 g, Lycium barbarum L. [Solanaceae; Lycii Radicis Cortex] 12 g, Rehmannia glutinosa (Gaertner) Liboschitz ex Steudel [Scrophulariaceae; Rehmanniae Radix Recens] 12 g, Paeonia × suffruticosa Andrews [Paeoniaceae; Moutan Radicis Cortex] 12 g | 1) Cough with excessive, white and sticky sputum: add Citrus × aurantium f. deliciosa (Ten.) M.Hiroe [Rutaceae; Citri Unshius Pericarpium] 6 g, Pinellia ternata (Thunb.) Makino [Araceae; Pinelliae Tuber] 6 g, Aster tataricus L.f. [Asteraceae; Asteris Radix et Rhizoma] 6 g, Poria cocos Wolf [Polyporaceae; Poria Sclerotium] 10 g, Glycyrrhiza glabra L. [Fabaceae; Glycyrrhizae Radix et Rhizoma] 3 g 2) Cough with yellow sputum: add Morus alba L. [Moraceae; Mori Radicis Cortex] 10 g, Lycium barbarum L. [Solanaceae; Lycii Radicis Cortex] 10 g, Descurainia sophia (L.) Webb ex Prantl [Brassicaceae; Lepidii seu Descurainiae Semen] 10 g, Perilla frutescens (L.) Britton [Lamiaceae; Perillae Fructus] 10 g, Tussilago farfara L. [Asteraceae; Farfarae Flos] 10 g, Fritillaria cirrhosa D.Don [Liliaceae; Fritillariae Cirrhosae Bulbus] 3 g 3) Dry stool: add Raphanus raphanistrum subsp. sativus (L.) Domin [Brassicaceae; Raphani Semen] 10 g, Hordeum vulgare L. [Poaceae; Hordei Fructus Germinatus] 10 g, Trichosanthes kirilowii Maxim. [Cucurbitaceae; Trichosanthis Semen] 10 g | NA | None | None | 20 days | None |
| Zhang 2016 | NR | Decoction | Ephedra sinica Stapf [Ephedraceae; Ephedrae Herba] 15 g, Prunus armeniaca L. [Rosaceae; Armeniacae Semen] 15 g, Ardisia japonica (Thunb.) Blume [Primulaceae; Herba Ardisiae Japonicae] 15 g, Adenophora triphylla (Thunb.) A.DC. [Campanulaceae; Adenophorae Radix] 15 g, Glycyrrhiza glabra L. [Fabaceae; Glycyrrhizae Radix et Rhizoma] 9 g | NA | NA | None | None | 2 weeks | None |
| Zhang 2020 | Chai Huang granule | Granule | Bupleurum falcatum L. [Apiaceae; Bupleuri Radix], Scutellaria baicalensis Georgi [Lamiaceae; Scutellariae Radix] | NA | Sichuan Baili Pharmaceutical Group | None | None | 2 weeks | None |
| Zhang 2021 | Ganmai Dazao-tang combined with Zhizishi-tang | Decoction | Triticum aestivum L. [Poaceae; Tritici Fructus Levis] 30 g, Glycyrrhiza glabra L. [Fabaceae; Glycyrrhizae Radix et Rhizoma] 20 g, Ziziphus jujuba Mill. [Rhamnaceae; Zizyphi Fructus] 20 pieces, Gardenia jasminoides J.Ellis [Rubiaceae; Gardeniae Fructus] 10 g, Glycine max Merrill [Leguminosae; Glycine Semen Preparata] 10 g | NA | NA | None | None | 4 weeks | None |
| Zhang 2022 | Maxing Shigan-tang | Decoction | Gypsum Fibrosum 20 g, Prunus armeniaca L. [Rosaceae; Armeniacae Semen] 10 g, Ephedra sinica Stapf [Ephedraceae; Ephedrae Herba] 8 g, Glycyrrhiza glabra L. [Fabaceae; Glycyrrhizae Radix et Rhizoma] 6 g | 1) Gastroesophageal reflux: add Scapharca subcrenata (Lischke) [Arcidae; Scapharcae seu Tegillarcae Concha] 15 g, Sepiella maindroni de Rochebrune [Sepiidae; Sepiae Endoconcha] 15 g 2) Heat in the upper energizer: add Oroxylum indicum (L.) Kurz [Bignoniaceae; Semen Oroxyli] 3 g, Scutellaria baicalensis Georgi [Lamiaceae; Scutellariae Radix] 9 g 3) Yin deficiency: add Adenophora triphylla (Thunb.) A.DC. [Campanulaceae; Adenophorae Radix] 9 g, Ophiopogon japonicus (Thunb.) Ker Gawl. [Asparagaceae; Liriopis seu Ophiopogonis Tuber] 9 g 4) Severe cough: add Tussilago farfara L. [Asteraceae; Farfarae Flos] 9 g, Schisandra chinensis (Turcz.) Baill. [Schisandraceae; Schisandrae Fructus] 9 g, Kitagawia praeruptora (Dunn) Pimenov [Apiaceae; Peucedani Radix] 6 g 5) Mixed with phlegm and dampness: add Wurfbainia compacta (Sol. ex Maton) Škorničk. & A.D.Poulsen [Zingiberaceae; Amomi Fructus Rotundus] 9 g, Coix lacryma-jobi var. ma-yuen (Rom.Caill.) Stapf [Poaceae; Coicis Semen] 12 g, Pinellia ternata (Thunb.) Makino [Araceae; Pinelliae Tuber] 12 g 6) Hard to sleep: add Poria cocos Wolf [Polyporaceae; Poria Sclertum Cum Pini Radix] 12 g, Reynoutria multiflora (Thunb.) Moldenke [Polygonaceae; Polygoni Multiflori Caulis] 12 g | NA | None | None | 1 week | None |
| Zhao 2022a | Liujunzi-tang | Decoction | Codonopsis pilosula (Franch.) Nannf. [Campanulaceae; Codonopsis Pilosulae Radix] 10 g, Atractylodes lancea (Thunb.) DC. [Asteraceae; Atractylodis Rhizoma] 12 g, Poria cocos Wolf [Polyporaceae; Poria Sclerotium] 12 g, Glycyrrhiza glabra L. [Fabaceae; Glycyrrhizae Radix et Rhizoma] 6 g, Citrus × aurantium f. deliciosa (Ten.) M.Hiroe [Rutaceae; Citri Unshius Pericarpium] 3 g, Pinellia ternata (Thunb.) Makino [Araceae; Pinelliae Tuber] 4.5 g | NA | NA | None | None | 1 week | 1 week |
| Zhao 2022b | Xuanfei Yunpi-tang | Decoction | Scutellaria baicalensis Georgi [Lamiaceae; Scutellariae Radix] 9 g, Citrus × aurantium f. deliciosa (Ten.) M.Hiroe [Rutaceae; Citri Unshius Pericarpium] 12 g, Platycodon grandiflorus (Jacq.) A.DC. [Campanulaceae; Platycodonis Radix] 6 g, Perilla frutescens (L.) Britton [Lamiaceae; Perillae Folium] 9 g, Fritillaria thunbergii Miq. [Liliaceae; Fritillariae Thunbergii Bulbus] 12 g, Rehmannia glutinosa (Gaertn.) DC. [Orobanchaceae; Rehmanniae Radix Preparata] 12 g, Codonopsis pilosula (Franch.) Nannf. [Campanulaceae; Codonopsis Pilosulae Radix] 9 g, Astragalus mongholicus Bunge [Fabaceae; Astragali Radix] 15 g, Morus alba L. [Moraceae; Mori Radicis Cortex] 12 g, Poria cocos Wolf [Polyporaceae; Poria Sclerotium] 9 g, Amomum villosum Loureiro var. xanthioides T. L. Wu et Senjen [Zingiberaceae; Amomi Fructus] 6 g, Glycyrrhiza glabra L. [Fabaceae; Glycyrrhizae Radix et Rhizoma] 6 g, Trichosanthes kirilowii Maxim. [Cucurbitaceae; Trichosanthis Semen] 12 g, Pinellia ternata (Thunb.) Makino [Araceae; Pinelliae Tuber] 9 g, Atractylodes lancea (Thunb.) DC. [Asteraceae; Atractylodis Rhizoma] 9 g | NA | NA | None | None | 10 days | None |
| Zhao 2022c | Shegan Mahuang-tang | Decoction | Iris domestica (L.) Goldblatt & Mabb. [Iridaceae; Belamcandae Rhizoma] 5 g, Ephedra sinica Stapf [Ephedraceae; Ephedrae Herba] 5 g, Schisandra chinensis (Turcz.) Baill. [Schisandraceae; Schisandrae Fructus] 5 g, Aster tataricus L.f. [Asteraceae; Asteris Radix et Rhizoma] 5 g, Tussilago farfara L. [Asteraceae; Farfarae Flos] 5 g, Asarum heterotropoides F.Schmidt [Aristolochiaceae; Asiasari Radix et Rhizoma] 0.5 g, Zingiber officinale Roscoe [Zingiberaceae; Zingiberis Rhizoma] 5 g, Pinellia ternata (Thunb.) Makino [Araceae; Pinelliae Tuber] 3 g, Glycyrrhiza glabra L. [Fabaceae; Glycyrrhizae Radix et Rhizoma] 3 g | 1) Dry stool: add Cannabis sativa L. [Cannabaceae; Cannabis Semen] 5 g 2) Poor food intake: add Dolomiaea costus (Falc.) Kasana & A.K.Pandey [Asteraceae; Aucklandiae Radix] 5 g, Wurfbainia compacta (Sol. ex Maton) Škorničk. & A.D.Poulsen [Zingiberaceae; Amomi Fructus Rotundus] 5 g 3) Diarrhea: add Atractylodes lancea (Thunb.) DC. [Asteraceae; Atractylodis Rhizoma] 5 g, Poria cocos Wolf [Polyporaceae; Poria Sclerotium] 5 g 4) Severe asthma: add Ziziphus jujuba Mill. [Rhamnaceae; Zizyphi Fructus] 2 pieces 5) Fever: add Bupleurum falcatum L. [Apiaceae; Bupleuri Radix] 5 g | NA | None | None | 2 weeks | None |
| Zhou 2011 | Yupingfeng-san | Decoction | Astragalus mongholicus Bunge [Fabaceae; Astragali Radix] 10-15 g, Atractylodes lancea (Thunb.) DC. [Asteraceae; Atractylodis Rhizoma] 5-10 g, Perilla frutescens (L.) Britton [Lamiaceae; Perillae Fructus] 5-10 g, Ophiopogon japonicus (Thunb.) Ker Gawl. [Asparagaceae; Liriopis seu Ophiopogonis Tuber] 5-10 g, Kitagawia praeruptora (Dunn) Pimenov [Apiaceae; Peucedani Radix] 5-10 g, Aster tataricus L.f. [Asteraceae; Asteris Radix et Rhizoma] 5-10 g, Saposhnikovia divaricata (Turcz. ex Ledeb.) Schischk. [Apiaceae; Saposhnikoviae Radix] 3-6 g, Platycodon grandiflorus (Jacq.) A.DC. [Campanulaceae; Platycodonis Radix] 3-6 g, Glycyrrhiza glabra L. [Fabaceae; Glycyrrhizae Radix et Rhizoma] 3-5 g | 1) Severe cough: add Inula japonica Thunb. [Asteraceae; Inulae Flos] 5-10g, Pinellia ternata (Thunb.) Makino [Araceae; Pinelliae Tuber] 5-10 g 2) Cough with yellow phlegm: add Fritillaria cirrhosa D.Don [Liliaceae; Fritillariae Cirrhosae Bulbus] 5-10 g, Rhaphiolepis bibas (Lour.) Galasso & Banfi [Rosaceae; Eriobotryae Folium] 5-10 g | NA | None | None | 2 weeks | None |
| Zhou 2017 | Xuanshen Shengma-tang | Decoction | Arctium lappa L. [Asteraceae; Arctii Fructus] 12 g, Sophora tonkinensis var. tonkinensis [Fabaceae; Sophorae Subprostratae Radix] 6 g, Actaea racemosa L. [Ranunculaceae; Cimicifugae Rhizoma] 6 g, Stemona tuberosa Lour. [Stemonaceae; Stemonae Radix] 9 g, Scrophularia ningpoensis Hemsl. [Scrophulariaceae; Scrophulariae Radix] 9 g, Isatis tinctoria L. [Brassicaceae; Isatidis Radix] 15 g, Rhizoma Paridis Verticillatae 9 g, Scutellaria baicalensis Georgi [Lamiaceae; Scutellariae Radix] 9 g, Trichosanthes kirilowii Maxim. [Cucurbitaceae; Trichosanthis Semen] 9 g, Platycodon grandiflorus (Jacq.) A.DC. [Campanulaceae; Platycodonis Radix] 6 g, Daiha-san 9 g, Fritillaria thunbergii Miq. [Liliaceae; Fritillariae Thunbergii Bulbus] 9 g, Aster tataricus L.f. [Asteraceae; Asteris Radix et Rhizoma] 9 g, Iris domestica (L.) Goldblatt & Mabb. [Iridaceae; Belamcandae Rhizoma] 9 g, Alkekengi officinarum var. franchetii (Mast.) R.J.Wang [Solanaceae; Physalis alkekengi L. var. franchetii] 6 g | 1) Inner heat: add Imperata cylindrica (L.) Raeusch. [Poaceae; Imperatae Rhizoma] 9 g  2) Excessive phlegm: add Pumex 9 g, Vincetoxicum stauntonii (Decne.) C.Y.Wu & D.Z.Li [Apocynaceae; Cynanchi Stauntonii Rhizoma Et Radix] 6 g, Houttuynia cordata Thunb. [Saururaceae; Houttuyniae Herba] 6 g  3) Severe cough: add Terminalia chebula Retz. [Combretaceae; Terminaliae Fructus] 6 g | NA | None | None | 2 weeks | None |
| Zhou 2018 | Zhisou-san | Decoction | Stemona tuberosa Lour. [Stemonaceae; Stemonae Radix] 12 g, Aster tataricus L.f. [Asteraceae; Asteris Radix et Rhizoma] 12 g, Vincetoxicum stauntonii (Decne.) C.Y.Wu & D.Z.Li [Apocynaceae; Cynanchi Stauntonii Rhizoma Et Radix] 12 g, Platycodon grandiflorus (Jacq.) A.DC. [Campanulaceae; Platycodonis Radix] 12 g, Sesamum indicum L. [Pedaliaceae; Schizonepetae Spica] 8 g, Citrus × aurantium f. deliciosa (Ten.) M.Hiroe [Rutaceae; Citri Unshius Pericarpium] 6 g, Glycyrrhiza glabra L. [Fabaceae; Glycyrrhizae Radix et Rhizoma] 6 g | 1) Headache, nasal congestion, aversion to cold: add Saposhnikovia divaricata (Turcz. ex Ledeb.) Schischk. [Apiaceae; Saposhnikoviae Radix], Zingiber officinale Roscoe [Zingiberaceae; Zingiberis Rhizoma] 2) Stick phlegm, heavy dampness: add Morus alba L. [Moraceae; Mori Radicis Cortex], Poria cocos Wolf [Polyporaceae; Poria Sclerotium], Pinellia ternata (Thunb.) Makino [Araceae; Pinelliae Tuber] 3) Laryngeal phlegm sound: add Iris domestica (L.) Goldblatt & Mabb. [Iridaceae; Belamcandae Rhizoma] 4) Cough with little phlegm: add Fritillaria cirrhosa D.Don [Liliaceae; Fritillariae Cirrhosae Bulbus], Trichosanthes kirilowii Maxim. [Cucurbitaceae; Trichosanthis Semen], Anemarrhena asphodeloides Bunge [Asparagaceae; Anemarrhenae Rhizoma] 5) Thirsty and vexation: add Scutellaria baicalensis Georgi [Lamiaceae; Scutellariae Radix], Coptis chinensis Franch. [Ranunculaceae; Coptidis Rhizoma], Trichosanthes kirilowii Maxim. [Cucurbitaceae; Trichosanthis Radix] | NA | None | None | 4 weeks | None |
| Zhou 2021 | Qinggan Ningfei-fang | Decoction | Persicaria tinctoria (Aiton) Spach [Polygonaceae; Indigo Pulverata Levis] 9 g, Cryptotympana dubia (Haupt) [Cicadidae; Cicadidae Periostracum] 9 g, Bombyx mori (Linné) [Bombycidae; Batryticatus Bombyx] 9 g, Ephedra sinica Stapf [Ephedraceae; Ephedrae Herba] 9 g, Trichosanthes kirilowii Maxim. [Cucurbitaceae; Trichosanthis Pericarpium] 12 g, Prunus armeniaca L. [Rosaceae; Armeniacae Semen] 12 g, Descurainia sophia (L.) Webb ex Prantl [Brassicaceae; Lepidii seu Descurainiae Semen] 12 g, Fritillaria thunbergii Miq. [Liliaceae; Fritillariae Thunbergii Bulbus] 12 g, Pumex Preparata 12 g, Gardenia jasminoides J.Ellis [Rubiaceae; Gardeniae Fructus] 12 g, Bupleurum falcatum L. [Apiaceae; Bupleuri Radix] 12 g, Terminalia chebula Retz. [Combretaceae; Terminaliae Fructus] 12 g | NA | Jiangsu Kangmei Pharmaceutical Co., Ltd. | None | None | 1 month | None |
| Zhou 2022 | Linggan Wuwei Jiangxin-tang combined with Zhisou-san | Decoction | Zingiber officinale Roscoe [Zingiberaceae; Zingiberis Rhizoma] 10 g, Poria cocos Wolf [Polyporaceae; Poria Sclerotium] 10 g, Glycyrrhiza glabra L. [Fabaceae; Glycyrrhizae Radix et Rhizoma] 10 g, Schisandra chinensis (Turcz.) Baill. [Schisandraceae; Schisandrae Fructus] 10 g, Platycodon grandiflorus (Jacq.) A.DC. [Campanulaceae; Platycodonis Radix] 10 g, Aster tataricus L.f. [Asteraceae; Asteris Radix et Rhizoma] 10 g, Citrus × aurantium f. deliciosa (Ten.) M.Hiroe [Rutaceae; Citri Unshius Pericarpium] 10 g, Pinellia ternata (Thunb.) Makino [Araceae; Pinelliae Tuber] 10 g, Vincetoxicum stauntonii (Decne.) C.Y.Wu & D.Z.Li [Apocynaceae; Cynanchi Stauntonii Rhizoma Et Radix] 15 g, Asarum heterotropoides F.Schmidt [Aristolochiaceae; Asiasari Radix et Rhizoma] 3 g | 1) Severe symptoms according to clinical syndrome: add Prunus armeniaca L. [Rosaceae; Armeniacae Semen], Ephedra sinica Stapf [Ephedraceae; Ephedrae Herba], Magnolia officinalis Rehder & E.H.Wilson [Magnoliaceae; Magnoliae Cortex], Saposhnikovia divaricata (Turcz. ex Ledeb.) Schischk. [Apiaceae; Saposhnikoviae Radix], Astragalus mongholicus Bunge [Fabaceae; Astragali Radix] | NA | The dosages are within the scope specified in the Chinese Pharmacopoeia | None | 1 month | 1 year |
| Zhu 2012 | Shashen Maidong-tang | Decoction | Glehnia littoralis (A.Gray) F.Schmidt ex Miq. [Apiaceae; Glehniae Radix] 20 g, Ophiopogon japonicus (Thunb.) Ker Gawl. [Asparagaceae; Liriopis seu Ophiopogonis Tuber] 15 g, Polygonatum odoratum (Mill.) Druce [Asparagaceae; Polygonati Odorati Rhizoma] 15 g, Trichosanthes kirilowii Maxim. [Cucurbitaceae; Trichosanthis Radix] 20 g, Lablab purpureus subsp. purpureus [Fabaceae; Dolichoris Semen] 15 g, Morus alba L. [Moraceae; Mori Folium] 15 g, Glycyrrhiza glabra L. [Fabaceae; Glycyrrhizae Radix et Rhizoma] 10 g | 1) Severe dryness and heat in the lungs: add Lycium barbarum L. [Solanaceae; Lycii Radicis Cortex], Artemisia annua L. [Asteraceae; Artemisiae Annuae Herba] 2) Cough louder: add Fritillaria cirrhosa D.Don [Liliaceae; Fritillariae Cirrhosae Bulbus], Prunus armeniaca L. [Rosaceae; Armeniacae Semen], Stemona tuberosa Lour. [Stemonaceae; Stemonae Radix]  3) Lung qi is not restrained, coughing and shortness of breath: add Schisandra chinensis (Turcz.) Baill. [Schisandraceae; Schisandrae Fructus], Terminalia chebula Retz. [Combretaceae; Terminaliae Fructus] 4) Low fever, vexing heat in the chest, palms and soles, afternoon tidal fever: add Stellariae seu Gypsophilae Radix, Lycium barbarum L. [Solanaceae; Lycii Radicis Cortex], Artemisia annua L. [Asteraceae; Artemisiae Annuae Herba]  5) Lung yin deficiency, night sweating: add Oryza sativa L. var. glutinosa Matsumura [Gramineae; Oryzae Rhizoma et Radix], Triticum aestivum L. [Poaceae; Tritici Fructus Levis] 6) Coughing up yellow phlegm: add Meretrix meretrix Linné [Veneridae; Meretricis Concha], Anemarrhena asphodeloides Bunge [Asparagaceae; Anemarrhenae Rhizoma], Scutellaria baicalensis Georgi [Lamiaceae; Scutellariae Radix] 7) Blood in sputum: add Paeonia × suffruticosa Andrews [Paeoniaceae; Moutan Radicis Cortex], Gardenia jasminoides J.Ellis [Rubiaceae; Gardeniae Fructus], Nelumbo nucifera Gaertn. [Nelumbonaceae; Nelumbinis Rhizomatis Nodus] | NA | None | None | 4 weeks | None |
| Zhu 2017 | Qingzao Jiufei-tang | Decoction | Morus alba L. [Moraceae; Mori Folium] 18 g, Gypsum Fibrosum 9 g, Ophiopogon japonicus (Thunb.) Ker Gawl. [Asparagaceae; Liriopis seu Ophiopogonis Tuber] 9 g, Panax ginseng C.A.Mey. [Araliaceae; Ginseng Radix] 6 g, Sesamum indicum L. [Pedaliaceae; Sesami Semen] 12 g, Equus asinus Linne [Equidae; Asini Corii Colla] 6 g, Prunus armeniaca L. [Rosaceae; Armeniacae Semen] 12 g, Rhaphiolepis bibas (Lour.) Galasso & Banfi [Rosaceae; Eriobotryae Folium] 15 g, Buthus martensii Karsch [Buthidae; Scorpio] 6 g, Bombyx mori (Linné) [Bombycidae; Batryticatus Bombyx] 9 g, Glycyrrhiza glabra L. [Fabaceae; Glycyrrhizae Radix et Rhizoma] 6 g | 1) Fever: add Houttuynia cordata Thunb. [Saururaceae; Houttuyniae Herba] 12 g, Scutellaria baicalensis Georgi [Lamiaceae; Scutellariae Radix] 10 g 2) Severe dampness: add Atractylodes lancea (Thunb.) DC. [Asteraceae; Atractylodis Rhizoma] 12 g, Poria cocos Wolf [Polyporaceae; Poria Sclerotium] 12 g 3) Severe cold: add Asarum heterotropoides F.Schmidt [Aristolochiaceae; Asiasari Radix et Rhizoma] 3 g, Ephedra sinica Stapf [Ephedraceae; Ephedrae Herba] 6 g 4) Severe dryness: add Lilium lancifolium Thunb. [Liliaceae; Lilii Bulbus] 12 g, Polygonatum sibiricum Redouté [Asparagaceae; Polygonati Rhizoma] 15 g | NA | None | None | 2 weeks | None |

CVA, cough variant asthma; GERD, gastroesophageal reflux disease; NA, not applicable; UACS, upper airway cough syndrome.

**Supplement 5. Risk of bias of the included studies**

| 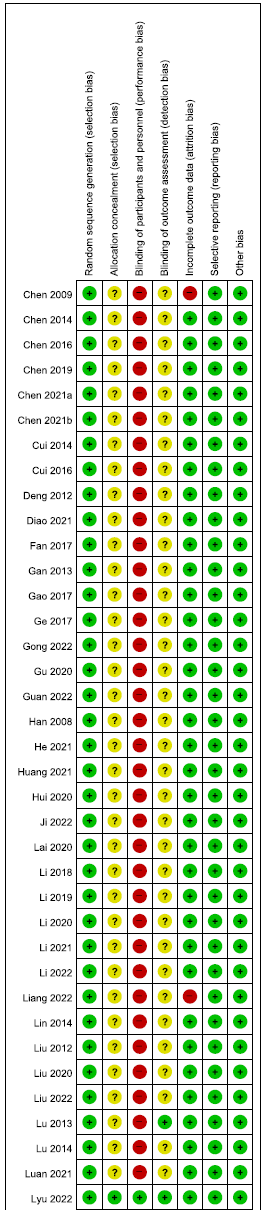 | 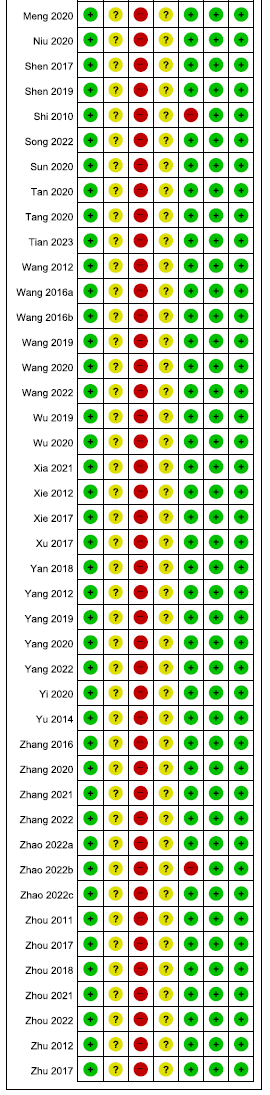 |
| --- | --- |

Low, unclear, and high risk, respectively, are represented with the following symbols: “+”, “?”, and “-”.

**Supplement 6. Funnel plots**

1. HM versus conventional medication: Total effective rate

1. HM versus conventional medication: Incidence of adverse events

1. HM combined with conventional medication versus conventional medication alone: Total effective rate

1. HM combined with conventional medication versus conventional medication alone: Incidence of adverse events
